# Constructing Taxonomies to Identify Distinctive Forms of Primary Healthcare Organizations

**DOI:** 10.5402/2013/798347

**Published:** 2013-04-15

**Authors:** Roxane Borgès Da Silva, Raynald Pineault, Marjolaine Hamel, Jean-Frédéric Levesque, Danièle Roberge, Paul Lamarche

**Affiliations:** ^1^Direction de santé publique de l'agence de la santé et des services sociaux de Montréal, 1301 Sherbrooke Est, Montréal, QC, Canada H2L 1M3; ^2^Institut national de santé publique du Québec, QC, Canada; ^3^Département d'administration de la santé et institut de recherche en santé publique de l'université de Montréal, QC, Canada; ^4^Centre de recherche du centre hospitalier de l'université de Montréal, Montréal, QC, Canada; ^5^Département de médecine familiale, université de Montréal, Montréal, QC, Canada; ^6^Centre de recherche de l'hôpital Charles Lemoyne, Longueuil, QC, Canada; ^7^Université de Sherbrooke, Sherbrooke, QC, Canada

## Abstract

*Background*. Primary healthcare (PHC) renewal gives rise to important challenges for policy makers, managers, and researchers in most countries. Evaluating new emerging forms of organizations is therefore of prime importance in assessing the impact of these policies. This paper presents a set of methods related to the configurational approach and an organizational taxonomy derived from our analysis. *Methods*. In 2005, we carried out a study on PHC in two health and social services regions of Quebec that included urban, suburban, and rural areas. An organizational survey was conducted in 473 PHC practices. We used multidimensional nonparametric statistical methods, namely, multiple correspondence and principal component analyses, and an ascending hierarchical classification method to construct a taxonomy of organizations. *Results*. PHC organizations were classified into five distinct models: four professional and one community. Study findings indicate that the professional integrated coordination and the community model have great potential for organizational development since they are closest to the ideal type promoted by current reforms. *Conclusion*. Results showed that the configurational approach is useful to assess complex phenomena such as the organization of PHC. The analysis highlights the most promising organizational models. Our study enhances our understanding of organizational change in health services organizations.

## 1. Background

Adapting healthcare systems to the changing needs of populations has provided important insights into primary healthcare (PHC) and given rise to political and organizational changes in many countries [1]; Canada is no exception [2–5]. The way PHC services are planned, organized, provided, and funded has been the subject of much questioning. Fragmentation of health services, ineffective use of qualified professionals, poor quality of information sharing tools, lack of coordination, the little importance given to prevention, and lack of access to care are perceived to be the main problems [2–4]. These organizational shortcomings reflect on the care experiences of individuals who have problems with access to services, continuity, and comprehensiveness of care. In a context of reforms, promising organizational models become all the more important. For decision makers who lead and manage organizational changes, evaluating new emerging forms of organizations and organizational changes is therefore of prime importance in assessing the impact of their policy decisions [6]. For researchers and decision makers, the challenges are great. It is relatively easy to evaluate interventions such as therapeutic procedures since their components can be readily identified and characterized. However, it is more difficult to do so at the organizational and system levels since interventions become more complex. The complex nature of organizational phenomena presents specific conceptual and methodological challenges to researchers, in terms of increasing their understanding of organizations and their evolution over time [7, 8]. To be useful, study results need to take into account the complex nature of organizations and the multiple interactions with their contexts.

Over the past decade, studies investigating PHC organizations have looked at a wide variety of organizational attributes that characterize service delivery, identified determinants of efficient healthcare organization, and offered guidance pertaining to organizational changes [9, 10]. Several studies have compared different types of PHC organizations (e.g., Kaiser, Veterans Administration in the United States, and PHC in Ontario) [11–14]. A Québec evaluative study has explored medical services available in private practices and local community health centers (CLSC). CLSC are public clinics, run and funded by the provincial government, in which physicians are paid by salary. Organizational innovations that have resulted from new public policies, such as “Family Medicine Groups” (FMG) in Québec, have also been studied [15–18]. In 2002, Family Medicine Groups (FMG) were created to increase access to family physicians for all, improve the quality of PHC and enhance the role of family physicians. FMG are groups of family physicians who work closely with nurses to offer family medicine services to registered individuals. The results of these studies are particularly insightful when comparing organizations on specific attributes and evaluating the performance of organizations. Studies have highlighted the importance of organizational characteristics such as gatekeeping, group medical practice and multidisciplinarity, longer hours, and integration of services into the health system for the performance of PHC organizations [19–25]. Studies have also shown that providers' characteristics and the professional composition of a healthcare team influence PHC practice and service delivery [19, 26]. Technical resources supporting PHC practice, such as diagnostic technologies and information systems, also influence the delivery of services [19]. Other studies have explored organization governance, methods of funding, and regulatory mechanisms [1, 27]. They have shown that providers' modes of payment affect care delivery, scope of services offered, and productivity [28–30].

Most of these studies have assessed the contribution of individual organizational attributes on the performance of PHC organizations. In so doing, they have overlooked the commonalities among various attributes and fail to look at organizational phenomena in a holistic way [31]. In sum, they tend to simplify highly complex organizational entities, thus limiting their interpretative capacity [32]. A research tradition in organization theory has opened a new perspective by using a systemic approach to design and analysis of health services [33–37]. In these studies, organizations are viewed as indivisible entities rather than sets of properties, and efforts are focused on understanding organizational forms and archetypes [38, 39]. As Meyer et al. put it, the term “organizational configuration denotes any multidimensional constellation of conceptually distinct characteristics that commonly occur together” [33]. The configurational approach takes into account the interdependence of organizational attributes and defines the various organizational forms that shape health systems and their components. Organizational groupings based on organizational configurations or taxonomies have a distinct explanatory potential [17].

According to the configurational perspective, an organization is not merely a set of attributes. It is a living and dynamic entity. There is no single definition of organization. Its meaning varies depending on the theoretical approach adopted [40]. Our perspective is to view organization as a social arrangement and an organized system for collective action, in which activities are planned and coordinated to achieve its mission and attain its goals [41]. To accomplish this mission, an organization requires resources that can be mobilized to produce services and a structure of governance that guides its members in carrying out their activities as well as exchanges with its environments [41]. Attributes of organization can thus be grouped around four domains: (1) a mission that states its goals and orientations; (2) a structure that provides a regulatory and governance framework for action; (3) resources that are required to produce services; and (4) professional and administrative practices embedded in mechanisms that underpin the production and delivery of services [42]. Each of these four domains must develop and maintain exchanges with their specific environments. For example, setting organizational goals must take into account the higher level of a healthcare system's goals. Likewise, sharing resources and collaboration between organizations are necessary to offer a broad range of services. Hence, environments are embedded in the four domains.

We conducted a study on accessibility and continuity of health services in PHC in Québec [43]. These four domains served as a frame of reference for creating the organizational questionnaire used in our study and analyzing the results. To evaluate the organization of PHC, we constructed a taxonomy of PHC organizations. The prime objective of this paper is to present the methods used and the results obtained in developing the taxonomy of PHC organizations. A secondary objective is to demonstrate the usefulness of the configurational approach to evaluate complex phenomena such as the organization of PHC.

## 2. Research Design

**Figure 1:**
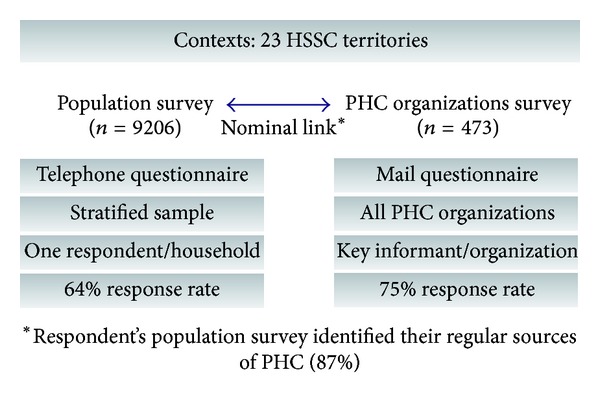
Research design.

The study on accessibility and continuity of PHC was conducted in two health and social services regions of the province of Quebec. The two regions include 23 territories that are located in urban, suburban, and rural areas. As shown in Figure 1, the study design involved two levels of analysis: a PHC organizational survey and a population survey. We conducted a mail organizational survey of general practitioners' PHC practice settings in the two regions (*n* = 665) [44]. The methodological procedures for this part of the study and the results of the analysis are the subject of this paper. The population telephone survey was conducted among 9206 respondents in the two regions. The objective was to gather information about the population's use of regular source of PHC and its perceptions of care accessibility, continuity, comprehensiveness, responsiveness, and outcomes [45]. The results of the population and organization surveys were nominally linked by identifying the regular source of PHC of respondents to the population survey. This paper does not aim to give details of the population survey, and its results that are presented in the research report are available in the Supplementary Material available online at http://dx.doi.org/10.5402/2013/798347 [46]. Only a few selected indicators are presented in this paper.

## 3. Methods

### 3.1. Data Sources

We conducted a mail survey of all PHC practices in the two regions (*n* = 665). Practices included privates offices (solo and groups), FMG, Family Medicine Units (FMU), and physician practices in CLSC (FMU are clinics that includes a medical education component; they are affiliated to university hospitals). One key informant per organization was chosen to answer a self-administered questionnaire. Most of the time, the respondent was a physician in charge of the organization or the one identified as the most knowledgeable regarding the clinic's overall activities. A total of 473 organizations participated in the survey, for a response rate of 71%. All clinical settings were well represented (CLSC: 91%; FMU: 91%; FMG: 90%; and private practices: 70%).

To our knowledge, there was no tool at that time. We thus developed an original questionnaire for this survey that addressed the specific questions we wanted to explore. Construction of the questionnaire and choice of items are based on previous work on health services organization realized by Starfield, Pineault et al., and Haggerty et al. [19, 39, 47]. (The questionnaire is available online at http://www.dsp.santemontreal.qc.ca/fileadmin/documents/dossiers_thematiques/Services_preventifs/ESPSS/questionnaireorgeng.pdf). The questionnaire was tested for face validity to assess the relevance of the questions it contains. It was also tested for content validity to determine the degree of exhaustivity of the questions covering the concepts. As shown in Table 1, organizational attributes are grouped around the four conceptual domains presented earlier: (1) the vision, goals, and orientations adopted by actors, providers, and organizations; (2) structure which includes elements of PHC organization governance, regulations, and agreements; (3) human, financial, and technological resources PHC organizations have at their disposal; and (4) professional and administrative practices that consist in the organizational procedures and mechanisms supporting service production and delivery.

### 3.2. Data Analysis

**Figure 2:**
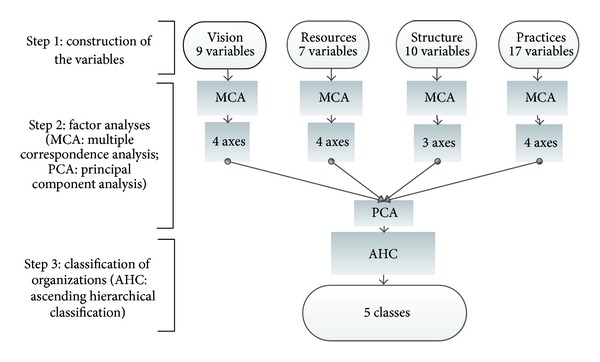
Three-step procedure followed to construct the taxonomy.

The information obtained from the organizational survey enabled us to characterize PHC organizations. Our goal was to establish an organizational classification which would group PHC organizations based on their common characteristics. The organizational data were analyzed using multidimensional non-parametric statistical methods [48, 49]. These methods are relevant because they enable a large amount of information to be processed and synthetized as a coherent whole. Analyses were conducted in three steps (Figure 2).

1. Construction of the variables: the variables were recoded into a restricted number of categories, based on four conceptual domains (vision, structure, resources, and practices), and frequency distributions.
2. Factors analysis: multiple correspondence analyses (MCA) were performed to study relationships between the variables in each of the four conceptual domains. This method is appropriate when the variables are categorical and the relationships between variables are nonlinear. The decision on the number of factorial axes to keep for subsequent analyses was based on the elbow criterion applied to the eigenvalue curve (corresponding to the point of inflection of the curve) [48–50] and on the cumulative inertia (adjusted eigenvalues with the correction of Benzécri for MCA) [51]. The factorial axes that resulted from the MCA were integrated into a principal component analysis (PCA). From PCA, again using the elbow criterion applied to the eigenvalue curve, we selected principal components.
3. Classification of organizations: we used an ascending hierarchical classification method (AHC) [52]. This technique has been shown to be effective in partitioning groups for which the internal variance of each class is minimal and the variance between classes is maximal. This method uses the Ward's generalized criterion. To obtain the classification, we examined the dendrogram which represented the hierarchy tree and the graph of the inertia quotient (interinertia/total inertia). The inertia quotient increases as the number of classes increases, but it tends asymptotically to 1. Therefore, we chose a number of classes for which the inertia quotient does not show any substantial increase. In addition to these statistical considerations, the decision made regarding number of factors to keep takes into consideration the interpretability of the factors [48]. Therefore, the number of partitions used in the final classification took into consideration both the statistical criterion (inertia quotient) and the theoretical and clinical plausibility of the final groupings. All calculations were carried out with using SPAD 7.0 (Data Management-Data Mining; Coheris society) and SPSS 12.0 (Statistical Package for the Social Sciences).

Our method for constructing the taxonomy is based on factorial analysis. MCA and PCA component analyses served to generate factorial axes that were then used in the classification analysis. According to Nakacha and Confais [53], interpretation of the classes of a taxonomy is done by referring back to the variables (and their modalities) that characterize these classes as well as their frequency of occurrence. The contribution of these variables to a class is determined by comparing their frequencies in the class with those in the total population of organizations. For example, taking *P* ≤ .05 as a reference point, the most important explanatory variables are those with a value test greater than 1.96, then reflecting the overrepresentation of this variable in the class and/or its magnitude. The value test corresponds to the difference between its mean in the class and the overall mean expressed in terms of the number of standard deviations.

## 4. Results

In the first step, we recoded 43 categorical variables, grouped into four conceptual domains: vision (9 variables), structure (10 variables), resources (7 variables), and practices (17 variables). The list of variables, their categories, and frequencies and the questions from which they were derived are presented in the appendix in supplementary material.

In the second step of the analysis, we carried out an MCA. For “vision,” we retained four factorial axes (99.29% of cumulative inertia with Benzécri correction). These axes are mainly formed by the following variables: value given to teamwork and attachment to the organization, purpose of services, and level of accountability regarding the healthcare needs of individuals, clienteles, or populations. Four factorial axes were retained for “resources” (99.58% of cumulative inertia with Benzécri correction). These axes are mainly defined by the diversity of resources available for services (medical and nursing services, technical services), clinics' sources of funding, and number of diagnostic and therapeutic procedures available. Three factorial axes were retained for “structure” (99.66% of cumulative inertia with Benzécri correction). These axes are mainly defined by type of governance (public or private), formalization of relationships among the actors in the organization, and links of collaboration with other care providers in the healthcare system. Four factorial axes were retained for “practices” (97.5% of cumulative inertia with Benzécri correction). These axes are primarily defined by quality and quantity of services provided, sharing activities among physicians and visit arrangements (e.g., by appointment/walk-in). PCA was performed on the factorial axes retained from MCA. We choose to keep the first four axes for the classification analysis, which corresponded to 55, 77% of cumulative inertia.

**Figure 3:**
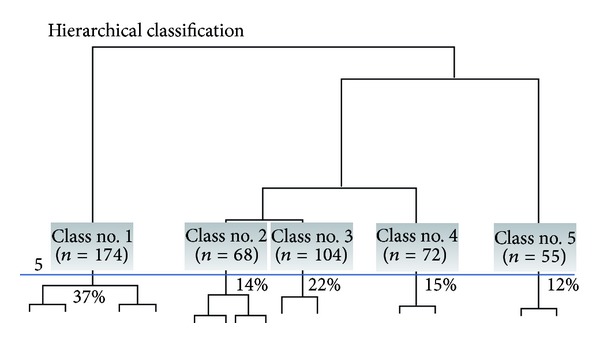
Dendogram (representation of all 473 organizations).

**Figure 4:**
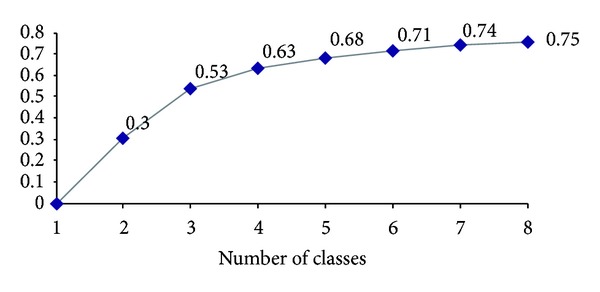
Inertia quotient by number of classes.

In the third step, we grouped the organizations by performing an AHC analysis. The dendogram of the AHC is shown in Figure 3 and the graph of the inertia quotient in Figure 4. As illustrated in Figure 4, any increase in the inertia quotient tends to level off after five partitions. The gains made by adding further classes fail to be substantial. We retained the partition with five classes; this solution can be easily interpreted.

We obtained five organizational PHC models: four *professional *organizational models and one *community* model. In line with the work conducted by Lamarche et al., the difference between professional and community models is primarily structural, stemming from governance and doctors' methods of payment [42]. Professional models attract a majority of PHC organizations in both regions under study (88%). These organizations are designed to deliver medical services to patients who seek these services. With strictly private governance, practitioners are paid on a fee-for-service basis. The findings highlight that these organizational configurations differ markedly by the vision and values of professionals who provide care. Accountability toward clients, organizational priorities, and the importance of teamwork are distinctive organizational characteristics of these models. The underlying bases of these organizations are conveyed not only in the resources and structures available, but also in the practices that ensure service delivery. Indeed, coordination between professionals and institutions, development of interdisciplinary approaches, and scope of services offered are all elements that differentiate professional models one from another. For each of these four models, we chose a term that reflects their dominant characteristics: single provider model, contact model, coordination model, and integrated coordination model. One PHC model corresponds to the community model. It includes 12% of the organizations of the study. These organizations report to a regional or local authority, and remuneration of professionals is time based (salary, sessional fee). The number of organizations by models and their main illustrative characteristics are presented in Table 2.

Over a third of medical clinics in our study fit the *professional single-provider model* (Table 2). Typically, physicians in this model adopt a vision based on the care principles of family medicine, centered on followup of regular clienteles. Secondary analyses on the affiliation of users with a family doctor showed that 94% of users affiliated with this model identify a physician who is responsible for their care (unpublished data). Doctors enjoy a high degree of autonomy. Usually, there is only one doctor in the organization and no nurse; occasionally, two or three physicians share the clinic. Information and clinical support technologies are underdeveloped in these practices. Doctors use the technical services offered by other clinics to support their clinical activities. It is clearly a rudimentary organizational structure. Private professional governance and fee-for-service payment are typical of this model. These clinics have few links with other care providers. Visits usually take place on weekdays by appointment. The scope of services offered is limited, compared to other models.

The *professional contact model* accounts for 14% of the organizations in our study (Table 2). The professional contact model is defined by its role as contact for new health problems and by provision of services to people who come to the clinic. These organizations foster accessibility rather than continuity. Also, only 64% of users affiliated with professional contact organizations identify a physician who is responsible for their care, which is relatively low compared with the other models (unpublished data). Services are delivered by medical teams of varying sizes that often share space with medical specialists or other professionals. Caregiving teams are composed of physicians and nurses. Technical platforms are available on site to support clinical activities. These organizations are under private professional governance and the method of remuneration is fee for service. In these settings, teamwork is underdeveloped (fairly informal), and few formal links are created with other care providers. Walk-in visits is the predominant method of care delivery, and the range of medical services offered is relatively limited. The nurses' role is reduced to assist doctors in their clinical activities and to manage triage, prevention, and screening activities.

The *professional coordination model *accounts for 22% of the organizations (Table 2). Like the professional single-provider model, followup of regular patients remains a prime concern for these organizations. They foster continuity rather than accessibility. About 80% of users affiliated with this model identify a physician who is responsible for their care (unpublished data). Medical teams are small or medium sized (2 to 6 physicians) and most of them do not include a nurse. This organizational model fosters teamwork for care delivery. PHC professionals share space with medical specialists or other types of health professionals. Private professional governance and fee-for-service payment are core characteristics of this model. As is the case for previous models, professional coordination structures are poorly developed, and few formal links are established with other care providers. However, these organizations offer a wide range of medical services (prevention, diagnosis, and treatment) complemented by referrals to other care providers.

The *professional integrated coordination model* represents 15% of the organizations (Table 2). This group includes over 90% of the FMG clinics surveyed, and these clinics make up about 35% of the organizations in this category. Typically, population health is an issue for professionals in this model. Organizational priorities focus on both accessibility and continuity of care. This model fosters teamwork for care delivery. Caregiving teams are composed of several physicians (more than six) and nurses. Doctors usually share space with specialists and other health professionals. To support their activities, the teams have access to onsite technical platforms and to information technologies. Like the other professional models, these organizations are under private professional governance and, remuneration is fee for service. A distinctive characteristic of this model is that cohesion among professionals and systemic integration are encouraged. Professional coordination structures are developed and formal links are established with other care providers. Practices associated with this model favor both access to services (first contact) and care management of patients. Thus, visits are both by walk in and appointment, opening hours are extended (evenings and weekends), and several doctors are involved in a healthcare access network. Nurses in these settings are at the core of care delivery. They are assigned expanded and innovative roles in the systematic followup of vulnerable patients and ensuring interorganizational liaison and coordination and participate in clinical decisions. A broad range of services is offered. Among the users affiliated with this model, 76% identify a physician who is responsible for their care (unpublished data). 

Finally, the *community model* represents 12% of all organizations (Table 2). Typically, these organizations focus on the population rather than on the individual and emphasize continuity of care. Caregiving teams are composed of several physicians and nurses who foster teamwork and interdisciplinarity. Other health professionals (nonphysicians) are also involved in these settings. Care teams have access to onsite technical platforms and to information technologies to support their clinical activities. This model differs from the others mainly by its governance. Indeed, these organizations are all integrated into public health and services structures such as CLSC and FMU. Physician remuneration is predominantly timebased. These organizations establish minimal relationship with other care providers. Visit type is variable; mixed for some (by appointment and walk-in) and mostly scheduled appointments for others. The scope of services offered is fairly broad. Like the professional coordination model, it is characterized not only by diversified services, but also by an expanded role of nurse in care delivery. Secondary analyses also demonstrated that patients who attend community organizations are relatively younger and less likely to identify a doctor who is responsible for their care [54]. Only 67% of users affiliated with this model report having a physician who is responsible for their care (unpublished data).

## 5. Discussion

We have identified and described the organizational configurations of PHC services delivery in two regions of the province of Quebec. Our findings indicate that the professional integrated coordination model and the community model are closer to the organizational ideal type promoted by current reforms, characterized by group medical practice, multidisciplinarity, longer opening hours, and integration of services into the health system [19, 21–25]. In addition to providing first contact, regular access to a care provider fosters longitudinality in provider-patient relationships, which is essential to the management of chronic diseases [55]. Group medical practices and interdisciplinarity encourage comprehensive service provision including prevention and management of psychosocial problems. In addition, interorganizational relationships that are characteristic of the integrated coordination model are congruent with improved coordination of care among providers and easier navigation of patients within the care system. Further analyses done on the performance of organizational models have also shown that the integrated coordination model and the community model tend to generate better care experiences for the general population, particularly for people with chronic diseases [46, 54]. We thus conclude that these models have great potential for organizational development that will improve PHC service delivery. In our study, all FMG belong to this category of organizations. Our findings suggest that the growth of FMG in Québec since 2003 has induced organizational changes in the desired direction. It remains to be seen whether FMG that have been created after 2005 follow the same pattern as these early adopters.

The other three models are more remote from the ideal pursued by reforms. The professional single-provider model typically corresponds to the traditional model of family medicine. These solo doctors have restricted hours and few formal links with other care providers. However, further analyses done on performance of PHC models have shown that these organizations perform generally very well with regards to the experience of care, particularly for more vulnerable patients [46, 54]. These results can presumably be explained by improved patient-physician relationship, which is at the core of PHC services [56, 57]. Our results suggest that it is important to preserve this aspect of services that characterizes solo practices when creating larger scale and more complex organizations. Clearly, professional models of coordination and contact do not satisfy all the objectives of PHC services delivery. For these PHC models, significant efforts are needed to ensure that they contribute to improve services to the population. For example, walk-in visits, as offered by the contact model, constitute short-term solutions to the problems of access to PHC currently observed [58]. These practices foster access first contact services, but have difficulty broadening their activities and to cover the wide spectrum of roles expected from PHC services [19].

### 5.1. Usefulness of the Configurational Approach and Methods

**Figure 5:**
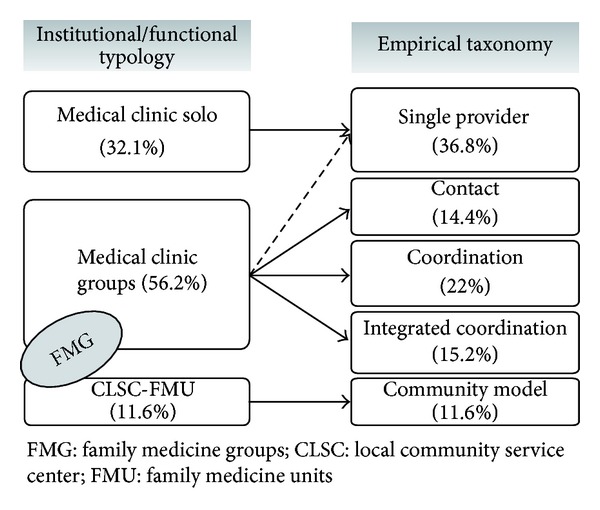
Distribution of PHC organizations, by functional/institutional typology and empirical taxonomy.

When comparing different models of PHC delivery, studies generally use typologies derived from administrative or functional denominations (e.g., CLSC, GMF, and private practices). However, such typologies form heterogeneous classes of organizations, with high intragroup and low intergroup variability. These typologies are then less useful for understanding organizations and comparing them [59, 60]. The taxonomy we developed uses an empirical approach and has generated homogeneous groups of organizations. Organizations are grouped together not only based on the presence or absence of specific attributes, but also on relational combinations that link attributes to each other. Organizational groupings are formed on the basis of internal coherence among organizational characteristics. As shown in Figure 5, the added value is mainly in the distribution of medical group practices into four professional models. Differentiating medical clinics is very useful since, as previously shown, configurations of organizational attributes vary considerably among professional models, particularly in terms of vision and practices. In the context of our study, publicly administered organizations such as CLSC and FMU exhibit more similar organizational forms and are therefore all grouped under the same organizational model. This finding is not surprising; the concept that underpins these two forms of organizations is the same.

Comparing the various organizational models of the taxonomy is very informative. Our study showed that different models have different effects on the care experience of users [46, 54]. For instance, the professional contact model obtained better results than other models for first-contact accessibility. This is not so for other care experience indicators (continuity, comprehensiveness, responsiveness, and outcome of care). Overall, the integrated coordination model performed better on all indicators. Results also showed that the emergence of models is closely linked to their contexts, and the prevalence of these models varies across urban, suburban, and rural settings [46]. In short, the taxonomy provides insight for understanding the meaning of the organizational configurations obtained and their potential as research tools [35].

Finally, studies have convincingly shown that a configurational approach enhances analysis of healthcare organization transformations. Several authors have shown that organizational changes do not result from simply modifying isolated organizational attributes or adding a few structural elements. Rather, in-depth organizational changes come from moving organizational forms toward existing or emerging configurations [61, 62]. If change is to be assessed, evaluations must also provide comparison bases among organizations and over time [35]. The configurational perspective we adopted for this study allowed us to observe how organizational models evolve over time. A study is underway in the same two regions of the province [63]. Its goal is to assess the nature and scope of the organizational changes that have occurred since 2005. The study will enhance our knowledge about the transformation of health services and the way we evaluate them. In the current context of healthcare reorganization, the information generated by these evaluations is very useful for decisions related to health services organization as well as for future studies.

### 5.2. Strengths and Limits

The theoretical foundation of this taxonomy, the conceptually based selection of the variables, and the representativeness of clinical settings are fundamental to the robustness of the results obtained and their external validity [64]. Data were collected from a large number of PHC organizations representing all PHC clinical settings in both regions included in the study. We are thus confident that the validation and operationalization processes of the 43 organizational variables and the factorial analyses allowed us to construct a valid representation of the actual situation regarding PHC organizations. These conditions confer robustness to the results. Moreover, our study represents the situation of a majority of PHC organizations in Québec. The two regions include almost 40% of the province's population and close to half of PHC clinics. These populous regions intersect both urban and rural zones. Nonetheless, some contexts are not represented, such as remote areas and regions far from urban centres. Yet, some studies have suggested that, in these contexts, organizational models differ and bring about different experiences [18, 65]. This is especially true for CLSCs, where studies show that medical practice differs in rural areas. These remarks call for caution when generalizing to other contexts.

Finally, the classification method itself has certain limitations, especially with regards to the stability of organizational groupings. For instance, variable construction and choice of number of factorial axes have an influence on organizations that are farthest from class centres and group membership. In our study, this phenomenon has usually been controlled through an iterative process of class consolidation [48].

## 6. Conclusion

Our study findings contribute significantly to knowledge about PHC and, more specifically, about PHC organizational models in Québec. The configurational approach has proven to be very useful in allowing us to create a valid basis of comparison between organizations. Thus, by reflecting the organizational complexity of PHC services, the taxonomy developed here provides a frame of reference useful to decision makers and practitioners who must bring transformations to healthcare systems. It also serves the purpose of research on organizational change [35, 66]. In our view, the approach used in our studies contributes to organizational sciences by enhancing our understanding of organizational change in health services.

## Supplementary Material

Research report (46) and Organizational questionnaire developed for mail organizational survey of general practitioners' PHC practice settings

## Figures and Tables

**Table 1:** Conceptual domains.

	Conceptual domains	Organizational attributes
Vision	Refers to the mission that states its goals and orientations	(i) Accountability (ii) Purpose services (iii) Values/beliefs/culture
Resources	Quantity and variety of resources to provide services	(i) Human (ii) Financial (iii) Diagnostic and therapeutic technologies (iv) Information technologies
Structure	Provides a regulatory and governance framework for action	(i) Governance (ii) Mode of remuneration (iii) Interprofessional links (iv) Interorganizational links
Practices	Professional and administrative practices embedded in mechanisms that underpin the production and delivery of services	(i) Types of consultation (ii) Delivery/supplies services (iii) Care coordination (iv) Quality of services

Adapted from Lamarche et al. (2003) [42].

**Table 2:** Main organizational attributes by organizational PHC model (percentage of organizations with the modality in the class).

Organizational PHC models (*n* = 473)	Professional models				Community model (*n* = 55)
	Single provider (*n* = 174)	Contact (*n* = 68)	Coordination (*n* = 104)	Coordination integrated (*n* = 72)	
% of organizations	36.8%	14.4%	22.0%	15.2%	11.6%

Vision					

V2 responsibility	Clientele* (83%)	Individuals who present* (44%)	Clientele* (88%)	Population* (26%) or Clientele (64%)	Population* (31%) or clientele (69%)
V3 organizational priority	Continuity > accessibility (84%)	Accessibility > continuity* (54%)	Continuity > accessibility* (89%)	Continuity > accessibility* (76%)	Continuity > accessibility* (95%)
V6 financial return	More important (59%)	More important* (65%)	Less important (52%)	More important* (67%)	Less important* (93%)
V7 team work	Less important* (91%)	More important (50%)	More important* (74%)	More important* (88%)	More important* (75%)

Resources					

R1 size of clinics	Very small* (91%)	Average* (34%) or variable	Small* (57%)	Large* (47%)	Large* (56%)
R3 presence of other professional or specialist	None* (40%)	High* (81%)	High* (69%)	High* (76%)	Average* (56%)
R6 information technologies	Very low* (45%)	Low or very low (70%)	Low or very low (62%)	High* (40%)	High* (49%)
R7 technical platform	Very low* (78%)	High* (32%)	Average* (53%)	High* (24%)	Average* (82%)

Structure					

S1 governance	Professional private* (100%)	Professional private* (100%)	Professional private* (100%)	Professional private* (100%)	Public* (100%)
S5 MD remuneration	Fee for services* (100%)	Fee for services* (100%)	Fee for services* (100%)	Fee for services* (100%)	Time based* (100%)
S7 coordination of care (intraorganizational)	None* (93%)	Informal* (52%)	Informal* (58%)	Formal* (63%)	Formal* (65%)
S8 collaboration with PHC	No (52%)	No* (69%)	No* (61%)	Yes* (88%)	No (53%)
S9 collaborations with secondary care institutions	No (56%)	No* (68%)	No* (66%)	Yes* (86%)	Yes (62%)

Practices					

PZ mode of consultation	Mostly scheduled appointment* (82%)	Mostly walk-in* (69%)	Mostly scheduled appointment (64%)	Mixed (24%) or variable	Mixed (25%) or mostly scheduled appointment (67%)
PB role of the nurses	No nurse* (88%)	Limited* (27%) or no nurse (66%)	No nurse* (75%)	Extended* (55%)	Extended* (82%)
P7 scope of services	Narrow* (59%)	Narrow* (59%)	Broad* (47%)	Very broad* (47%)	Very broad* (69%)
PH quality assessment	None* (100%)	More or less* (68%)	More or less* (66%)	More* (50%)	More* (67%)

*P ≤ .05.
