# Supplementary material for: Constructing Taxonomies to Identify Distinctive Forms of Primary Healthcare Organizations
Source: ISRN Family Med. 2013 Apr 15;2013:798347. doi: 10.5402/2013/798347 (PMC4041222; doi:10.5402/2013/798347)
Supplement: Supplementary file 1 — Research report (46) and Organizational questionnaire developed for mail organizational survey of general practitioners' PHC practice settings [file 798347.f1.zip › Supplementary Material/Add_file_1_Research_report_R-visedMarch2012_ISRNFM_477958.pdf]

ACCESSIBILITY AND CONTINUITY OF CARE: A STUDY OF PRIMARY HEALTHCARE IN QUÉBEC

RESEARCH REPORT

PRESENTED TO THE CANADIAN INSTITUTES OF HEALTH RESEARCH (CIHR) AND THE CANADIAN HEALTH SERVICES RESEARCH FOUNDATION (CHSRF)

Québec

ACCESSIBILITY AND CONTINUITY OF CARE:
A STUDY OF PRIMARY HEALTHCARE IN QUÉBEC

RESEARCH REPORT

PRESENTED TO THE CANADIAN INSTITUTES OF HEALTH RESEARCH (CIHR)
AND THE CANADIAN HEALTH SERVICES RESEARCH FOUNDATION (CHSRF)

POPULATION HEALTH AND HEALTH SERVICES TEAM

PREVENTION IN CLINICAL SETTINGS SECTOR
DIRECTION DE SANTÉ PUBLIQUE
AGENCE DE LA SANTÉ ET DES SERVICES SOCIAUX DE MONTRÉAL

DIRECTION SYSTÈMES DE SOINS ET POLITIQUES PUBLIQUES
INSTITUT NATIONAL DE SANTÉ PUBLIQUE DU QUÉBEC

AND

CENTRE DE RECHERCHE DE L'HÔPITAL CHARLES LEMOYNE

MARCH 2009

Centre de recherche
Hôpital Charles LeMoyne
Centre affilié universitaire
et régional de la Montérégie

Québec
• Agence de la santé et des services sociaux de Montréal
• Institut national de santé publique

WRITTEN BY
Raynald Pineault[1,2,3]
Jean-Frédéric Levesque[1,2,3]
Danièle Roberge[4,5]
Marjolaine Hamel[1,2]
Paul Lamarche[6]
Jeannie Haggerty[4,5]

Statistical analyses
Costas Kapetanakis

Production and publishing support
Audrey Lozier-Sergerie[1]
Mireille Paradis[1]

Cartography
Emanuelle Huberdeau[1]

Translation
Sylvie Gauthier[1]

Principal investigators
Raynald Pineault[1,2,3]
Jean-Frédéric Levesque[1,2,3]
Danièle Roberge[4,5]

Co-investigators
Marjolaine Hamel[1,2], Paul Lamarche[6], Jeannie Haggerty[4,5], Pierre Tousignant[1,2], Léo R. Poirier[2], James Hanley[7], Mike Bénigéri[8], Pierre Bergeron[2], Ginette Beaulne[1,2] and Marie-France Raynault[9]

Advisory committee
Yvon Brunelle[10], Sylvie Cantin[11], Louis Côté[8], Serge Dulude[8], Johanne Fournier[11], François Goulet[12], Richard Lessard[1], Laurent Marcoux[11], Jean Rodrigue[11], Denis A. Roy[11] and Lysette Trahan[10]

1 Direction de santé publique de l'Agence de la santé et des services sociaux de Montréal
2 Institut national de santé publique du Québec
3 Centre de recherche du Centre hospitalier de l'Université de Montréal
4 Centre de recherche de l'Hôpital Charles LeMoyne
5 Université de Sherbrooke
6 Faculté de médecine – Groupe de recherche interdisciplinaire en santé (GRIS)
7 Department of Epidemiology and Biostatistics, McGill University
8 Agence de la santé et des services sociaux de Montréal
9 Département de médecine sociale et préventive, Université de Montréal
10 Ministère de la Santé et des Services sociaux du Québec
11 Agence de la santé et des services sociaux de la Montérégie
12 Collège des médecins du Québec

Funding agencies and partners

This study has received funding from the Canadian Institutes of Health Research (CIHR), the Canadian Health Services Research Foundation (CHSRF) and the Fonds de recherche en santé du Québec (FRSQ). It was also funded by the Agence de la santé et des services sociaux (ASSS) de Montréal and Agence de la santé et des services sociaux de la Montérégie, Institut national de santé publique du Québec (INSPQ), Groupe de recherche sur l'équité d'accès et l'organisation des services de santé de 1re ligne (GRÉAS 1), and Groupe interuniversitaire de recherche sur les urgences (GIRU). The Collège des médecins du Québec, Groupe de recherche interdisciplinaire en santé (GRIS), Observatoire Montréalais des inégalités sociales de la santé (OMISS) and Ministère de la Santé et des Services sociaux du Québec (MSSS) also supported the project.

The project has received ethical approval from the research ethics committee of the Agence de la santé et des services sociaux de Montréal and Hôpital Charles LeMoyne.

This document can be downloaded from the Web sites of the Direction de santé publique (www.santepub-mtl.qc.ca/ESPSS/production.html), INSPQ (www.inspq.qc.ca/publications/) and Centre de recherche de l'Hôpital Charles LeMoyne (www.hclm.qc.ca/fr/centrerecherche/).

This document is printed on recycled paper containing 30% post-consumer waste fibre and processed without elemental chlorine.

Reproductions for the purpose of private study or research are authorised under section 29 of the Copyright Act. Any other use requires authorisation from the Government of Québec and the Centre de recherche de l'Hôpital Charles LeMoyne, which hold exclusive intellectual property rights over this document. Authorisation may be obtained by submitting a request to the central clearing house, Service de la gestion des droits d'auteur des Publications du Québec; the form can be obtained from the following Web site: http://www.droitauteur.gouv.qc.ca/autorisation.php, or by sending an e-mail to: droit.auteur@cspq.gouv.qc.ca.

Data in this document can be cited on condition that the source is credited.

LEGAL DEPOSIT – 1st TRIMESTER 2009
BIBLIOTHÈQUE ET ARCHIVES NATIONALES DU QUÉBEC
LIBRARY AND ARCHIVES CANADA
ISBN: 978-2-550-55435-6 (PRINT VERSION)
ISBN: 978-2-550-55436-3 (PDF)

© Gouvernement du Québec, 2009
Centre de recherche de l'Hôpital Charles LeMoyne (2009)

COLLABORATIONS

The authors wish to acknowledge the support for this study provided by the Direction de santé publique de l'Agence de la santé et des services sociaux de Montréal, the Centre de recherche de l'Hôpital Charles LeMoyne and the Agence de la santé et des services sociaux de la Montérégie.

We would also like to note the quality of the work and professionalism of the numerous individuals who participated in this study, as well as the excellent work of the SOM polling firm, which conducted the population survey.

More specifically, we would like to underline the contribution of Brigitte Simard, Liliane Foisy and Costas Kapetanakis, as well as Roxane Borgès Da Silva, Linda Cazale, Léonarda Da Silva, Danielle Larouche, Adeline Laugraud, Audrey Lozier-Sergerie, Odette Lemoine, Cristine Leroux, Dominique Lesage, Diane Ouellet, Lauriane Robert, Sylvie Provost, Isabelle Rioux and Alexandre Prud'homme, who collaborated on the project. We would also like to acknowledge the participation of Michèle Perron. It was a great pleasure to work with her.

PREFACE

In Canada, a number of provincial and federal committees have recognized problems related to primary care medical services organisation. Over the past 10 years or so, Canadian and Québec health systems have undergone considerable changes that have especially affected primary care services. In Québec, these organisational changes have resulted more particularly in the implementation of Family Medicine Groups (FMG) and network clinics (CR or *cliniques-réseau*) as well as in the creation of health and social services centres (HSSC).

This is the context in which the research project “Accessibility and Continuity of Care: A Study of Primary Healthcare in Québec” was conducted in two densely populated regions of Québec: Montréal and Montérégie. The study looks at organisational models of primary healthcare and their influence on the experience of care of the population (Pineault, Levesque et al., 2004).

The report is intended for healthcare professionals: regional and local decision makers, clinicians, representatives of community groups, as well as anyone interested in having a deeper understanding of the experience of primary healthcare (PHC) of the population. The unpublished data presented in this study provided evidence that informs decision makers, administrators and clinicians about the organisation of primary care services and its influence on the experience of care of users.

The study was conducted by researchers from the Population Health and Health Services team at the Direction de santé publique de l’Agence de la santé et des services sociaux de Montréal and Institut national de santé publique du Québec (INSPQ), as well as researchers from the Centre de recherche de l’Hôpital Charles LeMoyne. Many other Québec researchers collaborated in this study.

KEY MESSAGES

Generally, users of primary care have a favourable opinion of their experience of care. Organisational and geographical accessibility remain the aspect that is the least favourably perceived by the population. We should be concerned with the fact that residents of urban areas in general, and Montréal in particular, have a poorer perception of their healthcare experience.

The adult population is served by primary care medical clinics that have adopted different organisational forms. In the regions studied, we observe five primary care organisational models, four *professional* and one *community. Professional* models—*single-provider, contact, coordination and integrated coordination*—serve 90% of users. These types of organisations are privately governed, and their objective is to respond to the medical needs of who come to clinic, or of people for whom these clinics are the regular source of care. The *community* model includes organisations integrated into public healthcare institutions; their goal is to improve the health of the population in a given region. We should keep in mind the following:

- The *professional single-provider* model, the "solo physician", shows the best performance when it comes to perceived experience of care. This finding reminds us that a personal doctor-patient relationship fosters a better experience of care. And yet, solo practice is fading and being replaced by group clinical practice. It is important to preserve the relational character of this practice when reorganising primary care since reforms will undoubtedly lead to more complex and larger organisations.

- The *professional integrated coordination* model stands above the other models in all aspects of performance and should be considered for implementation of new models of primary care organisation. This model includes, for the large part, "Family Medicine Groups" (FMG) implemented in the two regions at the time of the study. These highly effective organisations are responsible in part for the *integrated coordination* model's high ranking when compared with other organisational models. Consequently, our results support ongoing implementation of FMG which, by inserting themselves into existing organisations, seem to contribute to improving the latter's performance.

- Implementation of "walk-in medical clinics" is not the solution to the current problem of accessibility to primary care. Indeed, organisations that follow the *professional contact* model and prioritise accessibility of services are shown to be the least performing in all aspects of the experience of care, including accessibility. Accessibility is considered more positive by users of the other professional and community models. Finally, these organisations are poorly integrated into the health services network. Therefore, this model does not represent an organisational basis for primary care reform. Specific efforts must be made to ensure this type of organisation contributes to an overall plan designed to enhance the population's access to primary of services.

- It is troublesome to note that the *professional contact* model, which posts the worst performance, includes a significant percentage of FMG and future network clinics (in the accreditation phase in 2005, at the time of the study). Development of emerging types of organisation must be guided by their capacity to improve the performance of primary care.

- Overall, results converge to demonstrate that apart from the *professional contact* model, organisational models are equally equitable when it comes to services rendered to clienteles who are disadvantaged on a socio-economic, educational or health level. Nonetheless, our study suggests that certain socio-economic factors have a negative impact on perception of experience of care and reporting unmet healthcare needs, especially among people who perceive themselves as poor or very sick. However, older people and those who are less educated tend to report better experience of care and fewer unmet needs for care.

Optimal organisation of primary care in a territory or region should take into consideration historical and contextual factors. It can hardly be based only on a single organisational model. We should remember the following:

- Greater availability of primary care and specialized resources at a territorial level is not a guarantee that primary care will perform better, especially in terms of the population's experience of care. Superior performance is associated with organisational models and their integration into the healthcare system. This finding suggests that in addition to favouring certain organisational models, reconfiguration of primary care must seek to create coordinated care networks.

- Models that ensure organisational accountability and patient management, particularly of clienteles with chronic diseases, and that offer a mix of consultation options (e.g., walk-in or by appointment and telephone consultations) seem to be the best way to ensure both accessibility and continuity of care.

- Rather than having isolated FMG organisations, having a certain number of FMG in a territory appears to have greater impact on the population's experience of care, which suggests an implementation strategy that involves saturating rather than scattering FMG.

Organisational and contextual taxonomies developed in this study allow a better understanding of the organisational realities of primary care. They provide a useful frame of reference for decision makers and administrators who wish to characterise delivery of primary care in the territories and follow their evolution over time. In addition, the knowledge generated by this study can be used to anticipate results of changes to the organisation of PHC.

SUMMARY

All around the world, primary care services are undergoing significant reforms. In Canada, a number of provincial and federal committees have alerted us to problems related to primary care medical services organisation. Over the past 10 years or so, Canadian and Québec healthcare systems have undergone considerable changes that have especially affected primary care. In Québec, these organisational changes have resulted more particularly in the implementation of Family Medicine Groups (FMG) and network clinics (CR or *cliniques-réseau*) as well as in the creation of health and social services centres (HSSC).

This is the context in which the research project *Accessibility and Continuity of Care: A Study of Primary Healthcare in Québec* was conducted. The study was conducted in two health regions in the province—Montréal and Montérégie. It looked at organisational models of primary care and their influence on accessibility and use of health services by the population, as well as the experience of users of these services. The main objective of the study was to identify organisational models of primary care that are best adapted and most likely to meet the population's needs and expectations. The research included three components:

1) A survey of the population designed to measure utilisation of health services as well as users' perception of the accessibility, continuity, comprehensiveness, responsiveness and results of services received (Levesque, Pineault et al., 2007a);
2) A study of primary care clinics that aimed to describe and characterise primary care organisation, and to identify the primary care organisation models in the regions studied (Hamel, Pineault et al, 2007);
3) A contextual analysis that sought to describe Health and Social Services Centre (HSSC) territories (Roberge, Pineault et al., 2007).

The study results are of particular interest to healthcare professionals including regional and local decision makers, clinicians, representatives of community groups, as well as anyone interested in having a deeper understanding of the PHC experience of the population who interface with health services organisation. The study also presents a unique opportunity to inform decision makers, administrators and clinicians about the performance of organisations delivering primary care.

Descriptive, methodological reports, thematic reports and articles have already been, or will soon be, published. Interested readers are invited to read these publications to gather all the information generated by this project.

Highlights

- Overall, individuals' assessments of their healthcare experience with their regular source of primary care are favourable. These observations concur with the results of international studies on healthcare user satisfaction. However, the population is clearly less appreciative of geographical and organisational accessibility.
- Assessment of experience of care varies greatly among HSSC territories in the two regions under study. The population's perception of the PHC is generally better in Montérégie than in Montréal.

- The contrasts characterizing the population's experience of care is evident when HSSC territories are grouped by context. Urban territories described as *affluent commerçant* and characterised by the density of their populations and the wide diversity and quantity of healthcare resources obtain the lowest scores for the global index of experience of care as well as for most of the specific indices. These territories are also where the number of people who have regular family physicians is lowest. Rural territories, grouped in the category *équilibré coordonné*, are very different; they obtain the best scores for almost all experience of care indices. Among this population, the percentage of individuals who report having a regular family physician is very high, with an average of 80%.
- Urban territories described as *affluent commerçant* have by far the greatest number of primary care resources. Even when taking into account the fact that resources in urban areas serve a sizeable number of people who live in other territories, this finding suggests that it is primary care than the quantity of resources that shapes a positive experience of care.
- Primary care organisations have been classified into five distinct models: four *professional* and one *community* model. Such a taxonomy is useful to better understand primary care organisation. One advantage resides in the distinction made among privately-owned medical clinics, which are sorted into four different categories.
- The profiles of these models vary according to different aspects of performance, such as response to vulnerable clienteles, coverage of the population, productivity in delivering services, conformity to an ideal-type of organisation, and experience of care of individuals.
- The *professional single-provider* model—characterised by a focus on the client, poor integration into the network, low number of resources, restricted range of services and mostly walk-in consultations—stands out favourably in terms of certain performance aspects, particularly experience of care of users of services and response to the needs of vulnerable clienteles. However, this model shows little potential for population coverage, and poor conformity to an ideal-type of primary care organisation. Although it is unrealistic for this model to represent a viable option for reform, its performance in terms of response to the needs and expectations of individuals highlights the necessity to preserve positive patient-physician relationships in the other, more complex organisational models.
- The *professional contact* model—characterised by a responsibility that focuses on individual clients, moderate integration into the health system, modest number of resources, restricted range of services and mostly walk-in consultations—is the least performing of models on all aspects; it is noticeably unfavourable when compared with other models of primary care organisation. Moreover, contrary to set objectives, service accessibility in this model is inferior to that in other models.
- The *professional coordination* model—characterised by responsibility that focuses on individual clients, moderate integration into components of the health system, average number of resources, a moderate range of services, and services provided mostly on an appointment basis—stands out favourably in terms of productivity, experience of care and population coverage. However, this model's organisational features conform less with an ideal-type of organisation.
- The *professional integrated coordination* model—characterised by population-based responsibility, strong integration into health network activities, significant number of resources, broad range of services delivered, and mixture of services available through walk-in clinics or by appointment—stands out favourably from the other models in all aspects of performance. This model includes, for the large part, "Family Medicine Groups" (FMG) implemented in the two regions at the time of the study. Results concur to demonstrate that organisations associated with FMG show the best performance.

- The *community* model–characterised by a population-based approach, public governance, many resources, broad scope of services offered, and mixture of walk-in or by appointment clinics—stands out in terms of how it conforms to ideal-type of primary care organisations and individuals' experience of care. However, low productivity and limited population coverage reduce its overall performance evaluation. These organisations are integrated into public institutions that extend beyond general medical services delivery. Analysis of potential for reforms should take into account their multiple complementary missions.
- The configuration profile of primary care service delivery differs greatly from one context to another. In rural areas, it is characterised by the predominance of professional integrated coordination organisations and the absence of a *professional contact* model. The *community* model is poorly represented here. In urban contexts, the single-provider model is still predominant, although each model is represented.

Based on study results, we can suggest that the room for improvement in primary care certainly exists. In the current context of rising healthcare costs, the issue of performance is of capital interest to decision makers. Therefore, the preferred organisational models of primary care should be effective as well as equitable for the population. Moreover, given that our results tend to show that contexts interact differently with some of the organisational models, reforms should be based on organisational realities that differ according to contexts. Changes must rest on configuration profiles proper to each territory and user characteristics.

TABLE OF CONTENTS

INTRODUCTION **1**
1 BACKGROUND AND RESEARCH QUESTIONS **3**
2 APPROACHES AND METHODS **7**
2.1 Conceptual framework **7**
2.2 Methods **9**
2.2.1 Populational component 9
2.2.2 Organisational component 10
2.2.3 Contextual component 11
2.2.4 Analysis of organisational performance 12
2.3 Dissemination of results **12**
3 PRIMARY CARE ORGANISATIONAL MODELS **15**
3.1 Description of the organisational models **15**
3.2 Performance of organisational models **18**
3.2.1 The population's experience of primary healthcare 19
3.2.2 Coverage of the population 20
3.2.3 Response to vulnerable clienteles 21
3.2.4 Productivity 23
3.2.5 Organisational potential 24
3.2.6 Synthesis of performance results 25
4 RESULTS PRESENTED BY CONTEXT **27**
4.1 HSSC Territories: Four groupings **27**
4.2 Primary care resources and contexts **29**
4.3 Experience of PHC among the population and in the various contexts **30**
5 DISCUSSION AND CONCLUSION **33**
REFERENCES AND BIBLIOGRAPHY **37**

APPENDIX A – GLOSSARY **43**
APPENDIX B – METHODOLOGICAL ASPECTS **49**
APPENDIX C – PRESENTATIONS AND PUBLICATIONS **77**

List of Tables

Table 1: Synthetic indices of experience of care ... 10
Table 2: Presentations and publications ... 13
Table 3: Characterisation of organisational models of primary care (n=473) ... 15
Table 4: Productivity indicators, by organisational model ... 23
Table 5: Taxonomy of HSSC territories ... 27
Table 6: Primary care resources by context ... 29
Table 7: Primary care organisational models by context ... 30
Table 8: Population's affiliation to organisational models: percentage of users by context (place of residence) ... 31

List of Figures

Figure 1: Reference periods of the 3 components of the study in relation to the reform of the healthcare system ... 5
Figure 2: Conceptual framework ... 7
Figure 3: Study design ... 9
Figure 4: Construction of the organisational taxonomy of primary care ... 11
Figure 5: Experience of care indices by organisational model ... 19
Figure 6: Population coverage: proportion of users and organisational models ... 21
Figure 7: Proportion of vulnerable clientele, by organisational model ... 22
Figure 8: Coverage of vulnerable clienteles: proportion of users who are "highly vulnerable" and organisational models ... 23
Figure 9: Ideal-type conformity score, by organisational model ... 24
Figure 10: Ranking of organisational models, by performance ... 25
Figure 11: Geographical location of territorial groupings ... 28
Figure 12: Experience of care indices by place of residence ... 30

LIST OF TABLES APPENDED

Table B1: Composition of indices of experience of care 52
Table B2: Taxonomy of organisations – Organisational variables 58
Table B3: Description of organisational groupings..... 62
Table B4: Variables retained for the analysis 70
Table B5: Distance of HSSC territories from the centre of their class..... 73
Table B6: Composition of ideal-type conformity scores 74

LIST OF FIGURES APPENDED

Figure B1: Classification tree of organisations 61
Figure B2: Variation of the inertia quotient based on number of classes..... 61
Figure B3: Groupings of organisations into five classes: intra-class variance (IV) and inter-class distances..... 62
Figure B4: Inter-class and intra-class inertia..... 71
Figure B5 : "Inertia quotient increase between partitions"..... 72
Figure B6: Graphic representation of the 4 groups over the first two factorial axes..... 72

INTRODUCTION

This research report presents the results of the project entitled *Accessibility and Continuity of Care: A Study of Primary Healthcare in Québec.* It is the end product of a project involving extensive population-based and organisational data collection and analysis, from the perspective of primary care organisation and performance.

The initial project was more modest, and focused on the region of Montréal. The obvious interest demonstrated by decision makers in Montréal and the Montérégie resulted in sampling in both regions, which ensured population representativity from HSSC territories and completeness of regular sources of primary care. The broader project laid the groundwork for a unique partnership involving research and decision makers from these settings. The partnership guided knowledge exchange activities carried out during this study.

Descriptive and methodological reports, thematic reports and articles have already been, or will soon be, published. Interested readers are invited to read these publications to get a better grasp of the information generated by this project. For purposes of this report, we have retained results that respond more directly to the study's central question: What are the organisational models of primary care that are best adapted and most promising to meet the needs of the population, in particular of socioeconomically disadvantaged individuals?

The first part of the report presents the study background, research questions, and usefulness of the results for decision makers. The second section briefly outlines the approaches and methods used for data collection and analysis. It also describes the strategies chosen to disseminate the results. The results follow in the next three sections. The first of these sections describes results from an organisational perspective, showing the organisational models that emerged from the analyses and how these models performed. Next comes a "territorial perspective" of the results, which presents, as a whole and by context, the population's perceptions of services used and the organisational configuration of services offered. The third section discusses the results in light of the changes brought to primary care, as well as the study limits. The methods used are described in the Appendices.

1 BACKGROUND AND RESEARCH QUESTIONS

An environment of reform

In recent years in Québec and Canada, the health system has been the object of profound re-examination. Significant problems in primary care have been identified in the way services are planned, organised, funded and provided. Fragmentation of services (Kirby, 2002; Clair, 2000), ineffective use of healthcare providers, poor information sharing, collection and management (Kirby, 2002), lack of coordination (Clair, 2000), lack of emphasis on health promotion (Kirby, 2002), as well as barriers to access (Kirby, 2002; Romanow, 2002; Clair, 2002) have been identified as the main problems. These deficiencies are manifest in the accessibility and continuity of services provided and in a reduced capacity to respond to the health needs of the population. There is widespread agreement on the need to reform primary care (Romanow, 2002; Kirby, 2002; Clair, 2000; Sinclair, 1999; Fyke, 2002; Mazankowsky, 2001). Moreover, while the problems observed in the health system extend beyond primary care, a number of experts concur that effective organisation of primary care is essential to solving problems affecting the system as a whole (Starfield, 1998; Clair, 2000; Romanow, 2002; Sinclair, 1999).

Consequently, it is clear that health systems and primary care are under a great deal of pressure to better meet the needs of the population through multiple, often complex care services that can improve performance. It is in this context that Québec's health system is undergoing major changes. A number of these changes—implementation of new organisational models such as Family Medicine Groups (FMG) or network clinics (NC)—directly affect primary care. Others, such as the creation of health and social services centres (HSSC) and local health and social services networks (LHSSN), transform the environment surrounding primary care. All these changes respond to a desire to structure care delivery more coherently.

Nonetheless knowledge about primary care organisation—especially that of private clinics—and the experience of care of individuals who use them are relatively limited (Levesque, Roberge and Pineault, 2007).

Study objectives

This is the context in which the research project *Accessibility and Continuity of Care: A Study of Primary Healthcare in Québec* was conducted in two health regions of Québec: Montréal and Montérégie.

The main objective was to document the various organisational forms that prevail in primary care and to identify the organisational models that are best suited and most likely to meet the needs of the population and, more specifically, those of disadvantaged individuals. In particular, the objectives were as follows:

1) To identify existing primary care organisational models in the regions under study and their distinctive characteristics concerning management, accessibility and continuity, coordination with other providers and integration.

2) Determine the influence of organisational models on accessibility of primary care and unmet needs for care of the population, as well as the experience of care of people who use primary care services;
3) Explore the influence of contexts on prevalence of primary care organisational models and on experience of care of the population.

Usefulness for decision making

At a time when all provincial health systems in Canada are implementing new forms of primary care organisation, this study is important to identify the best ways to respond to the healthcare needs of the population. The report provides evidence on the organisation and performance of primary care, as well as on the experience of care of populations who interface with these services. Under current circumstances, at a time when Québec's health system is undergoing significant changes, these data provide very useful information for decision makers at different levels. Indeed, current reforms incite clinical settings to reposition their practices, and decision makers and administrators to revise ways of ensuring adequate coverage of the population’s healthcare needs and to adopt consequent health policies.

Moreover, this research project coincided with the initial implementation phases of new primary care organisational models and of Health and Social Services Centres (HSSC). Figure 1 illustrates the reference periods for each component of the study and corresponding timeline of reforms affecting primary care organisation in Québec. Notably, study data were collected during the years when FMG were first implemented and preceding the implementation of the first network clinics in Montréal and Montérégie. This conjuncture presented a unique opportunity to influence the ongoing reform, with new evidence emerging throughout the research process. Finally, in this context, the study provides an organisational profile of primary care and of its effects on the experience of care of the population at the beginning of the reform process, thus forming the basis for future evaluation of the transformations.

Figure 1: Reference periods of the 3 components of the study in relation to the reform of the healthcare system

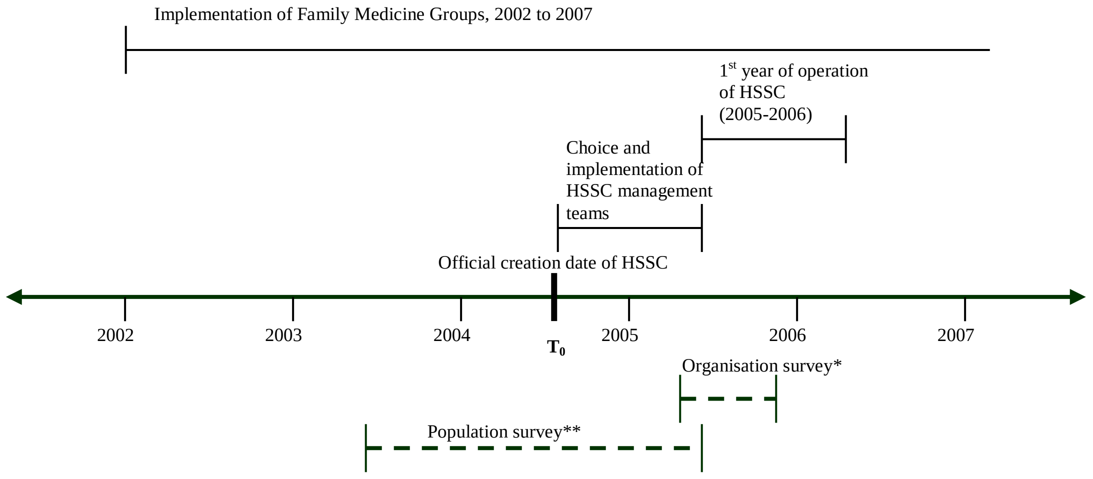

* May to November 2005
** Service utilisation, February 2003 to June 2005

2 Approaches and methods

2.1 Conceptual framework

This study focuses on PHC services offered to the population by general practitioners working in private or public clinics. We conceptualize the care process and its attributes—access/utilisation, unmet needs for care and individuals' experience of care (accessibility, responsiveness, comprehensiveness and perceived outcomes of care)—as the result of the interface between populations and the organisation of services serving the public, in specific contexts (Figure 2).

Figure 2: Conceptual framework

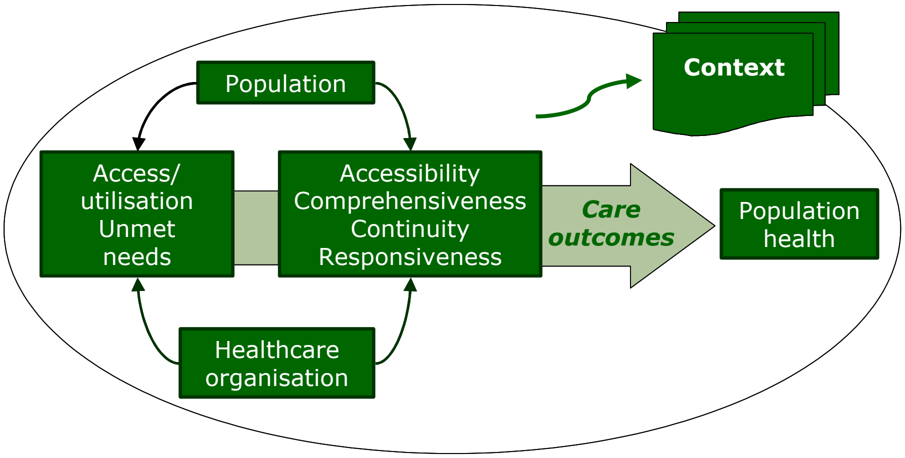

Service organisations

The definition of "organisation" used in this study refers to organisational entities that include one or several general practitioners offering general medical services. Therefore private, single-doctor offices are regarded as "organisations". Offices and clinics with more than one physician are also considered "organisations" when physicians share a minimum number of administrative resources (rooms, secretarial services or archives), whether or not their professional practices are integrated. Service points tied to a CLSC (local community services centre) but located at different sites and having their own medical equipment are considered to be different organisations.

Service organisations are conceptualized as systems for organised action (Contandriopopulos, Denis et al., 2001). A configurational approach was chosen to study the organisations. "We use the term organisational configuration to denote any multidimensional constellation of conceptually distinct characteristics that commonly occur together." (Meyer, Tsui and Hinings, 1993). Like Lamarche, Beaulieu et al. (2003), we understand that organisations result from particular configurations of four components:

- Vision: beliefs, representations, values and objectives that allow persons involved to communicate and legitimate their actions
- Resources: the quantity and variety of resources available

- Organisational structure: rules, regulations and other agreements that frame and guide partners' actions, their reciprocal relationships and governance
- Practices: mechanisms that underlie the production of activities and services.

Experience of PHC

While notions of access and utilisation are prerequisites to the experience of care, the concept of **unmet needs for care** describes a situation where a person perceives or feels a need to obtain health services but doesn't. **Accessibility** refers to the ease with which individuals can use the services; it includes geographical, organisational, economic and cultural barriers to use (Levesque, Pineault et al., 2007a). **Continuity** describes the phenomenon of fluidity and breaks in temporal sequence during which several services must be provided. Services are continuous if care is connected and coherent (Haggerty, Burge et al., 2008; Reid, Haggerty et al., 2002). **Responsiveness** is a concept put forward by the World Health Organization (Murray, Frenk, 2000) which refers to the responsiveness of system to the legitimate expectations of the population concerning elements or actions not related to the technical content of treatment. There are two main components of **responsiveness**: respect for persons and attention to the patient (Levesque, Pineault et al., 2007a). **Comprehensiveness** measures an organisation's response to a variety of healthcare needs (Starfield, 1998). **Outcomes of care** describe the effects or consequences of health services on individuals (Levesque, Pineault et al., 2007a) (see definitions in Appendix A).

Contexts

Similar to the study of primary care organisations, we chose a configurational approach to characterise the contexts in which the actors work (Meyer, Tsui, Hinings, 1993; Miller, 1992, 1996). The multidimensional character of these contexts were considered (Cannon, St. John, 2007; Scott, Mendel, Polack, 2004). The aspects considered are the following: health needs of the population, availability of resources on the territory, and degree of clinical collaboration among primary care organisations and between these organisations and hospitals.

Organisational performance

In the current context of rising healthcare costs, the issue of performance is of capital interest to decision makers. As a result, we decided to broaden our initial framework by adopting a general framework for evaluating the performance of primary care organisation models. We have integrated questions about the association between organisational models and experience of care, as well as the concerns we have for socioeconomically disadvantaged individuals, into the conceptualisation developed to assess organisational performance.

As suggested by Champagne, Contandriopoulos et al. (2005), we used a comparative performance model to compare different types of organisations to each other. The criteria for comparison is essentially empirical and relative, that is, based on current performances of the organisations we compared. Indeed, we too consider performance as a generic concept encompassing notions of effectiveness, efficiency, productivity, quality and other indicators used to evaluate organisations. For purposes of this study, we identified the following organisational dimensions of performance: experience of care, coverage of the needs that reflects the relative importance of vulnerable clienteles, population coverage, productivity as a measure of economic performance, and organisational potential.

2.2 METHODS

The research focused on four broad areas: a populational component, an organisational component, a contextual component, and an analysis of the performance of organisations. The first three components involved data collection and were each the subject of methodological and descriptive reports (Levesque, Pineault et al, 2007a; Hamel, Pineault et al., 2007; Roberge, Pineault et al., 2007). As shown in Figure 3, the study design included a nominal link between results of the populational and organisational components. This distinctive feature meant that very high rates of response to the surveys had to be achieved.

Figure 3: Study design

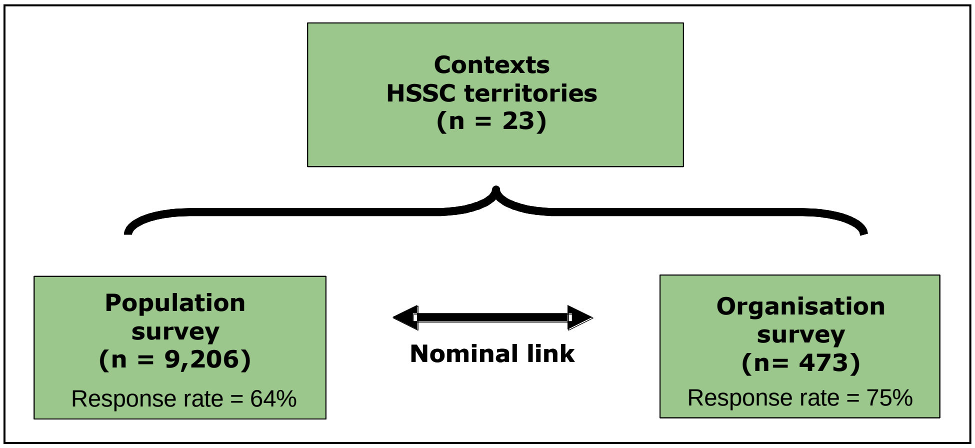

2.2.1 Populational component

A telephone survey of individuals aged 18 and over was conducted in the regions of Montréal and Montérégie. The survey was carried out in 2005 by a polling firm; it reached over 9,200 people. It allowed us to document the adult population's use of regular source of primary care and users' perceptions of the accessibility, continuity, comprehensiveness and responsiveness of primary care services, as well as the perceived outcomes of care. Participation in the survey was very good, with response rates of 63% in Montréal and 66% in Montérégie (overall rate: 65%). The sample design chosen enabled us to produce a series of indicators disaggregated by HSSC territory, and thus assess the parameters of primary care service utilisation, including unmet needs for care and experience of care.

The report entitled *L'expérience de soins de la population : portrait des variations intra-régionales à Montréal et en Montérégie* (Levesque, Pineault et al., 2007a) presents the methodology, detailed results and highlights. The indicators discussed in this report show that overall, individuals' assessments of their healthcare experience with regular source of primary care are favourable. These observations are similar to the results of international studies on healthcare user satisfaction (Schoen, Osborn et al., 2004). However, appreciation of experience of care varies greatly among territories of the HSSC in the two regions under study.

To measure these variations, an aggregate index of experience of care and seven specific indices were constructed, based on the answers to 29 items from the questionnaire. Factorial analyses compiled items

based on the seven aspects that characterise a experience of care: geographical and organisational accessibility, economic accessibility, continuity of affiliation and follow-up, informational continuity, comprehensiveness, responsiveness and perceived outcomes of care (Table 1). For comparative purposes, the variables that make up each of the indices were dichotomised and the score calculated by adding variables from each index. Scores were converted into percentages, taking into account the number of variables that made up the index. The method used to construct experience of care indices is presented in Appendix B1.

Table 1: Synthetic indices of experience of care

Dimensions of the experience of care	Number of items	Cronbach's alpha
**Global index of experience of care**	29	.87
**Geographical and organisational accessibility**<br>E.g.: Distance and opening hours	5	.45
**Economic accessibility**<br>Ex: Fees incurred for uninsured services	3	.42
**Continuity of affiliation**<br>E.g.: With a regular source of care	4	.70
**Informational continuity**<br>E.g.: Follow-up after a test or visit to a specialist	3	.57
**Responsiveness**<br>E.g.: Non-clinical aspect of care; respect and human dignity	5	.70
**Comprehensiveness**<br>E.g.: Comprehensive/biopsychosocial approach to care	4	.78
**Perceived outcomes of care**<br>E.g.: Control of the condition; acquisition of healthy lifestyle habits	5	.84

2.2.2 Organisational component

A postal survey of general practitioners' offices and PHC clinics was undertaken in the regions of Montréal and Montérégie (n=665)[1]. The survey, conducted in 2005, focused on the visions of organisations, material, financial and human resources, current organisational structures, and organisational practices that support service delivery. The research team put much effort into the survey, which resulted in very high participation rates. A total of 473 organisations participated in the study, for a response rate of 71% (66% in Montréal and 81% in Montérégie). The various types of private and public primary care organisations were well represented (solo, group, CLSC, family medicine units, and FMG).

The report entitled « *L'organisation des services de santé de première ligne : Portrait des services médicaux de première ligne à Montréal et en Montérégie* » (Hamel, Pineault et al., 2007) presents the

[1] Clinics studied included private offices (solo or group), FMG, family medicine units (FMU), and physician groups in local community services centres (CLSC). One key informant per organisation was chosen to answer the self-administered questionnaire. Most of the time, the respondent was a physician in charge of the organisation or the one identified as the best person to answer regarding the clinic's overall activities.

methodology, detailed results and highlights of the survey. Results converge to demonstrate that primary care resources vary greatly among HSSC administrative regions and territories. These differences can be seen in the availability and types of resources in the territories and in the organisational characteristics of medical clinics.

We adopted a taxonomic approach to study organisational forms. The strategy enabled us to consider a significant number of organisational attributes that describe the services. We retained 43 organisational variables that captured the characteristics proper to the organisations' visions (9 variables), structures in place (10 variables), resources they have (7 variables), and practices that ensure service delivery (17 variables). The analyses performed using these data enabled us to classify organisations into five homogeneous, well-defined categories (Figure 4). The SPAD software (Version 6) was used for the analyses. Appendix B2 shows the variables, analyses performed and statistical indicators that support our decisions concerning the solutions chosen.

Figure 4: Construction of the organisational taxonomy of primary care

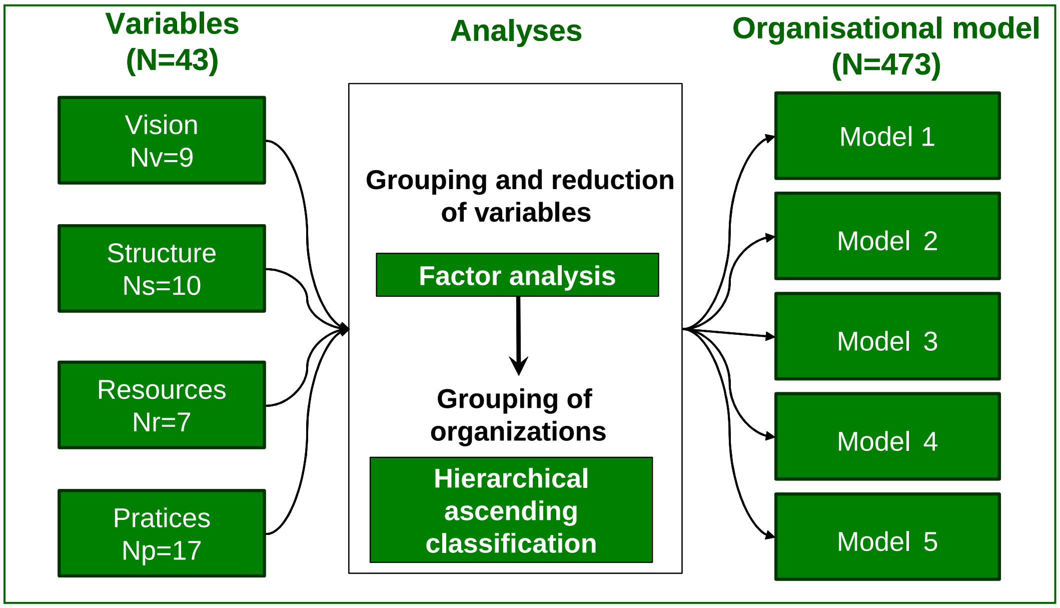

2.2.3 Contextual component

The contexts explored (n=23) are the geographical territories of the new local governance structures recently implemented in Québec: Health and Social Services Centres (HSSC). Similar to the study of primary care organisations, we chose a configurational approach to characterise the HSSC territories (Meyer, Tsui and Hinings, 1993; Miller, 1992, 1996). The result is a taxonomy composed of a limited number of groups looking to maximise intragroup homogeneity and heterogeneity among groups. For more information on the taxonomy construction process, readers can refer to the methodological report of the context analysis (Roberge, Pineault et al., 2007).

The taxonomy was constructed to ensure the contexts' multidimensionality were taken into account (Cannon and St. John, 2007; Scott, Mendel and Pollack, 2004). The dimensions and variables chosen to characterise the HSSC territories are presented in Appendix B3. Their relevance and usefulness for management purposes were validated with managers. The dimensions include the **health needs of the population** (6 variables), **availability of resources** on the territory (4 variables) and **degree of clinical collaboration** (2 variables) among primary care organisations and between these organisations and hospitals.

Overall, the variables used to characterise the contexts were documented, based on usual sources of administrative data. A telephone survey of chief executive officers in each HSSC was conducted to document the level of clinical collaboration in their territories. Taxonomy construction is predominantly based on two exploratory techniques: a multiple correspondence analysis (factorial analysis) and ascending hierarchical classification. The SPAD software (Version 6) was used for the analyses. Appendix B3 shows the variables, analyses performed and statistical indicators which were used as a basis for decisions regarding the final classification.

2.2.4 Analysis of organisational performance

We integrated, into the conceptualisation we developed to assess organisational performance, the main study questions about the association between organisational models and experience of care, as well as our concerns for socioeconomically disadvantaged individuals. For purposes of this study, we operationalised the following dimensions of performance: (1) experience of care measured with the eight synthetic indices of care presented in Section 2.2.1; (2) population coverage as a percentage of all users served by a given group of organisations; (3) response to vulnerable clienteles measured with five indicators (health status, level of education, economic situation, poverty and age) and an index of high vulnerability; (4) organisational productivity measured by number of visits by FTE physician, number of patients by FTE physician, percentage of clinics that set aside 30 minutes or less for new case evaluations, and percentage of clinics that reserve less than 15 minutes for follow-up and emergency consultations; and (5) organisational potential, measured by a conformity score to an organisational ideal-type. We looked to the literature on primary care organisation to identify the characteristics associated with better functioning of primary care (Brunelle, 2006; Gouvernement du Québec, 2003, 2006; Starfield, 1998) (Appendix B4).

For these analyses, information from the organisational and population-based components were nominally linked. Bi- and multi-variate statistical analyses (multiple regressions) were performed to study the relationship between organisational models and experience of care indices, and to evaluate the associated factors. Other analyses looked at factors associated with having a regular family physician, a condition that fosters good experience of care, and occurrence of unmet needs for care (Appendix B5).

2.3 DISSEMINATION OF RESULTS

Knowledge exchange occupied a very important place throughout this project. It should be noted that, for the last few years, different local, regional and national decision making bodies have been grappling with reform of primary care organisation. Many questions accompany this agenda, especially with regard to perception of the population, services offered and performance of primary care organisations.

Ongoing information sharing mechanisms with different groups, such as the steering committees of the Agences de la santé et des services sociaux de Montréal and Montérégie, and HSSC directors' and MSSS administrators' tables, fostered constant dialogue during the project. Discussions were also facilitated by the involvement of decision makers sitting on the advisory committee. The research team's many presentations attest to the efforts made (Table 2). Decision makers and think tanks requested a number of these meetings. For example, two presentations were given to the GETOS group (Gouverne et transformation des organisations de santé, sponsored by CHSRF and CIHR). Finally, the richness of these exchanges should be mentioned, even though such knowledge exchange strategies are very demanding for the research team, which has to remain flexible and available.

Two descriptive reports (one on results of the population-based survey and the other on the results of the organisational survey), a methodological report on analyses of the contexts, thematic reports, summaries and scientific articles were produced and then distributed to decision-making bodies and research groups (see documents attached to the report). Additional efforts were made to translate the summaries and reports into English and to publish these versions on the Web sites of the INSPQ, Direction de santé publique de l'Agence de la santé et des services sociaux de Montréal and the Centre de recherche de l'hôpital Charles LeMoyne. Finally, dissemination strategies also included participating in many scientific conferences and writing articles. The list of presentations and publications pertaining to this research project is included in Appendix C.

Finally, the project was the subject of an article in a medical journal. In addition, two daily newspapers, *La Presse* and *Journal de Montréal* conducted interviews about the project results and published articles in February 2008.

Table 2: Presentations and publications

Type of activities	Local and regional	Provin-cial	National	Interna-tional	Total
Presentations to partners/ decision makers	24	7	-	-	31
Invited presentations	2	10	2	2	16
Unsolicited communications	-	-	8	14	22
Reports, summaries and thematic reports	14				
Book chapters	2				
Scientific articles	4				
Articles for the general public	3				
**Total**	**92**				

3 PRIMARY CARE ORGANISATIONAL MODELS

3.1 DESCRIPTION OF THE ORGANISATIONAL MODELS

The taxonomy of organisations obtained in this study enabled us to collect a great deal of information and, as a result, to document thoroughly each emerging organisational model. Moreover, it is consistent with our previous work on primary care organisations (Lamarche, Beaulieu et al., 2003; Lamarche et Pineault, 2006; Haggerty, Pineault, Beaulieu, 2004). Following the same logic, there are four professional organisational models. The objective of these organisations is to respond to the medical needs of patients who come in to obtain these services or of people for whom these clinics are the regular source of care. The population does not play a role in the administration or funding of these organisations. These types of organisations account for 88% of clinical settings in our study. There is also a community model. Organisations of this type are integrated into public healthcare network institutions. Their goal is to improve the health of the population in a given region. The population can be involved in their administration. About 12% of participating clinics belong to this category.

Each model is characterised based on the vision of the actors, composition of organisational governance, internal integration (interprofessional links), external integration (interorganisational links), quantity and type of resources, and organisational practices. A summary of this information is presented in Table 3.

Table 3: Characterisation of organisational models of primary care (n=473)

ASPECTS		PROFESSIONAL MODELS				COMMUNITY MODEL
		Single-provider 37%	Contact 14%	Coordination 22%	Integrated coordination 15%	12%
**Vision**	**Accountability**	Clientele	Individuals who come to the clinic	Clientele	Clientele - Population	Population - Clientele
**Structure**	**Governance**	Private - Professional				Public
	**Internal–External integration**	Low - low	Medium - low	Medium - low	High - high	High - medium
**Resources**	**Quantity and variety**	Small	Medium	Medium	Large	Large
**Practices**	**By appointment – Walk-in**	Mostly by appointment	Mostly walk-in	Mostly by appointment	Mixed	Mixed
	**Range of services**	Restricted	Restricted	Average	Wide	Wide

Professional single-provider model

This organisational model includes 37% of PHC organisations (Table 3). Among users of primary care, about 11% identified an organisation from this group as their regular source of care[2]. It comprises the offices of general practitioners who work essentially on their own. It is clearly the simplest organisational structure. The typical *professional single-provider* model has the following characteristics[3]:

- Private professional governance and fee-for-service payment;
- A vision based on the principles of family medicine, with organisational priorities being continuity of services and follow-up of regular clienteles.
- Most often, one physician per organisation, no on-site nurse or technical support centre. Occasionally, two or three physicians share the space but their practices remain separate and little integrated;
- Little information technology to support clinical activities;
- Fairly limited services offered: mostly visits by appointment; reduced coverage time (weekdays only); restricted range of services; few formal links with other care providers.

Professional contact model

This model includes 14% of organisations (Table 3). Among users of primary care, about 23% identified an organisation from this group as their regular source of care. This model stands out because of the vision upon which organisation of service delivery is based. For these organisations, the emphasis is on provision of primary care medical services. The typical *professional contact* model has the following characteristics[3]:

- Private professional governance and fee-for-service payment;
- Organisational priorities that converge toward accessibility of services and responding to the short-term medical needs of people who come to the clinic;
- Medical teams of varying sizes, occupying space in a building where other specialists are located.
- More or less formalised professional group work, and lack of interdisciplinarity (with nurses);
- Mostly walk-in visits; restricted range of services and few formal links with other care providers.

2 n = 5,636; data weighted for age, sex, composition of the household and place of residence.
3 To be included in a specific model, an organisation must have several specific characteristics, but not necessarily all the characteristics (details about the methodology can be found in Appendix B).

Professional coordination model

This model includes 22% of organisations (Table 3). Among users of primary care, about 23% identified an organisation from this group as their regular source of care. The typical *professional coordination* model has the following characteristics[3]:

- Private professional governance and fee-for-service payment;
- Organisational priorities that converge towards continuity of services and follow-up of regular clients;
- Small- and medium-sized medical teams (two to six doctors), and no nurse;
- More or less formalised professional group work, and lack of interdisciplinarity (with nurses);
- Mostly walk-in visits; an average range of services complemented by referral networks but few formal links with other care providers.

Professional integrated coordination model

This model includes 15% of organisations (Table 3). Among users of primary care, about 32% identified an organisation from this group as their regular source of care. Of the four *professional* models, this one is distinguished by an organisational structure that fosters greater cohesion among clinic professionals as well as greater systemic integration. This group includes over 90% of organisations with FMG-affiliated physicians (main and affiliate sites)[4]. Family Medicine Groups (FMG) make up about 35% of the organisations under this model.

A typical *professional integrated coordination* model has the following characteristics[3]:

- Private professional governance and fee-for-service payment;
- A vision based on the principles of family medicine, with organisational priorities being continuity of services and follow-up of regular clients; accountability for the health of the population is also involved;
- Teams of caregivers consisting of several physicians (more than four) and nurses;
- Cohabitation with specialists and other health professionals;
- Formalised professional group work, and developed interdisciplinarity (work with nurses);
- Broad range of care modalities: A mix of consultation options (walk-in and by appointment); greater coverage time (evenings and weekends); a broader range of services supplemented with formal inter-organisational links with other care providers.

[4] At the time of the study, in 2005, 30 FMG (principal + affiliate sites) had been implemented in Montréal and Montérégie. Over 90% of these organisations are in the integrated coordination model. Twenty-two FMG (principal + affiliated sites) were in the process of being accredited. These FMG organisations are mostly distributed among the professional integrated coordinated (32%), coordination (32%) and contact (27%) models. Moreover, 30 network clinics were also involved in the accreditation process, 40% of which are in the integrated coordination model and 40% in the contact model.

Community model

This model includes 12 % of organisations (Table 3). Among users of primary care, about 11% identified an organisation from this group as their regular source of care. Organisations that follow this model are all public organisations such as CLSC and FMG. The typical *community* model includes the following:

- public governance and fee-for service and time-based compensation;
- A vision that focuses on accountability for the health of the population which gives priority to continuity of care;
- Teams of caregivers consisting of several physicians (more than 6) and nurses;
- Presence of other health professionals (non physicians);
- Formalised professional group work and interdisciplinarity that has been developed (work with nurses);
- A mix of consultation options (walk-in and by appointment); a broader range of services.

In short

In Montréal and Montérégie, primary care services are grouped into five organisational models: one community model and four professional models. The models differ based on the vision under which services are organised, the complexity of their structure, their capacity to integrate into the health system, and the organisational practices.

3.2 PERFORMANCE OF ORGANISATIONAL MODELS

The issue of performance is currently of great interest to decision makers. For this reason, we have adopted a comprehensive framework for assessing the performance of organisational models that integrate questions about the association between organisational models and experience of care, as well as the concerns we have for socioeconomically disadvantaged individuals. Results are presented according to the five following dimensions:

- *The population's experience of care*
- *Coverage of the population*
- *Response to vulnerable clienteles*
- *Productivity*
- *Organisational potential*

3.2.1 The population's experience of primary healthcare

The *experience of care* of primary care users was assessed using an aggregate index of experience of care and seven specific indices relating to geographical and organisational accessibility, economic accessibility, continuity of affiliation and follow-up, information continuity, comprehensiveness, responsiveness, and perceived outcomes of care. Reported unmet needs for care is also included under this heading. Results of the analyses show the following:

- Overall, individuals' assessments of their healthcare experience with their regular sources of primary care are favourable. However, the population is clearly less appreciative of geographical and organisational accessibility. On average, less than 70% of the questions retained to describe this dimension were given a positive assessment by users.
- The aggregate index of experience of care shows that perception of PHC is generally more positive among users of the *professional single-provider* model (Figure 5). On average, 80% of the questions retained to describe experience of care were assessed very positively by these users. Organisations that follow this model also stand out for each specific index of experience of care; they had the best scores, even when controlling for users' personal conditions. Organisations that follow the *coordination* and *integrated coordination* models were highest, after solo organisations, for all indices except geographical, organisational and economic accessibility, and information continuity; *community* models scored higher on these indices. The latter organisations stood out only in the area of economic accessibility, where they obtained the best score. They were second for geographical and organisational accessibility, and information continuity. Finally, the *professional contact* model ranked last in all dimensions of the experience of care.
- Reports of unmet needs for care are significantly lower among users of *single-provider* (14%) and higher among users of *contact* (22%) model organisations. Rates for the three other models are around 18%.

Figure 5: Experience of care indices by organisational model

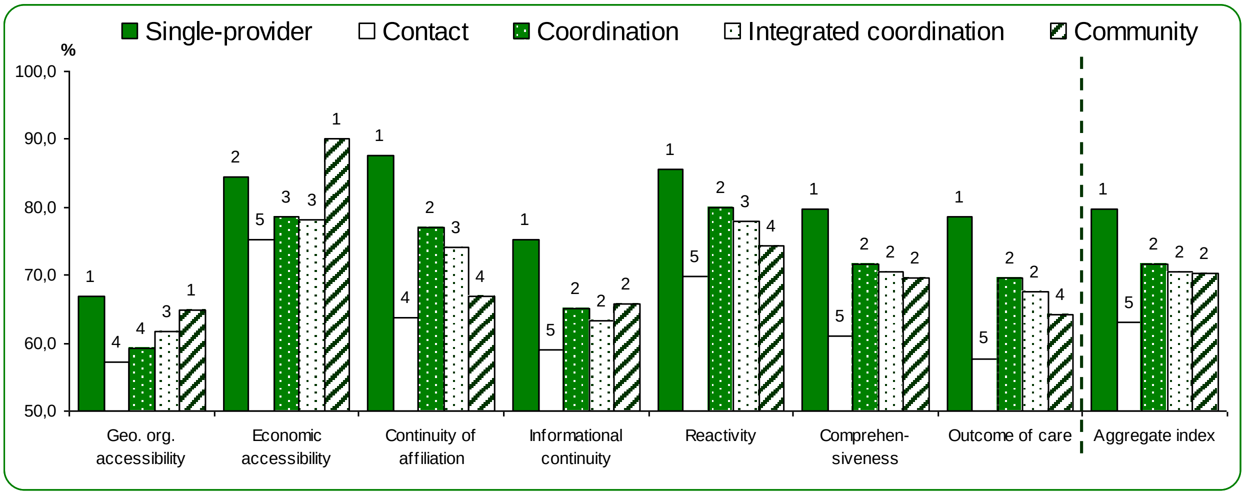

The differences among the models are statistically significant. The greatest contrast is between *professional single-provider* models, which perform best, and the *professional contact* model, the lowest performing one. Marked differences between these models persist even when controlling for individual user characteristics (multiple regression analyses). The other models are positioned between these two extremes for almost all dimensions of the experience of care with the exception of economic accessibility, where the *community* model performs best.

Moreover, results show that a greater proportion of users (94%) affiliated with *single-provider* clinics have regular family physicians. The figure is 82% for those affiliated with *coordination* and *integrated coordination* models, and 72% for users affiliated with *community* model organisations. Finally, the figure for individuals affiliated with *contact* model organisations is only 69%; this model posts the worst performance regarding experience of care. Results of the analyses (multiple regression analyses) demonstrate that having a doctor is a determining factor in individuals' perceptions of experience of care.

3.2.2 Coverage of the population

With regards to the impact of primary care services delivery on the population, we observe that professional model organisations serve close to 90% of users; consequently they assume the largest part of services in terms of population coverage (Figure 6). Organisations that have adopted the integrated coordination model serve the highest proportion of users (32%), even though they make up only 17% of organisations. Conversely, professional single-provider organisations only reach 11% of users but account for 31% of organisations[5].

The potential of organisational models regarding population coverage is seen in the ratio of number of users per model to number of organisations per model (u/o ratio), as presented in Figure 6. The professional integrated coordination model posts the highest u/o ratio (1.93), which indicates that more users are reached for a similar number of organisations. These results suggest that development of this model would certainly help improve population coverage by investing in fewer organisations compared with other models.

5 Percentages do not coincide exactly with those presented in Table 3. Data in the latter Table were calculated based on all organisations participating in the study (n=473) while data in Figure 6 were calculated for organisations that could be linked to a user or users (n=416).

Figure 6: Population coverage: proportion of users and organisational models[6]

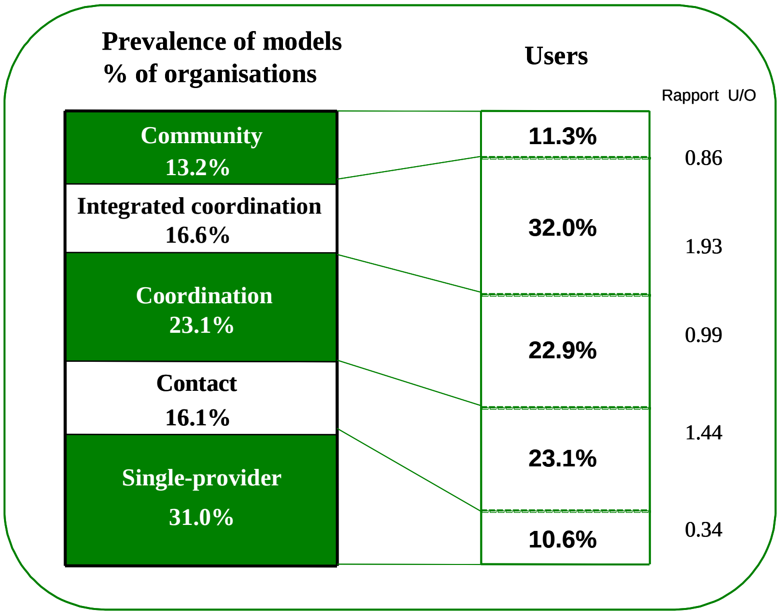

3.2.3 Response to vulnerable clienteles

The impact of primary care services delivery was assessed for vulnerable clienteles. This aspect of performance refers to the burden that greater patient vulnerability can represent for an organisation. The five following conditions were considered: (1) health condition: serious; (2) level of education: low; (3) financial situation: insufficient income; (4) poverty: perception of being poor or very poor; (5) age: ≥ 65 years. An aggregate index of "high vulnerability" was constructed based on individuals' accumulation of conditions of vulnerability (3 conditions and over).

Overall, we observe that groups of older individuals have better perceptions of their experience of care and have fewer unmet needs for care compared with the population as a whole. The same goes for groups of people who are less educated. People who are vulnerable with respect to health tend to report having a less positive experience of care and more unmet needs for care. The same applies to people who perceive themselves as being poor. We note that clienteles who describe themselves as "highly vulnerable", that is, those with three or more conditions of vulnerability, usually have a better perception of experience of care and have fewer unmet needs for care (data not shown). These aspects of vulnerability will be the subject of future analyses.

6 Percentages of organisations presented in Figure 6 do not coincide exactly with those in Table 3. Data in the Table were calculated based on all organisations participating in the study (n=473) while data in Figure 6 were calculated for organisations that could be linked to a user or users (n=416).

Figure 7 illustrates the percentage of vulnerable clienteles by organisational model. The models' ranking is based on performance regarding response to vulnerable clienteles. We observe that the clientele of *single-provider* organisations include a larger proportion of older, poorer and less educated individuals. It is interesting to note that the *community* model clientele is composed of a high proportion of people who are socioeconomically disadvantaged, and the *integrated coordination* model includes a high percentage of less educated individuals. Finally, *coordination* and *contact* models rate last on these aspects of performance.

Figure 7: Proportion of vulnerable clientele, by organisational model

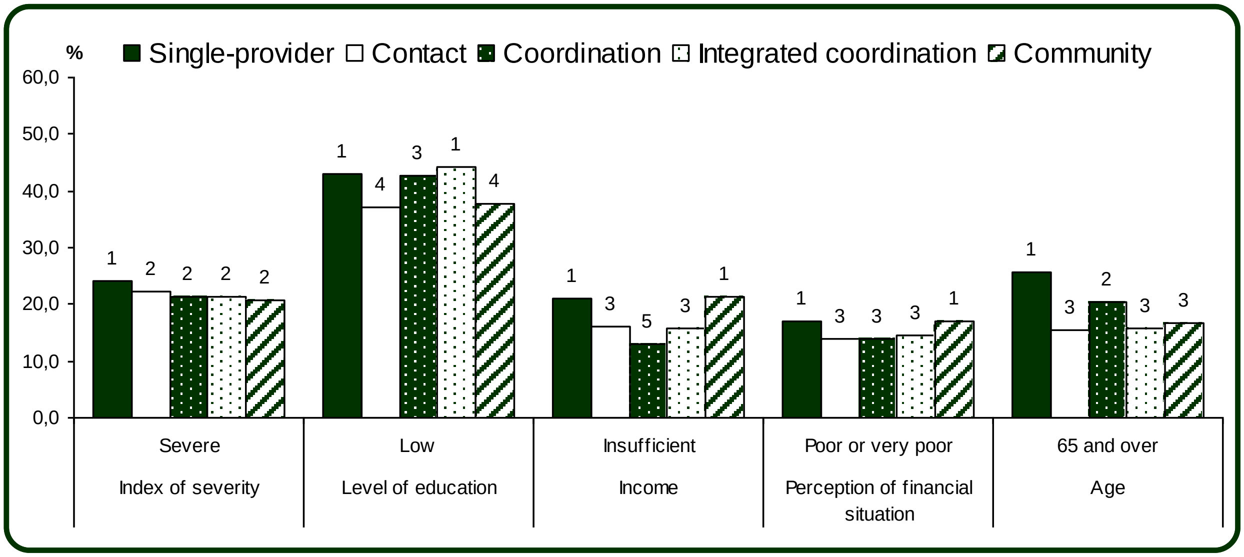

Finally, we observe that integrated coordination models are the organisations that reach the highest percentage of users who are "highly vulnerable" (33% of "highly vulnerable" clienteles) (Figure 8). When we compare these data with those in Figure 6, which illustrate the population coverage of different models, we note that *single-provider* model organisations see relatively more people who are "highly vulnerable" (15% vs. 11%). The inverse is true for the *contact* model (19% vs. 23%).

Figure 8: Coverage of vulnerable clienteles: proportion of users who are "highly vulnerable" and organisational models[7]

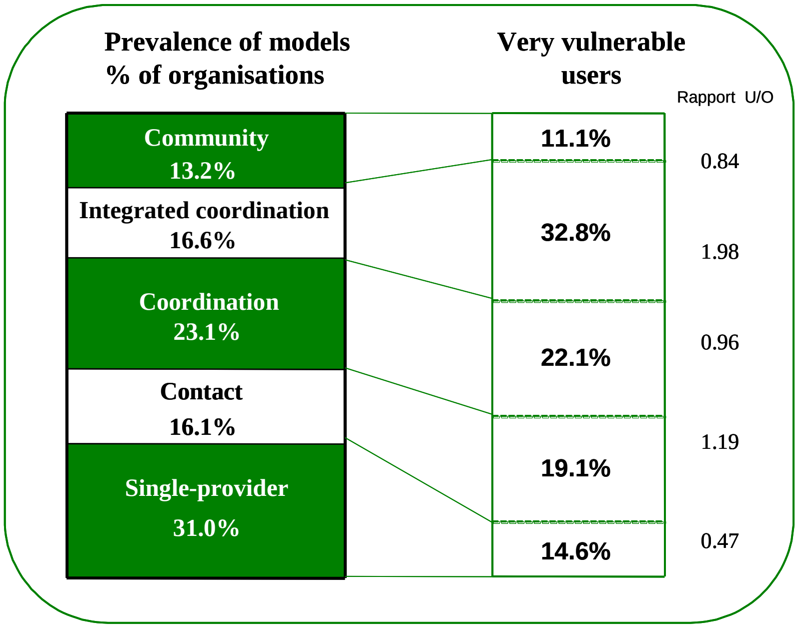

3.2.4 Productivity

Organisational productivity was assessed using four indicators: (1) index calculated based on number of visits by FTE physician; (2) index calculated based on number of patients by FTE physician; (3) percentage of clinics that set aside 30 minutes or less for new case evaluations; and (4) percentage of clinics that reserve less than 15 minutes for follow-up and emergency consultations (Table 4).

Table 4: Productivity indicators, by organisational model

	Professional models				Community model
	Single-provider	Contact	Coordination	Integrated coordination	
Number of visits index	3.5	2.8	3.2	2.9	1.0
Number of patients index	3.2	2.6	2.9	2.6	1.0
Visit time for complete examination ≤ 30 min. (% of organisations)	73.6	89.6	87.5	91.3	49.1
Visit time for follow-up or emergency ≤ 15 min. (% of organisations)	69.8	88.1	88.5	87.0	25.5

7 Percentages of organisations presented in Figure 8 do not coincide exactly with those in Table 3. Data in the Table were calculated based on all organisations participating in the study (n=473) while data in Figure 8 were calculated for organisations that could be linked to a user or users (n=416).

The first two indices reveal that organisations which have adopted *professional* models seem to be the most productive. These results are consistent with average time of visits, which tends to be longer in *community* model than in *professional* model organisations.

However, these data should be interpreted with caution. Indeed, once past a certain threshold, productivity can be associated with a drop in quality. Our data do not allow us to qualify the interpretation of these indicators. In addition, findings related to the *community* model can be somewhat underestimated. It is important to remember that our evaluation targets general medical services and does not take into account medical consultations conducted in the institutions' other programmes, such as CLSC (corresponding to the *community* model). This situation might have caused a few measurement biases.

3.2.5 Organisational potential

Organisational potential was measured using organisational ideal-type conformity scores (Appendix B4). The chosen indicators refer to the following organisational dimensions: vision, structure, resources, and practices. Results for organisational conformity are expressed in percentages (Figure 9). The more ideal-type attributes an organisation has, the more performing it is considered to be.

Overall, professional integrated coordination and community models obtain better results than the other models. Single-provider models have the weakest results, especially concerning “structure” and “resources”. These results are not surprising since by definition, the organisations are characterised by their structural simplicity and by the presence of physicians working in solo.

Figure 9: Ideal-type conformity score, by organisational model

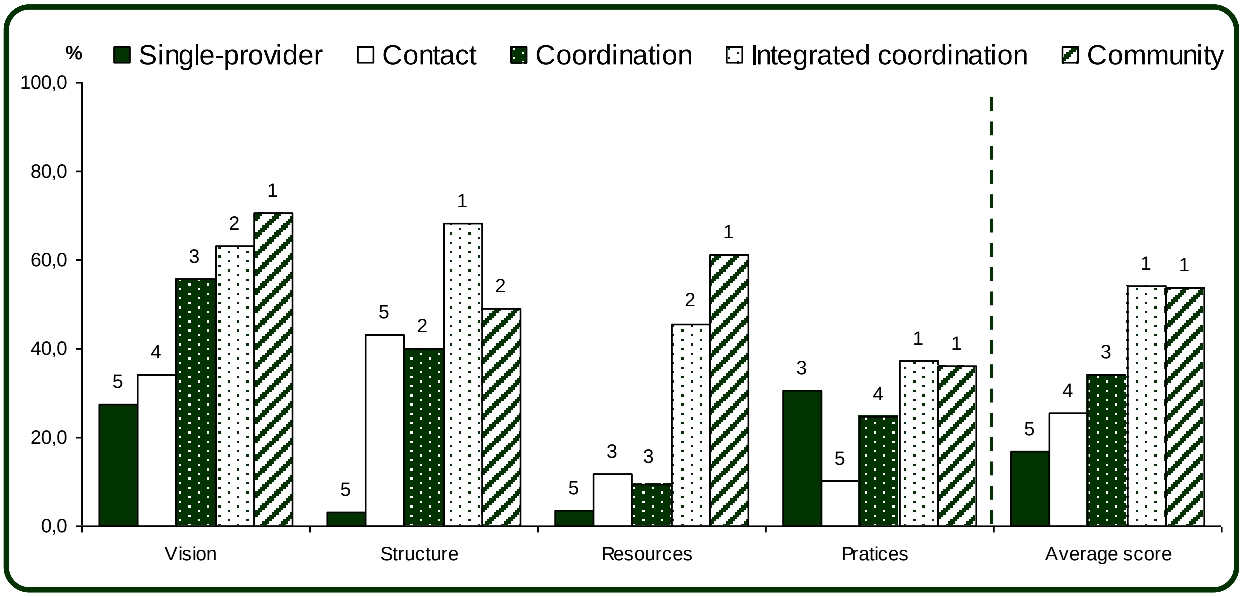

3.2.6 Synthesis of performance results

Figure 10 presents a synthesis of results for different aspects of performance of primary care organisational models, by relative ranking in relation to each other.

Figure 10: Ranking of organisational models, by performance

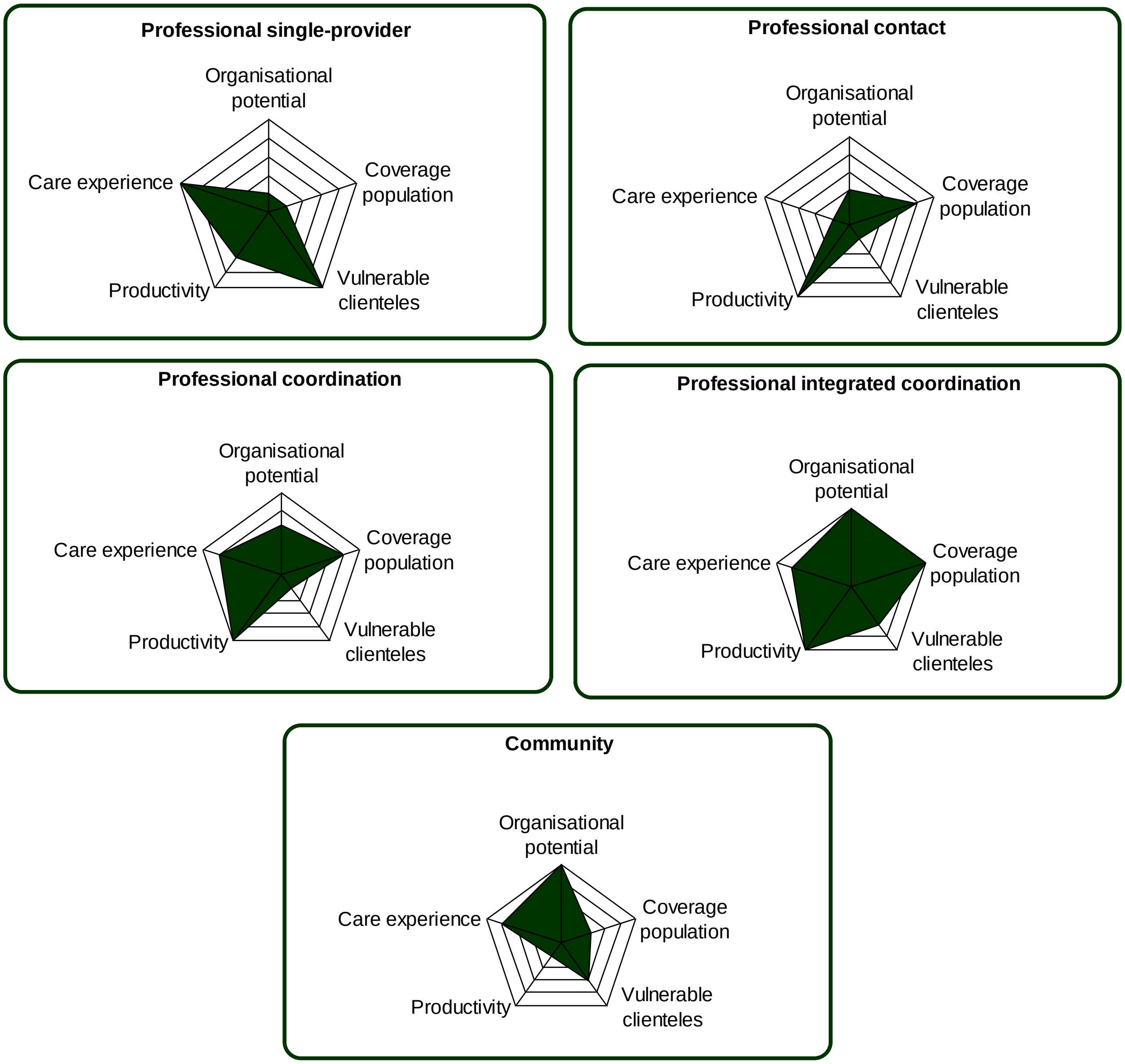

In short

The *professional integrated coordination* model clearly distinguishes itself from other types of organisations on all aspects of performance, while the *professional contact* model ranks last. The *professional coordination* and *single-provider* models, as well as the *community* model post variable scores, depending on various aspects of performance.

As regards the rather weak overall performance of the community model, we should remember that our evaluation did not look at the CLSC's whole mission but rather only at routine medical care. Compared with the other models, having other missions may have influenced productivity findings. We should also note that response to the needs of vulnerable clienteles and the organisational potential of public institutions are high and achieve the same levels as the *professional integrated coordination* model.

The *professional single-provider* model retains our attention; it ranks first in several aspects of performance (experience of care, response to vulnerable clienteles and productivity). The good performance regarding experience of care is dependent on the patient-physician relationship, which is at the heart of primary care provision (Safran, Tarlov and Rogers, 1994; Safran, Kosinski et al., 1998). Consequently, our results suggest that it is important to preserve this aspect of services that characterises solo physicians when creating bigger, more complex organisations.

4 RESULTS PRESENTED BY CONTEXT

In this section, results are presented from a “territorial” perspective. HSSC territories, which are in effect administrative districts, have been grouped together so as to link territories similar in terms of population, resources and inter-organisational collaboration. The results presented in this manner provide a systemic view that can be used to assess the perception of a territory's population regarding services used and to depict the configuration of PHC organisations.

4.1 HSSC TERRITORIES: FOUR GROUPINGS

The territories explored (n=23) are the geographical territories of new local governance structures recently implemented in Québec: Health and Social Services Centres (HSSC). The 23 HSSC territories in Montréal and Montérégie were considered according to the health needs of the population, availability of resources on the territory, and degree of clinical collaboration among primary care organizations and between these organizations and hospitals.

HSSC territories were divided into four distinct groups. The following Table describes each group based on the gap between their populations’ needs and available resources, spatial organisation of primary care services (import and export of primary care[8]) and the extent of clinical collaboration within the territories.

Table 5: Taxonomy of HSSC territories

**Dimensions**	**Urban central 4 HSSC**	**Urban peripheral 7 HSSC**	**Suburban 8 HSSC**	**Rural 4 HSSC**
	Affluent commerçant	***Dépourvu dépendant***	***Pourvu Indépendant***	***Équilibré coordonné***
Health needs	low	high	low	average
Availability of resources	high	low	high	average
**Resources/needs gap**	++	--	+	=
Import/export of primary care	open market (import/export)	mixed market (import)	closed market	closed market
Clinical collaborations	low	low	low	high

HSSC territories are “naturally” grouped around a rural/urban axis. The urban central group, described as *affluent commerçant*, is characterised by the high concentration of hospitals with specialised and primary care resources. Compared with other contexts, the quantity of these resources greatly surpasses the needs of the territory’s residents. This group is “*commerçant*” because the territory's resources serve a certain percentage of the clientele that lives in other territories, for the most part, and because residents of these territories do not necessarily choose their territory’s resources (open market). The urban peripheral group,

8 The notion of market refers to the degree to which residents of an HSSC territory use the area’s services or those located in other territories (import) and the degree to which the services available in a territory are used by residents of other territories (export).

described as *dépourvu dépendant*, is characterised by the low number of resources compared with other groups as well as by the needs of the population. It is described as dependent since HSSC import primary care services for their population from other territories. The suburban grouping is described as *pourvu indépendant*. Compared with other groups, this one is considered to have a relatively adequate supply of resources. The number of resources available is above that required to meet the needs of the population. Territories in this group can be perceived as independent since they import and export few primary care services. The needs and resources of the rural grouping, described as *équilibré coordonné*, are relatively balanced; it is the only group where clinical collaboration among territorial resources is high. The following map shows the geographical locations of HSSC territorial groupings.

Figure 11: Geographical location of territorial groupings

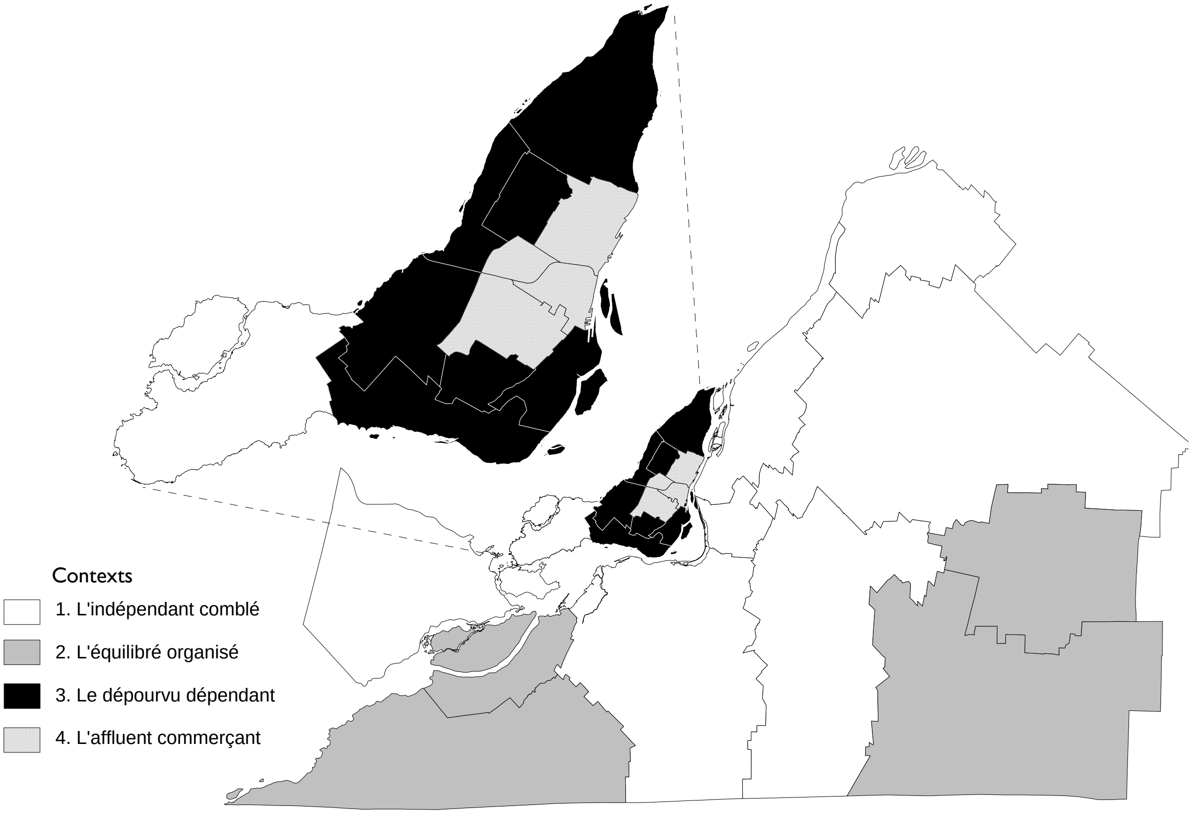

Urban central territories are located in the heart of the island of Montreal. Urban peripheral territories constitute Montréal’s urban belt. Suburban territories are located in Montérégie, and include an HSSC territory located to the west of the island of Montréal. Rural territories are all located in Montérégie and are totally or partially rural geographical sectors.

4.2 PRIMARY CARE RESOURCES AND CONTEXTS

Primary care resources vary greatly among HSSC administrative regions and territories. These differences can be seen in the availability and types of resources and in the organisational characteristics of medical clinics. The figures grouped by context reveal significant territorial differences.

Results show that urban central territories described as *affluent commerçant* have by far the greatest number of primary care resources (Table 6). However, this finding is not reflected in the proportion of the population who report having a family physician, since this is the context where rates are lowest. Conversely, the rural context described as *équilibré coordonné* is where the highest proportion of individuals who have family physicians is found. At the time of study (2005), over half of clinics in this context were associated with FMG; in the other contexts, FMG were at best in the early stages of implementation.

Table 6: Primary care resources by context

**Context**	**Urban central (4 HSSC):**	**Urban peripheral (7 HSSC):**	**Suburban (8 HSSC):**	**Rural (4 HSSC):**	**Prevalence**
	Affluent commerçant	***Dépourvu dépendant***	***Pourvu indépendant***	***Équilibré coordonné***	
Number of GP – FTE/100 000 inhabitants*	**129**	**69**	73	80	84
Population with a family physician (%)	**59.9**	66.0	74.0	**83.1**	69.1
Clinics associated with FMG (%)	**2.4**	**2.5**	**4.6**	**56.7**	6.5

Note: Dark = contexts where prevalence for a model is higher than the average prevalence.
Grey = contexts where prevalence for a model is lower than the average prevalence.
* Data taken from the organisational survey (Hamel, Pineault et al., 2007).

It is interesting to note that differences persist among contexts with regards to primary care organisational models (Table 7). *Professional single-provider* and *contact* models are proportionately more common in urban contexts, while *integrated coordination* models are less so. The configuration of organisational models differ in the rural context. Indeed, while *professional contact* model is absent, over half of organisations fit the *integrated coordination* model.

Table 7: Primary care organisational models by context

Context	Urban central (4 HSSC):	Urban peripheral (7 HSSC):	Suburban (8 HSSC):	Rural (4 HSSC):	Prevalence
	Affluent commerçant	*Dépourvu dépendant*	*Pourvu indépendant*	*Équilibré coordonné*	
***Professional single-provider* model (%)**	**42.0**	**39.4**	**32.0**	30.3	36.8
***Professional contact* model (%)**	16.0	16.1	14.5	0.0	14.4
***Professional coordination* model (%)**	18.3	22.6	27.9	3. 0	22. 0
***Professional integrated coordination* model (%)**	12.2	9.5	14.0	**54.5**	15.2
***Community* model (%)**	11.5	12.4	11. 0	12.1	11.6
**Total**	100.0	100.0	100.0	100.0	100.0

Note: Dark = contexts where prevalence for a model is higher than the average prevalence.
Grey = contexts where prevalence for a model is lower than the average prevalence.

4.3 Experience of PHC among the population and in the various contexts

The contrasts characterizing the population's PHC are evident when HSSC territories are grouped by context (Figure 12).

Figure 12: Experience of care indices by place of residence

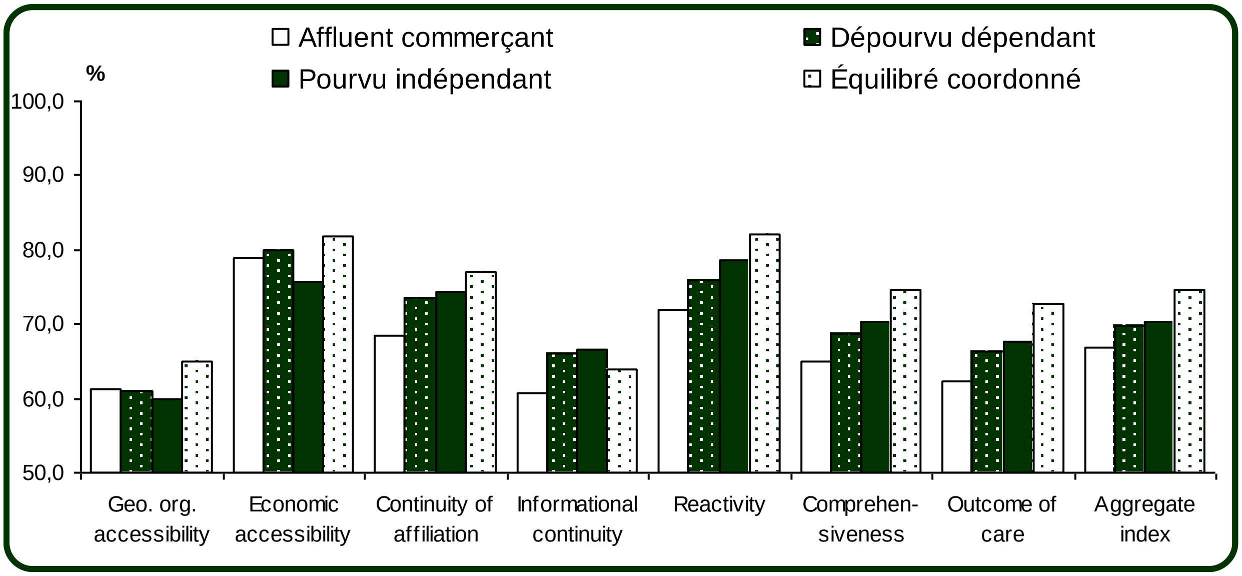

Direction de santé publique, Agence de la santé et des services sociaux de Montréal
Institut national de santé publique du Québec
Centre de recherche de l'Hôpital Charles LeMoyne

As seen in Figure 12, populations residing in urban territories, described as *affluent commerçants*, post the lowest scores for experience of care. On average, fewer than 67% of the questions employed to describe overall experience of care were assessed very positively by these users. The same finding applies for almost all indices. These populations also report the greatest number of unmet healthcare needs (20% versus 18% on average) (Levesque, Pineault et al., 2007b). Results also show that it is in these territories that the highest percentage of people report having a *professional contact* model of organisation as regular source of primary care (Table 8).

Rural territories, grouped in the category *équilibré coordonné*, are very different; they obtain the best scores for almost all experience of care indices (Figure 12). The figure for reported unmet needs for care is also good, amounting to only 15% of the population (vs. 18% on average). Results reveal that most (over 80%) people living in these territories reported having a *professional integrated coordination* model organisation as regular source of primary care (Table 8)

Results for urban peripheral and suburban contexts vary between the two extremes and obtain near average rates of reported unmet needs for care (18%). The organisational affiliations of populations in these territories are divided among different organisational models. In both contexts, the *professional single-provider* and *community* models reach the fewest individuals (Table 8)

Table 8: Population's affiliation to organisational models: percentage of users by context (place of residence)

% of users	Urban central (4 HSSC): *Affluent commerçant*	Urban peripheral (7 HSSC): *Dépourvu dépendant*	Suburban (8 HSSC): *Pourvu indépendant*	Rural (4 HSSC): *Équilibré coordonné*	Prevalence
***Professional single-provider* model (%)**	11.0	13.4	8.8	9.8	10.6
***Professional contact* model (%)**	**28.5**	22.8	25.2	0.9	23.1
***Professional coordination* model (%)**	18.7	24.6	27.3	2.2	22.9
***Professional integrated coordination* model (%)**	25.2	24.8	**30.6**	**80.9**	32.0
***Community* model (%)**	16.6	14.4	8.2	6.2	11.4
**Total (%)**	100.0	100.0	100.0	100.0	100.0

Note: Dark = contexts where prevalence for a model is higher than the average prevalence.
Grey = contexts where prevalence for a model is lower than the average prevalence.

In short

The contexts are marked by significant organisational differences. There are clear contrasts between contexts defined as *affluent commerçant* and *équilibré coordonné.* Our results indicate that, at the population level, quantity of resources–physicians does not ensure better experiences of care. Indeed, the worst PHCs were reported by residents of urban central territories (*affluent commerçant*). Rural territories (*équilibré coordonné*) are distinguished by the predominance of organisational models that result in the best experiences of care, as perceived by the population.

Although a series of factors interact in a certain way to yield differences in experience of care and reported unmet needs of care, the variations measured suggest that there is a potential for improvement that can be further exploited.

5 Discussion and Conclusion

The results of this study show that overall, individuals' assessments of their healthcare experience with their regular source of primary care are favourable. These observations are similar to the results of international studies on healthcare users satisfaction (Schoen, Osborn et al., 2004). However, experience of care assessment varies greatly among HSSC territories in the two regions under study. The population's perception of the PHC in Montérégie is generally better than in Montréal.

Contrasts in the population's PHC are emphasized when HSSC territories are grouped by context. Urban territories, described as *affluent commerçant* and characterised by the high density of their populations and the wide diversity and quantity of healthcare resources, obtain the lowest scores for both global index of experience of care and most specific indices. It is also in this context that the number of people who have a regular source of primary care is lowest (60%). Rural territories, grouped in the category *équilibré coordonné*, are very different; they obtain the best scores for almost all experience of care indices. Having a regular source of primary care is also very high among this population, with an average rate of 80%.

Moreover, results show that urban territories described as *affluent commerçant* have by far the greatest number of primary care resources. Even when taking into account the fact that resources in urban areas serve a sizeable number of people who live in other territories, this finding suggests that it is the organisation of primary care services rather than the quantity of resources that shapes a positive experience of care.

The configuration of PHC organisation differs greatly from one context to another. Indeed, a characteristic of the rural context is the predominance of organisations (55%) that come under the *professional integrated coordination* model, which is centred on clientele follow-up and team work, interdisciplinarity and interorganisational coordination. Conversely, the *contact* model, which focuses on accessibility of services and meeting the short-term medical needs of individuals who visit these clinics, is nonexistent in these territories. In the other contexts, this model accounts for about 15% of organisations and about 25% of users. The *community* model is poorly represented in the rural context, reaching only about 6% of the population versus 17% in urban central territories. Finally, the *single-provider* model remains a prevalent model in urban contexts, even though only 11% of users have chosen this type of organisation.

The *professional integrated coordination* model clearly distinguishes itself from other types of organisations on all aspects of performance, while the *professional contact* model ranks last. These findings deserve to be assessed in the light of primary care transformations currently in progress. In Québec, the implementation of new types of organisations—for instance, FMG and, to a lesser degree, network clinics—are the cornerstone of transformations of primary care. The number of FMG has been growing since 2003. However, almost all FMG are associated with the *professional integrated coordination* model, which is the most effective model. Moreover, when compared with organisations of the same model, FMG stand out and perform better in all respects. These data correspond with the ones we published previously (Pineault, Levesque, et al., 2008). All these results point to the superior performance of FMG when compared with other primary care organisations. Does this effect truly result from the addition of FMG to existing clinics or is it due to a favourable selection of clinics at the onset? Our data does not enable us to come to a conclusion regarding this issue.

We can presume that by integrating FMG, organisations will raise their ideal-type conformity scores and, consequently, their organisational potential. The appeal would be to have an organisation move from a lower to a higher performing model. At the time of study, FMG organisations that were in the process of being accredited were about equally divided among three professional models of the taxonomy, that is, the integrated coordination (32%), coordination (32%) and contact (27%) models. For the most part, the evolving network clinics were classified as *integrated coordination* (40%) and *contact* models (40%). This raises legitimate concerns with the fact that a high proportion of these organisations come under the least performing model, the *contact* model. Further studies and analyses are needed to identify the effects of transformations on network clinics, including their migration towards better performing models.

The *professional single-provider* model retains our attention; it ranks first in several aspects of performance: population experience of care, response to vulnerable clienteles and productivity). Its poor performance relating to organisational aspects and population coverage contrasts with its very good performance on other aspects of organisational performance. Users' positive perceptions of their experiences are largely attributable to the close relationship that develops between patient and physician. However, in the context of primary care reconfiguration, this model tends to be seen as an "endangered species" (Savard and Rodrigue, 2007). When implementing larger, complex organizations and networks, it is important to remember that the health professional/patient relationship is at the heart of primary care provision. Moreover, insofar as these types of organisations are doomed to disappear, we should also be thinking about taking charge of the highly vulnerable clienteles who would lose their doctor.

Scope and limits of the study

Although only two regions of Québec were examined in this study, we are confident that the organisational statements apply to a majority of primary care organisations. Our results are consistent with our previous work (Lamarche, Pineault, 2006; Haggerty, Pineault, Beaulieu, 2004; Lamarche, Beaulieu et al., 2003). Moreover, these populous regions intersect both urban and rural zones. These regions account for almost 40% of the province's population and over half of primary care clinics. On the other hand, not all contexts were represented in our study. Yet, studies suggest that primary care organisational models can produce different experience of cares in different contexts, in particular when comparing areas closer to and further from large urban centres (Haggerty, Pineault, Beaulieu, 2004; Haggerty, Burge et al., 2008). Finally, in regard to CLSCs, studies show that the medical practice developed in these centres differs by region. Such considerations require caution regarding generalisations that can be made based on our data.

A second limit concerns the measures used to express performance and performance comparison. Although various dimensions of performance were considered, the indicators used did not measure all aspects of performance. Moreover, they probably do not all have equal weight in overall organisational performance.

The population survey enabled us to obtain valuable, otherwise unavailable data on the population's use of services and on unmet needs for care. Nonetheless, these data are derived from survey and therefore comprise the limits inherent in using such a research tool. In particular, the population-based survey provided a series of data on user perception as PHC. In addition, the cross-sectional nature of the study design compels us to be cautious in determining relationships among organisational variables (independent variables) and organisational performance variables (dependent variables).

Finally, the taxonomy of contexts that was developed shows that HSSC territories can be grouped around a set of dimensions and that there seems to be a certain coherence among these dimensions. Despite reservations regarding the exhaustive nature of the dimensions under consideration and as regards the exportability of the taxonomy to remote regions, the latter can be a useful tool for regional resource planning by making it possible to conduct ecological studies of primary care organisation.

Conclusion

Based on study results, we can suggest that the room for improvement in primary care certainly exists. In the current context of rising healthcare costs, the issue of performance is of capital interest to decision makers. The preferred models should be effective as well as equitable for the population. Moreover, reforms should be based on organisational realities that differ according to contexts, especially as our results tend to show that contexts can interact differently with some organisational models.

We should underline the need to better define factors likely to improve the experience of care of urban residents in general and, more particularly, of those living in Montréal. In this regard, the contexts in which experience of cares are most favourable should be investigated. Future analyses will aim to clarify the influence on the emergence and performance of organisational models.

REFERENCES AND BIBLIOGRAPHY

Breton M, Lamarche P, Pineault R. Un défi de taille pour les gestionnaires. Le Point en administration de la santé et des services sociaux, juin 2005; vol 1; numéro 2.

Brunelle Y. La pratique de groupe. Direction des services médicaux et médecine universitaire Ministère de la Santé et des Services sociaux. Groupe de travail sur la première ligne. Ref Type: Serial (Book, Monograph), 2006.

Cannon AR, St. John CH. Measuring environmental complexity: A theoretical and empirical assessment. Organizational Research Methods, 2007;10(2):296-321.

Clair M. Les solutions émergeantes. Rapport et recommandations, Commission d'étude sur les services de santé et les services sociaux, Gouvernement du Québec, 2000, 410 pages.

Champagne F. Contandriopoulos A-P, Touché-Picot J, Béland F, Nguyen H. Un cadre d'évaluation globale de la performance des systèmes de santé. Le modèle EGIPSS, 2005.

Contandriopoulos AP, Denis JL et coll. Intégration des soins : dimensions et mise en œuvre. Ruptures, revue transdisciplinaire en santé, 2001, 8(2):38-52.

Donabedian A. Capacity to Produce Services in Relation to Need and Demand. In Aspects of medical Care administration. Cambridge (Mass.), Harvard University Press, 1973:418-485.

Dussault G. L'utilisation des services de santé. Dans les notes du cours d'Analyse des systèmes de santé. Département d'administration de la santé. Université de Montréal. Août 1993, 38 pages.

Feifer C. et al. Different Paths to High-Quality Care: Three Archetypes of Top-Performing Practice Sites. Annals of Family Medicine, 2007;5(1):233-241.

Frenk J. The concept and measurement of accessibility. In Health Services Research: An Anthology. Pan American Health Organization (Editors) Washington, 1992;17:858-864.

Fyke KJ. Caring for Medicare: Sustaining a quality system. Commission on Medicare, Government of Saskatchewan, April, 2001, 82 pages.

Gouvernement du Québec. Projet loi 25 : Loi sur les Agences de développement de réseaux locaux de services de santé et de services sociaux, 2003.

Gouvernement du Québec. Garantir l'accès : un défi d'équité, d'efficience et de qualité, 2006.

Haggerty J, Burge F, Levesque J-F, Gass D, Pineault R, Beaulieu M-D, Santor D. Operational definitions of attributes of PHC to be evaluated: consensus among Canadian experts. Annals of Family Medicine 2008 (sous presse).

Haggerty J, Pineault R, Beaulieu M-D, Brunelle Y, Gauthier J, Goulet F et al. Patients' experiences of primary care in Québec before major reforms. Canadian Family Physician, 2007;53:1057.

Haggerty J, Pineault R, Beaulieu M-D. The impact of the organization of primary care medical services on accessibility and continuity. Rapport de recherche de la FCRSS. Montréal, 2004. Ref Type: Serial (Book, Monograph).

Hamel M, Pineault R, Levesque J-F, Roberge D, Foisy L, Simard B. Méthodologie de l'enquête organisationnelle. L'accessibilité et la continuité des services de santé : Une étude sur la première ligne au Québec. Institut national de santé publique du Québec, Direction de santé publique de l'Agence de la santé et des services sociaux de Montréal, Centre de recherche de l'hôpital Charles LeMoyne (à paraître 2008).

Hamel M, Pineault R, Levesque J-F, Roberge D, Lozier-Sergerie A, Prud'homme A, Simard B. L'organisation des services de santé de première ligne. Portrait des services médicaux de première ligne à Montréal et en Montérégie L'accessibilité et la continuité des services de santé: Une étude sur la première ligne au Québec. Institut national de santé publique du Québec, Direction de santé publique de l'Agence de la santé et des services sociaux de Montréal, Centre de recherche de l'hôpital Charles LeMoyne, rapport et résumé 2007. (Résumé anglais : Primary care services organisation. A portrait of primary care medical services in Montréal and Montérégie).

Kirby M.J.L. The Health of Canadians – The Federal Role. Final Report. Volume Six: Recommendations for Reform, The Standing Senate Committee on Social Affairs, Science and Technology, Chair: The Honourable Michael J.L. Kirby, Deputy Chair: The Honourable Marjory LeBreton, Senate of Canada, 2002, 351 pages.

Lamarche P, Pineault R. Projet 3 du programme de recherche « Gauthier J, Haggerty J, Pineault R, Lamarche P, Morin D, Sylvain H, Levesque J-F, Feldman F, Abbrahamowicz M. Modèles d'organisation des services de santé primaire et accès aux services pour les communautés rurales, éloignées et isolées du Québec ». Programme de recherche financé par la FCRSS, RC2-0985-05, 2002-2006, 2006.

Lamarche P, Beaulieu M-D, Pineault R, Contandriopoulos A-P, Denis J-L, Haggerty J. Sur la voie du changement : Pistes à suivre pour restructurer les services de santé de première ligne au Canada. Fondation canadienne de la recherche sur les services de santé, 2003.

Lebart L, Morineau A, Piron M. Statistique exploratoire multidimensionnelle. 3e éd., Dunod, Paris 2000.

Levesque JF, Pineault R, Kapetanakis C, Hamel M, Roberge D, Robert L, Simard B, Primary care affiliation and un meet needs for healthcare services for vulnerable population-based survey in Quebec province. Soumis pour publication, 2008.

Levesque J.-F, Pineault R, Simard B, Roberge D, Hamel M, Kapetanakis C, Robert L. L'expérience de soins de la population. Portrait des variations intra-régionales à Montréal et en Montérégie. Institut national de santé publique du Québec, Direction de santé publique de l'Agence de la santé et des services sociaux de Montréal et Centre de recherche de l'hôpital Charles LeMoyne. Rapport et résumé, mars 2007a (Résumé anglais : The population's experience of care: Portrait of intra-regional variations in Montréal and Montérégie).

Levesque J.-F., Pineault R., Robert L, Hamel M, Roberge D, Kapetanakis C, Simard B, Laugraud A. Les besoins non comblés de services médicaux : un reflet de l'accessibilité des services de première ligne? Institut national de santé publique du Québec et Direction de santé publique de l'Agence de la santé et des services sociaux de Montréal. Cahier thématique et résumé octobre 2007b. (Cahier thématique et résumé anglais : Unmet healthcare needs : A reflection of the accessibility of primary care services?).

Levesque J-F, Pineault R, Simard B, Hamel M, Roberge D, Ouellet D, Haggerty J. Questionnaire populationnel. L'accessibilité et la continuité des services de santé : Une étude sur la première ligne au Québec. Institut national de santé publique du Québec, Direction de santé publique de l'Agence de la santé et des services sociaux de Montréal et Centre de recherche de l'hôpital Charles LeMoyne, avril 2006. (version anglaise : Population questionnaire. Accessibility and continuity of health services: A study on primary care in Québec).

Levesque J-F, Roberge D, Pineault R. La première ligne de soins : un témoin distant des réformes institutionnelles et hospitalières au Québec? Chapitre 5 dans M-J Fleury, M Tremblay, H. Nguyen, L Bordeleau « Le système sociosanitaire au Québec : gouvernance, régulation et participation ». Gaëtan Morin éditeur, Chenelière Éducation, 2007, p. 63-78, ISBN : 2-89105-970-0.

Mazankowski D. A framework for reform, Report of the Premier's Advisory Council on Health, Government of Alberta, 2001, 72 pages.

Meyer AD, Tsui AS, Hinings CR. Configurational approaches to organizational analysis. Academy of Management Journal. 1993; 36(6):1175-1195.

Miller D. Configurations de stratégies et de structures : Un pas vers la synthèse. Gestion. 1996; 21(1):43-54.

Miller D. Environmental fit versus internal fit. Organization Science. 1992; 3(2):159-178.

Murray CJL, Frenk J. A framework for assessing the performance of health systems. Bulletin of the World Health Organization 2000;78 (6):717-731.

Nietert, PJ. et al. Using a summary measure for multiple quality indicators in primary care: the Summary Quality InDex (SQUID). Implementation Science, 2007;2(11):1-12.

Penchansky R, ThomasW. The concept of access, Defenition and relationship to consumer satisfaction. Medical Care 1981;19(1):127-140

Pineault R, Foisy L, Hamel M, Simard B, Levesque J-F, Roberge D. Répertoire des cliniques médicales de première ligne de Montréal et de la Montérégie. Institut national de santé publique du Québec, Direction de santé publique de l'Agence de la santé et des services sociaux de Montréal, 2006.

Pineault R, Hamel M, Levesque J-F, Roberge D, Lamarche P, Haggerty J. Questionnaire organisationnel Clinique médicale de première ligne. L'accessibilité et la continuité des services de santé : Une étude sur la première ligne au Québec. Direction de santé publique de l'Agence de la santé et des services sociaux de Montréal, 2005 (Version anglaise : Organisational questionnaire PHC clinic: A study on primary care in Québec).

Pineault R, Levesque J-F, Breton M, Hamel M, Roberge D. Réalisations et potentiel des organisations publiques et privées de première ligne dans deux régions du Québec. Dans Béland F, Contandriopoulos A-P, Quesnel-Vallée A, Robert L,. Le privé dans la santé : un débat sans fin? (à paraître 2008).

Pineault R, Levesque J-F, Tousignant P, Beaulne G, Hamel M, Poirier L-R, Raynault M-F, Bénigeri M, Roberge D, Lamarche P, Haggerty J, Hanley J, Bergeron P, Dulude S, Marcil M. (2004). L'accessibilité et la continuité dans la population: l'influence des modèles d'organisation des services de santé de première ligne. Projet financé par la Fondation Canadienne de Recherche sur les Services de Santé, 2004. FCRSS RC1-1091-05.

Pineault R, Levesque J.-F, Hamel M, Roberge D, Prud'Homme A, Perron M, Simard B, Foisy L. Le profil et le type d'organisation de première ligne ont-ils un lien dans l'expérience de soins de leur clientèle? Résultats d'une étude dans deux régions du Québec. Infolettre, février 2007, 4 (1):2-6.

Pineault R, Levesque J.-F, Hamel M, Roberge D, Prud'Homme A, Perron M, Simard B, Foisy L. Are the profile and type of organization of primary care linked to experience of care for their clients? Findings of a study in two regions of Quebec, Infoletter, February 2007, 4 (1):2-6.

Pineault R, Levesque J.F. Le type d'organisation des soins de première ligne a-t-il un effet sur l'expérience de soins rapportée par les utilisateurs? Le Point en administration de la santé et des services sociaux, Hiver 2007 – 2008, 3 (4): 12-14

Reid R, Haggerty J, McKendry R. Dissiper la confusion : Concepts et mesures de la continuité des soins. Rapport préparé pour la Fondation canadienne de la recherche sur les services de santé, 2002, 18 pages.

Roberge D, Pineault R, Hamel M, Da Silva R, Cazale L, Levesque J-F, Ouellet D, Prudhomme A. Rapport méthodologique de l'analyse des contextes. L'accessibilité et la continuité des services de santé : Une étude sur la première ligne au Québec. Institut national de santé publique du Québec, Direction de santé publique de l'Agence de la santé et des services sociaux de Montréal, Centre de recherche de l'hôpital Charles LeMoyne, décembre 2007.

Roberge D, Larouche D, Pineault R, Levesque J.-F, Hamel M, Simard B. L'urgence hospitalière : un substitut à la première ligne? Résultats d'une enquête auprès de la population de Montréal et de la Montérégie. Direction de santé publique de l'Agence de la santé et des services sociaux de Montréal, Institut national de santé publique du Québec et Centre de recherche de l'Hôpital Charles LeMoyne, Rapport et résumé, mars 2007 (Résumé anglais : Hospital emergency departments: Substitutes for primary care? Results of a survey among the population of Montréal and Montérégie.

Romanow RJ. Guidé par nos valeurs. L'avenir des soins de santé au Canada, rapport final. Saskatoon : Commission sur l'avenir des soins de santé au Canada; 2002.

Reid R, Haggerty J, McKendry R. Dissiper la confusion : Concepts et mesures de la continuité des soins. Rapport préparé pour la Fondation canadienne de la recherche sur les services de santé, 2002, 18 pages.

Safran DG, Kosinski M, Tarlov AR, Rogers WH, Taira DH, Lieberman N, Ware JE. The Primary Care Assessment Survey: tests of data quality and measurement performance. Med Care. 1998 May;36(5):728-39.

Safran DG, Tarlov AR, Rogers WH. Primary care performance in fee-for-service and prepaid healthcare systems. Results from the Medical Outcomes Study. JAMA, 1994 May 25;271(20):1579-86.

Savard I, Rodrigue J. Des omnipraticiens à la grandeur du Québec. Évolution des effectifs et des profils de pratique. Édition 2007.

Sinclair D. Health Services Restructuring Commission (Duncan Sinclair, Chair), Primary Healthcare Strategy - Advice and Recommendations to the Honourable Elizabeth Witmer, Minister of Health, Government of Ontario, December 1999, 170 pages.

Schoen C, Osborn R, Trang Huynh P, Doty M, Davis K, Zapert K, Peugh J, Primary Care and Health System Performance: Adults Experiences In Five Countries; Differing performance lever among

countries highlight the potential for improvement and cross-national learning. Primary Care Health Affairs; w4:487-503. 2004.

Scott WR, Mendel P, Pollack S. Environments and fields: Studying the evolution of a field of medical care organizations. In: Powell WW, Jones DL (eds). How Institutions Change. University of Chicago Press, forthcoming: Revised February 2004:2-38.

SOM. Sondage sur l'accessibilité et la continuité dans la population : l'influence des modèles d'organisation des services de première ligne. Rapport méthodologique présenté à la Direction de santé publique de l'Agence de la santé et des services sociaux de Montréal, à l'Institut national de santé publique du Québec et au Centre de recherche de l'hôpital Charles LeMoyne, Septembre 2005, 22 pages.

SPAD: logiciels pour l'Analyse des Données - Data Mining - Analyse Prédictive - Statistique Décisionnelle - Contrôle et Gestion de la Qualité des Données.

Starfield B. Primary care: Balancing Health Needs, Services and Technology. New York: Oxford University Press, 1998, 438 pages.

APPENDIX A – GLOSSARY

Accessibility of services

The notion of accessibility refers to the ease with which access to an institution or service is possible. Access signifies the possibility of obtaining a health service when there is a perceived need and a desire for care (Frenk, 1992; Penchansky and Thomas, 1981). Population access can be measured in terms of barriers to service utilisation through population surveys or patient perception surveys (Starfield, 1998).

In this study, a health organisation that can be used easily is considered accessible if there are few geographical, organisational, economic and cultural barriers to its use. Accessibility is the assessment of the possibility of using a service and ease with which the service or resource is used (Haggerty, Burge et al., 2008; Donabedian, 1973; Dussault, 1993).

Affiliation

Affiliation describes the action of affiliating with or joining an organisation. In this study, affiliation refers to the fact of having formal or informal links with a general practitioner or a primary care organisation.

Experience of care

In this study, experience of care refers to the way individuals feel about or perceive or the care and services received. The experience of care incorporates various attributes that can be evaluated by individuals (for instance, accessibility, continuity, comprehensiveness, responsiveness or outcome of services).

Comprehensiveness of services

The concept of comprehensiveness is used to described the services required to respond to a community's most common health needs. Comprehensive services are designed, based on the health needs presented, to address all personal (biological, psychological and social) or health-related dimensions (functional capacity, sensation, cognition), or all stages of intervention (promotion, prevention, diagnostic, treatment, palliation), Comprehensiveness of care is achieved by the availability, in an organisation, of a full range of services to meet patient's needs or by the assurance other services in other institutions are accessible (Starfield, 1998).

Continuity of care

Continuity describes the absence of a break in the temporal sequence during which several services are provided. These services are continuous if they flow smoothly one after the another. Continuity results from the attributes of resources that allow for such a progression: the same people provide the services, and information circulates between care episodes or among the different sites where services are offered (Reid et al., 2002).

Continuity encompasses three dimensions: informational (transfer and accumulation of information required for a person's treatment); clinical (approach) (care given over a coherent temporal sequences); and relational (patient-professional relationship is stable over time) (Haggerty, Burge et al., 2008; Reid, Haggerty et al., 2002).

Family Medicine Group

A Family Medicine Group (FMG) is a group of 6 to 10 physicians—for 10 000 to 20 000 people—who work very closely with nurses to provide services to people registered with a Group, on a non-geographical basis. An FMG provides services by appointment or on a walk-in basis; it is accessible 24 hours a day, 7 days a week through opening hours that extend into the evening (until 9:00 p.m.) and weekends (at least 4 hours), and through an on-call system when the clinic is closed. FMG physicians work in cooperation with on-site nurses and community services, according to the terms negotiated with a local community services centre (CLSC). In 2008, there were about 145 FMG in Québec, 43 of which are in the two regions considered in the study.

Family physician

For the purposes of this study, the notion of family physician refers to a respondent identifying a “family doctor”. No specific definition was provided to respondents during the survey. They were asked a direct question, "Do you have a family doctor?" and two indirect questions, "At this place, is there a specific doctor that manages your health? Do you think of this physician as your family doctor?” to determine if they had a regular family physician.

First contact

In this study, first contact describes use of services initiated by individuals. First contact is the first medical assessment performed after a person has recognized a need for health services and he or she has sought care.

Network clinic

The services offered by these institutions are more specifically targeted to (1) ongoing and integrated management of clients, particularly vulnerable clienteles; (2) access to primary care at all times and to urgent use of technical support centres and specialists (Gouvernement du Québec, 2006). In short, with regard to services offered, network clinics add a broader range of services to those offered in FMG, including basic technical support and access to specialises services. Initially, the plan was to implement 15 to 20 network clinics in Montréal. By January 2006, there were 13. The MSSS supported their development, an essential element to consolidating primary care services. In 2007, there were 16 network clinics in Montréal.

Organisation

The definition of “organisation” used in this study refers to organisational entities that include one or several general practitioners offering general medical services. Therefore private, single-doctor

offices are regarded as “organisations”. Offices and clinics with more than one physician are also "organisations” when physicians share a minimum number of administrative resources (rooms, secretarial services or archives), whether or not their professional practices are integrated. Service points tied to a CLSC but located at different sites and having their own medical equipment are considered to be separate organisations.

Perceived outcome of services

In this study, perceived outcome of services describe the effects or consequences of health services on the health of persons, as perceived by the individuals. These effects can include direct consequences on perceived state of health or consequences on health related knowledge. Moreover, perceived outcomes of care can involve intermediate results—such as adoption of health behaviours—in addition to health outcomes.

Primary medical care services

The notion of primary care refers to the presence of a combination of resources and services that are consulted initially when there is a health problem. According to this definition, primary care includes general medical care that is aimed at diagnosing common conditions and assessing conditions that will require specialised care. Primary care medical services are health services that are located close to individuals and communities and possess light diagnostic capabilities Given the wide range of needs that can bring a person to turn to the health system, a number of health professionals dispense primary care medical services, including physicians, nurses, dentists, optometrists, physiotherapists, psychologists and pharmacists. Some specialists also offer primary care services, insofar as patients can seen them for all sorts of health problems (paediatricians for children, gynaecologists/obstetricians for reproductive care and births, and internists in some settings).

For purposes of this study, primary care medical services refers to places where family physicians, generalists or general practitioners offer first contact care (medical evaluation): doctors offices, medical clinics and polyclinics, family medicine groups (FMG), affiliated or integrated network clinics, family medicine units in CLSC and hospital centres, and hospital emergency units.

Regular source of care

For the purposes of this study, the regular source of care is the one reported by an individual as the place “you usually go to see a doctor for your general medical care, excluding specialised care". In situations where respondents do not identify a usual place, the place where they went most frequently in the past two years is designated as the usual source of care.

Responsiveness of services

Responsiveness is a concept put forward by the World Health Organization (Murray, Frenk, 2000) which consists in the response to legitimate expectations of the population concerning

elements or actions not related to the technical content of treatment. There are two major components to responsiveness: respect for persons (defined as individuals' interactions with the health system) and client orientation (includes several dimensions of consumer satisfaction that are not a function of health improvement.

Respect for persons comprises respect for dignity, for confidentiality, that is, the patients' right to have access to written and verbal information about their own health, and for autonomy, that is, the possibility of participating in choices concerning their health, including methods of investigation and treatment that will or will not be received.

The notion of client orientation includes prompt attention in emergency situations and reasonable delays in cases that are not urgent, a good quality environment including clean and spacious rooms and adequate food, access to social support networks during and after care, and choice of care provider (institution or individual providing care) (Murray and Frenk, 2000).

Taxonomy of primary care organisation

Grouping of primary care organisations into homogeneous categories that are clearly differentiated according to their vision, resources, organisational structure and organisational practices.

Unmet needs for care

The notion of unmet needs for care describes a situation where a person perceives or feels a need to obtain health services but does not.

APPENDIX B – METHODOLOGICAL ASPECTS

B1 Indices of experience of care
B2 Taxonomy of primary care organisation
B3 Taxonomy of contexts
B4 Ideal-type conformity score
B5 Analyses of the relationships among organisational models, experience of care, having a family physician and unmet needs for care

B1 Indices of experience of care

Factorial construction of indices of experience of care

For the purposes of our study, we needed to develop indices for the different effects expected from primary care organisational models. The population-based questionnaire offered an extensive series of questions that are both reporting (questions used to report facts) and rating (questions used to assess perceived quality, satisfaction or agreement with a statement) in nature. A limited number of effects that covered the range of experience of care had to be developed for the analyses. Therefore, we elaborated synthetic indices of various concepts related to individuals' experience of care.

Starting from the questions on experience of care with the regular source of care, about 40 items were selected on a conceptual judgement basis. These items were selected according to the concepts identified originally: geographical and organisational accessibility, accessibility of first contact, economic accessibility, continuity of affiliation and follow-up, informational continuity, comprehensiveness, responsiveness and perceived outcomes of care (Table 1).

The frequency distribution of each item was evaluated to consider the items' various response scales and assess their compatibility. Only items with four-category response scales were retained for analysis. A measure of inter-item correlation was also performed.

Exploratory factor analyses were then conducted. We used the principal component extraction method and orthogonal solutions (Varimax). These initial analyses, which included only respondents who had answered all questions in the scale, focused on all items in the experience of care scale and each dimension previously determined by conceptual classification. The factor solution only partly reproduced the classifications of the prior conceptual attribution. When related to the conceptual model, these results enabled us to group the dimensions or items together to better reflect the concepts that were actually evaluated. Some items with little commonality with the factors (0.30) or strong links without adding more specific information were removed from the scale.

Additional exploratory factor analyses were then conducted, again using the principal component (PC) extraction method and orthogonal solutions (Varimax). The internal homogeneity of the global scales and of its dimensions was evaluated by Cronbach's alpha. These latter analyses allowed confirmation that the new grouping of items, excluding 10 that had to be removed, constituted a valid factorial classification.

The factor analyses generated the following eight indices of experience of care: *physical and organisational accessibility* (5 items, Cronbach's alpha: 0.45); *economic accessibility* (3 items; Cronbach's alpha: 0.42); *continuity of affiliation* (4 items; Cronbach's alpha: 0.70); *informational continuity* (3 items; Cronbach's alpha: 0.57); *responsiveness* (5 items; Cronbach's alpha: 0.70); *comprehensiveness* (4 items; Cronbach's alpha: 0.78); *perceived outcomes of care* (5 items; Cronbach's alpha: 0.84); as well as an aggregate index of experience of care (29 items; Cronbach's alpha: 0.87).

The various indices present moderate to significant correlations among each other. There are high correlations, varying between 0.64 and 0.75, among indices of *responsiveness*, *comprehensiveness* and *perceived outcomes of care*. Indices of *continuity of affiliation* and *informational continuity* are moderately correlated with other indices of experience of care, ranging from 0.35 to 0.58. The *physical*

and organisational index is also moderately correlated with *comprehensiveness* (0.34) and *responsiveness* (0.36). Finally, *economic accessibility* shows weak correlations with the other indices of experience of care.

For each experience of care index, two aggregate scores were constructed: factor and performance threshold. The factor score is computed by adding the values of the original scale. The threshold performance score is computed from dichotomised variables by adding the value 1 of each variable. A group of experts established a dichotomisation point that generally corresponds to the upper category of the original scale, which is given the value 1, while the others are assigned the value 0. This performance score is called SQUID (Summary QUality InDex) (Feifer et al., 1997; Neitert et al, 2007). The two scores are expressed as percentages, either by dimension or for dimensions as a whole.

Table B presents details of the composition of experience of care indices.

Table B1: Composition of indices of experience of care

Geographical and organisational accessibility index alpha: 0.45; explained variance: 54.2%

Wording of questions	Original scale	Dichotomisation	%
The clinic is conveniently located	1) Strongly,<br>2) Somewhat,<br>3) A little,<br>4) Not at all,<br>95) Does not know	1 = Strongly<br>0 = Other	79.9<br>20.1
The office hours are convenient	1) Strongly,<br>2) Somewhat,<br>3) A little,<br>4) Not at all,<br>95) Does not know	1 = Strongly<br>0 = Other	60.3<br>39.7
It is easy to reach someone at this place by telephone to make an appointment	1) Strongly,<br>2) Somewhat,<br>3) A little,<br>4) Not at all,<br>95) Does not know	1 = Strongly and somewhat<br>0 = Other	57.8<br>42.2
It is easy to talk to a doctor or nurse by telephone when this place is open	1) Strongly,<br>2) Somewhat,<br>3) A little,<br>4) Not at all,<br>95) Does not know	1 = Strongly and Somewhat<br>0 = Other	18.7<br>81.3
It is easy to enter the building where the clinic is located (stairs, elevator, access ramp, etc.)	1) Strongly,<br>2) Somewhat,<br>3) A little,<br>4) Not at all,<br>95) Does not know	1 = Strongly<br>0 = Other	88.7<br>11.3

Economic accessibility index alpha: 0.42; explained variance: 46.4%

Wording of questions	Original scale	Dichotomisation	%
When you go to this place, you have to pay for laboratory or radiology tests done on site or somewhere else (e.g., blood tests, X-ray, scan, mammography, etc.)	1) Always,<br>2) Often,<br>3) Sometimes,<br>4) Never,<br>95) Does not know	1 = Never<br>0 = Other	73.7<br>26.3
When you go to this place, you have to pay for OTHER doctor's services not covered by the Régie de l'assurance-maladie or not reimbursed by your personal health insurance plan (for example to get the doctor to fill out a form for you)	1) Always,<br>2) Often,<br>3) Sometimes,<br>4) Never,<br>95) Does not know	1 = Never<br>0 = Other	73.7<br>26.3
When you go to this place, you have to pay for medications given on site or material such as bandages, syringes, etc.? (For example eye drops, local anaesthetic, etc.)	1) Always,<br>2) Often,<br>3) Sometimes,<br>4) Never,<br>95) Does not know	1 = Never<br>0 = Other	89.4<br>10.6

Continuity of affiliation index alpha: 0.70; explained variance: 53.8%

Wording of questions	Original scale	Dichotomisation	%
When you go this place, you see the same doctor	1) Always,<br>2) Often,<br>3) Sometimes,<br>4) Never,<br>95) Does not know	1 = Always<br>Often<br>0 = Other	75.0<br>25.0
Still keeping in mind your experiences over the last two years, tell me if you agree strongly, somewhat, a little or not al all with each of the following statements. At this place…<br>your medical history is known	1) Strongly,<br>2) Somewhat,<br>3) A little,<br>4) Not at all,<br>95) Does not know	1 = Strongly<br>0 = Other	70.7<br>29.3
Still keeping in mind your experiences over the last two years, tell me if you agree strongly, somewhat, a little or not al all with each of the following statements. At this place… the clinic professionals are aware of all the prescription medications you take	1) Strongly,<br>2) Somewhat,<br>3) A little,<br>4) Not at all,<br>95) Does not know	1 = Strongly<br>0 = Other	79.4<br>19.6
At this place… you can get routine ongoing care for a chronic problem, for example, for high blood pressure (hypertension), diabetes or back pain, etc.	1) Strongly,<br>2) Somewhat,<br>3) A little,<br>4) Not at all,<br>95) Does not know	1 = Strongly<br>0 = Other	69.6<br>30.4

Informational continuity index alpha: 0.57; explained variance: 55.7%

Wording of questions	Original scale	Dichotomisation	%
Someone telephoned you or met with you to give you the results of these tests or exams	1) Always,<br>2) Often,<br>3) Sometimes,<br>4) Never,<br>95) Does not know	1 = Always/Often<br>0 = Other	67.0<br>33.0
You were given clear explanations about the results of your tests or exams	1) Always,<br>2) Often,<br>3) Sometimes,<br>4) Never,<br>95) Does not know	1 = Always<br>0 = Other	75.2<br>24.8
After you saw the specialist(s) (or another general practitioner), a doctor at the clinic discussed the report with you	1) Always,<br>2) Often,<br>3) Sometimes,<br>4) Never,<br>95) Does not know	1 = Always<br>0 = Other	51.1<br>49.9

Responsiveness alpha: 0.70; explained variance: 46.6%

Wording of questions	Original scale	Dichotomisation	%
You feel respected	1) Strongly,<br>2) Somewhat,<br>3) A little,<br>4) Not at all,<br>95) Does not know	1 = Strongly<br>0 = Other	86.0<br>14.0
You are greeted courteously at the reception	1) Strongly,<br>2) Somewhat,<br>3) A little,<br>4) Not at all,<br>95) Does not know	1 = Strongly<br>0 = Other	73.7<br>26.3
Your physical privacy is respected	1) Strongly,<br>2) Somewhat<br>3) A little,<br>4) Not at all,<br>95) Does not know	1 = Strongly<br>0 = Other	90.7<br>9.3
At this place or at this clinic, the doctors spend enough time with you	1) Strongly,<br>2) Somewhat,<br>3) A little,<br>4) Not at all,<br>95) Does not know	1 = Strongly<br>0 = Other	67.1<br>32.9
The premises are pleasant	1) Strongly,<br>2) Somewhat,<br>3) A little,<br>4) Not at all,<br>95) Does not know	1 = Strongly<br>0 = Other	69.0<br>31.0

Care outcome alpha: 0.84; explained variance: 63.4%

Wording of questions	Original scale	Dichotomisation	%
Your questions are answered clearly (all the clinic staff)	1) Strongly,<br>2) Somewhat,<br>3) A little,<br>4) Not at all,<br>95) Does not know	1 = Strongly<br>0 = Other	77.2<br>22.8
The services you get there help you better understand your health problems	1) Strongly,<br>2) Somewhat,<br>3) A little,<br>4) Not at all,<br>95) Does not know	1 = Strongly<br>0 = Other	68.4<br>31.6
The services you get there help you prevent certain health problems before they develop	1) Strongly,<br>2) Somewhat,<br>3) A little,<br>4) Not at all,<br>95) Does not know	1 = Strongly<br>0 = Other	55.0<br>45.0
The services you get there help you control your health problems	1) Strongly,<br>2) Somewhat<br>3) A little,<br>4) Not at all,<br>95) Does not know	1 = Strongly<br>0 = Other	68.3<br>31.7
The professionals you see there help motivate you to adopt good lifestyle habits like quitting smoking, eating better, etc.	1) Strongly,<br>2) Somewhat,<br>3) A little,<br>4) Not at all,<br>95) Does not know	1 = Strongly<br>0 = Other	65.1<br>34.9

Comprehensiveness alpha: 0.78; explained variance: 61.1%

Wording of questions	Original scale	Dichotomisation	%
At this place… during your visits, the doctor takes the time to talk to you about prevention and asks you about your lifestyle habits	1) Strongly,<br>2) Somewhat,<br>3) A little,<br>4) Not at all,<br>95) Does not know	1 = Strongly<br>0 = Other	61.7<br>38.3
At this place… you get help for all the healthcare services you need	1) Strongly,<br>2) Somewhat,<br>3) A little,<br>4) Not at all,<br>95) Does not know	1 = Strongly<br>0 = Other	73.0<br>27.0
Your opinion and what you want are taken into account in the care that you receive	1) Strongly,<br>2) Somewhat,<br>3) A little,<br>4) Not at all,<br>95) Does not know	1 = Strongly<br>0 = Other	74.1<br>25.9
You are given help to weigh the pros	1) Strongly,	1 = Strongly	70.3

Wording of questions	Original scale	Dichotomisation	%
and cons when you have to make decisions about your health	2) Somewhat,<br>3) A little,<br>4) Not at all,<br>95) Does not know	0 = Other	29.7

Global index including the 29 experience of care items
Cronbach's alpha: 0.872
Explained variance: 53.8%

B2 Taxonomy of primary care organisation

B2.1 Methods

The method used in this study is based on multidimensional exploratory statistics. This strategy allows consideration of a large quantity of information and production of a synthesis. The objective of these analyses was to characterise homogeneous classes of primary care organisation services. The analyses were conducted in accordance with the following steps.

(1) **The construction of organisational variables** is based on the concept of organisational conceptualisation proposed by Lamarche, Pineault et al., 2003. Particular attention was paid early on to transforming continuous variables into categorical ones. Qualitative coding of all variables was performed on the basis of two principles: (1) the existence of a natural or conventional threshold; and (2) a search for equal numbers within modalities. 43 variables were constructed and arranged into the following dimensions: vision (9 variables) resources (7 variables), structure (10 variables) and practices (17 variables). The list of variables, their values and distributions are presented in Table B2.

(2) **Factor analyses** were performed to study existing relationships between the variables and their modalities, and to confine the information to factorial spaces. Multiple correspondence analyses (MCA) are particularly interesting when highlighting non-linear links among variables. MCA carried out by dimension underscored the most striking data structures (factorial axes), coherent with initial conceptual positioning. In addition to statistical indicators associated with inertia values (explained variance) of different factors and with dispersion of individuals and variables, many of the decisions pertaining to number of factors to retain depended on the interpretation of factors, as recommended by Lebart, Morineau and Piron (2000), who attach a lot of importance to knowledge about the study subject. Finally a principal components analysis (PCA) enabled development of a complete final model that integrates the factorial axes of each dimension.

(3) The **ascending hierarchical classification** has proven to be an effective strategy to identify partition groups for which the variance within each class is minimal and variance between classes is maximal. This method uses a technique based on similarities among individuals based on Euclidian distance, and a

second technique that proceeds by aggregation, applying the generalised Ward criterion, which is based on minimum loss of inertia (or variance) (Lebart, Morineau and Piron, 2000).

(4) **Choice of final classification** rests on comparison of statistical analysis results expressed as inter-class distances and inertia quotient with theoretical plausibility of the groupings. The main indicators used are the following (Lebart, Morineau and Piron, 2000):

- Intra-class inertia: intra-class measure of variance that expresses the degree of homogeneity in the category
- Inter-class inertia: inter-class measure of variance that expresses the degree of heterogeneity among categories
- Distance: square of the distance of the chi-square of the origin to the class's centre of gravity. "The higher the value, the more the class is atypical and contains individuals who are very different from the average individual in the sample."
- Inertia quotient: inter-class inertia/total inertia (variance ratio).

Two criteria were used to determine optimal grouping choices, with the objectives of minimising intra-class variance or inertia and maximising inter-class variance or inertia. The first is based on the variance ratio or inertia quotient. We look for the threshold at which there are no longer any significant increases or substantial gains. The second concerns group differentiation and consists in identifying the group for which adding classes does not bring additional significant statistical or theoretical information.

(5) **Description and interpretation of organisational models** looks at modalities according to their degree of significance. A high test value (> 2; absolute value) means that this modality is significantly important for characterisation of the grouping. A test-value of 2 corresponds to the 1% threshold or critical probability of 0.01. The following three indicators are also used at this stage:

- % of the modality in the class: % of organisations in the class with the modality,
- % of the modality in the sample: % of organisations in entire sample with the modality,
- % of the class in the modality: % of organisations in the class with the modality, among all organisations with this modality.

Table B2: Taxonomy of organisations – Organisational variables

Variables	Wording of the variable	Categories (%; 473 organisations)			
**Vision**					
**V1**	Scope of responsibility	1. More socio-environmental (63.2)	2. Less socio-environmental (36.8)		
**V2**	Population targeted to be served by the clinic	1. Population (12.5)	2. Clinic's regular clients (75.1)	3. Individuals (12.5)	
**V3**	Clinic's priority regarding service organization	1. Continuity of care (79.7)	2. Accessibility (20.3)		
**V4**	Importance attached to experience of care	1.More important (33.2)	2. Moderately important (35.3)	3. Less important (31.5)	
**V5**	Responsibility and right regarding health and access to services	1. More social (44.2)	2. Moderately social (36.8)	3. Less social (19.0)	
**V6**	Financial profitability	1. Less important (47.6)	2. More important (52.4)		
**V7**	Valued team work	1. More important (48.6)	2. Less important (51.4)		
**V8**	Responsibility towards work colleagues and clinic managers	1. More important (48.4)	2. Less important (51.6)		
**V9**	Responsibility towards the RAMQ and the CMQ	1. More important (68.9)	2. Less important (31.1)		
**Resources**					
**R1**	Number of FTE MDs in this clinic	1. > 6 FTE (19.5)	2. 4-6 FTE (20.5)	3. 2-3 FTE (23.5)	4. ≤ 1 FTE (36.6)
**R2**	Presence of a nurse	1. Yes (36.6)	2. No (63.4)		
**R3**	Presence of other health professionals or medical specialist MDs	1. Medical specialist with or without health professionals (52.2)	2. Health professional only (26.2)	3. None (21.6)	
**R4**	Financing sources for the operating costs of the clinic	1. Public source only (11.4)	2. Physician and public source (7.4)	3. Physician source only (81.2)	
**R5**	Financial contribution from patients or enterprises	1. No (87.9)	2. Yes (12.1)		
**R6**	Number of information's technologies	1. 3 tech. and + (18.0)	2. 2 tech. (24.9)	3. 1 tech. (27.3)	4. None (29.8)
**R7**	Availability of a technical platform on the site of the clinic	1. Radiology with or without bio-logical specimen collection (10.4)	2. Biological specimen collection only (39.5)	3. No technical platform (50.1)	
**Organisational structure**					
**S1**	Type of governance	1. Public (11.6)	2. Private (88.4)		
**S2**	Medico-administrative management	1.Administrator/ manager and physician (13.5)	2. Physician-in-charge and physician group (42.7)	3. Not specified and solo practice physician (43.8)	
**S3**	Seniority of physicians	1. > 5 years (77.0)	2. <= 5 years (23.0)		

Variables	Wording of the variable	Categories (%; 473 organisations)			
**S4**	Attendance time of the physician in the clinic	1.Long (58.6)	2.Short (41.4)		
**S5**	Mode of remuneration	1. Fee-for-service (88.4)	2. Time based and various modes (11.6)		
**S6**	Administrative resources sharing (rooms, staff, medical records)	1. Highly (31.1)	2.Moderately (31.5)	3. None (37.4)	
**S7**	Mechanism of care coordination among clinic professionals	1. Formal (27.7)	2. Informal (30.0)	3. None (42.3)	
**S8**	Collaborative agreement with PHC organizations (Clinic and CLSCS)	1 Yes (49.7)	2. No (50.3)		
**S9**	Collaborative agreement with hospital	1. Yes (48.8)	2. No (51.2)		
**SA**	Participation of physicians in regional committees	1. Yes (40.2)	2. No (59.8)		
**Organisational practices**					
**P1**	Availability of services in the evening and on the week-end	1. High (16.1)	2. Moderate (24.5)	3. Low (59.4)	
**P2**	Possibility of regular patients of the clinic who have an urgent health problem to contact a nurse or a physician	1.High (11.4)	2. Moderate (55.2)	3. Low (33.4)	
**P3**	Participation in a healthcare access network	1. Yes (9.3)	2. No (90.7)		
**PZ**	Types of consultation	1. + scheduled appointments (61.7)	2. Various type (app. and walk-in) (15.6)	3. + walk-in clinics (22.6)	
**P5**	Number of settings where the clinic's services are available (home, hospital or residential centres)	1. 2-3 settings (21.6)	2. 1 setting (23.0)	3. None (55.4)	
**P6**	Approximate time for a consultation at the clinic	1. Long (30.4)	2. Moderate (31.9)	3. Short (37.6)	
**P7**	Range of services available on the site of the clinic (Diagn./prevention)	1. Very extended (27.3)	2.Extended (30.2)	3. Restricted (42.5)	
**P8**	Number of diagnostic and therapeutic procedures available on the site of the clinic	1. Much (7-10) (27.9)	2. Fairly (4-6) (31.3)	3. A little or none (0-3) (40.8)	
**P9**	Management of chronic diseases	1. Overall responsibility (33.8)	2. Limited responsibility (66.2)		
**PA**	Assistance by professionals for appointments with specialist	1. + support (35.7)	3. - support (64.3)		
**PB**	Role of the nurse	1. Extended role	2.Limited role	3. No nurse in the	

Variables	Wording of the variable	Categories (%; 473 organisations)			
		(Innovator) (22.4)	(traditional) (14.2)	clinic (63.4)	
**PC**	Sharing of clinical activities (among MDs)	1. More important (49.9)	2. Less important (50.1)		
**PD**	Network of reference with PHC clinics	1. Extended (70.6)	2. Limited (29.4)		
**PE**	Network of reference with hospitals	1.Extended (55.2)	2. Limited (44.8)		
**PF**	Network of reference with private institutions	1. Extended (76.1)	2. Limited (23.9)		
**PG**	Organizational affiliation of the physicians (front and second lines)	1. Double affiliation (44.6)	2. Single affiliation (40.0)	3. None (15.4)	
**PH**	Maintenance and evaluation of professional competence	1. + mechanisms (20.5)	2. +/- mechanisms (33.8)	3. - mechanisms (45.7)	

B2.2 Factor analyses

MCA revealed the most striking data structures for each conceptual dimension For vision, four factorial axes were retained with 46% of explained inertia; for resources, four axes were retained with 47% of explained inertia; for structure, there were three axes with 52% of explained inertia; and for practices, four axes with 31% of explained inertia. A principal components analysis (PCA) enabled the development of a complete final model that integrates the factorial axes of each dimension. Fifty-six per cent of inertia is explained.

B2.3 Classification of organisations and description of groupings

Ascending hierarchical classification analysis techniques were used to group organisations into homogeneous, well-differentiated classes. The resultant classification tree is illustrated in Figure B1.

Two criteria were used to determine optimal grouping choices, with the objectives of minimising intra-class variance or inertia and maximising inter-class variance or inertia. The first is based on the inertia quotient. Results shown in Figure B2 demonstrate that an increase in inertia quotient with the number of classes tends to flatten out once 5 or 6 partitions are reached. After 6 partitions, the gains are not substantial.

Figure B1: Classification tree of organisations

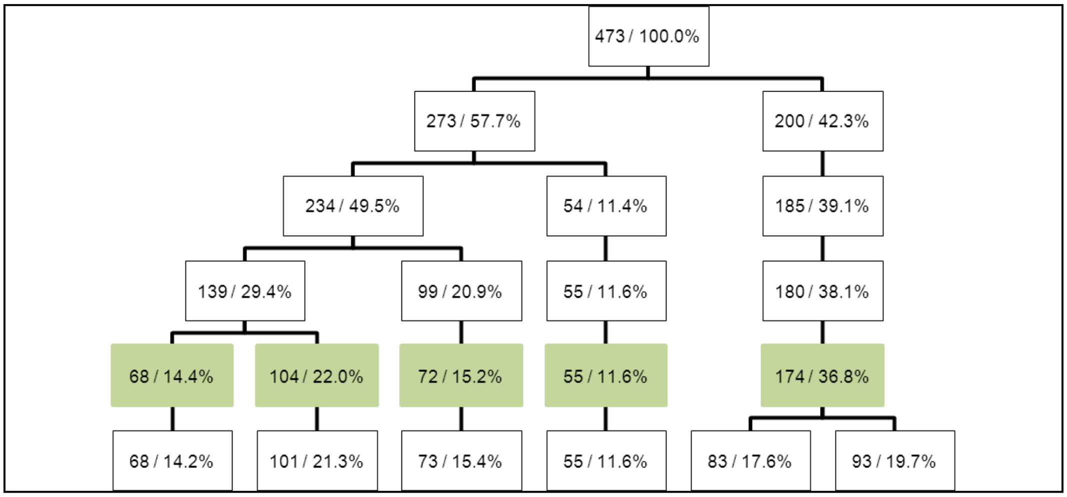

Corrected / Marsh 2012

Figure B2: Variation of the inertia quotient based on number of classes

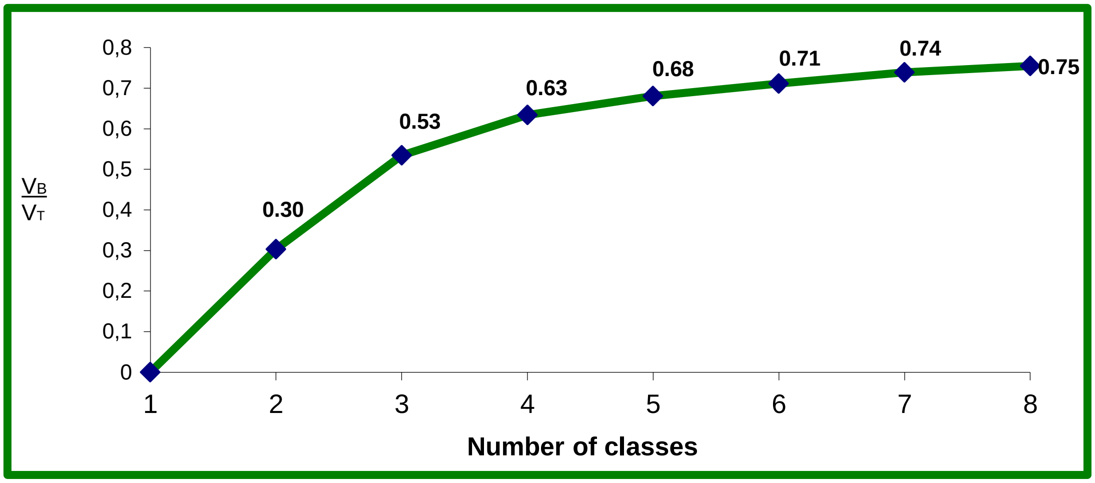

The second concerns group differentiation. Analysis of the distances among classes shows that in groupings with more than five classes, some groups are very close (therefore differ very little). Moreover the composition of classes (significant modalities and variables; test-values ≥2) is not greatly improved.

On these bases, groupings into five classes were deemed optimal. Intra-class inertia values (variance) and inter-class distances are shown in Figure B3.

Figure B3: Groupings of organisations into five classes: intra-class variance (IV) and inter-class distances

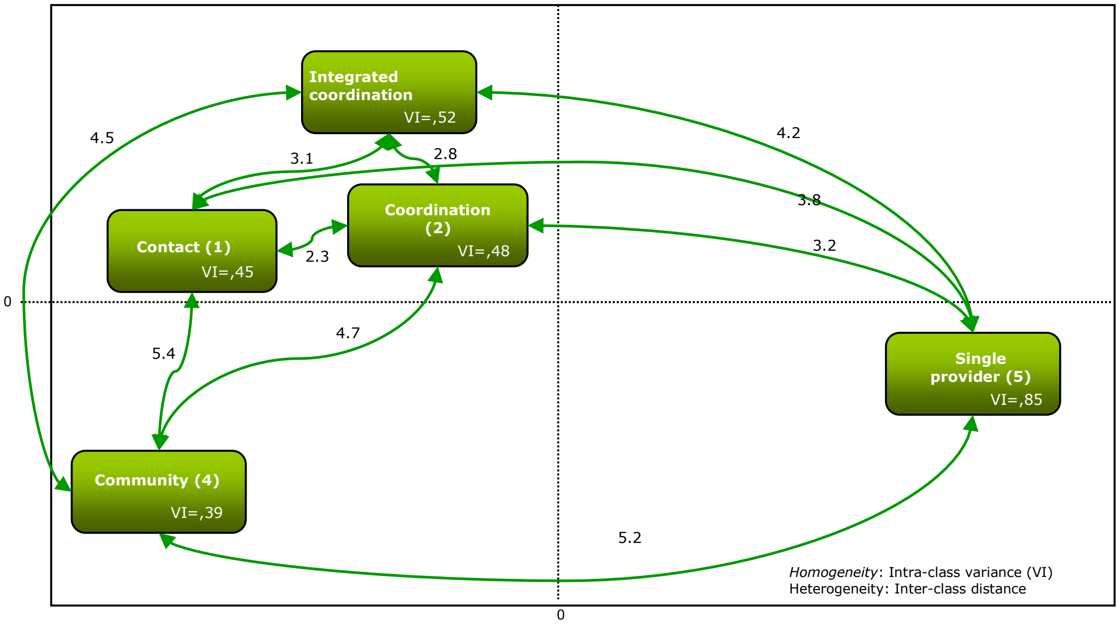

Table B3: Description of organisational groupings

Class: Class 1/5 (Workforce: 68 – Percentage: 14.38)

***Professional contact* model**			
**Wording of variables**	**Characteristic modalities**	**Test value**	**Histogram**
**Vision**			
(V2) Population targeted to be served by the clinic	3. Individuals	7.19	************
(V3) Clinic's priority regarding service organization	2. Accessibility	6.74	***********
(V1) Scope of responsibility	2. - Socioenvironmental	5.74	**********
(V8) Resp. towards work colleagues and clinic managers	1. More important	5.24	*********
(V4) Importance attached to experience of care	3. Less important	3.85	******
(V9) Resp. towards the RAMQ and the CMQ	1. More important	2.85	*****
(V6) Financial profitability	2. More important	2.07	***
**Resources**			
(R7) Availability of a technical platform on the site of the clinic	1. Radio with/without	5.42	*********

***Professional contact* model**			
**Wording of variables**	**Characteristic modalities**	**Test value**	**Histogram**
(R3) Presence of other health prof. or medical specialist MDs	1. Medical specialist with	5.15	*********
(R4) Financing sources for the operating costs of the clinic	3. Physician source only	5.08	********
(R1) Number of FTE MDs	2. 4-6 FTE	2.66	****
**Structure**			
(S6) Adm. resources sharing (rooms, staff, etc.)	1. Highly	4.48	*******
(S2) Medico-administrative management (organisational)	1. Administrator/manager and physician	3.96	*******
(S7) Mechanism of care coordination among clinic professionals	2. Informal	3.89	******
(S4) Attendance time of the physician in the clinic (Majori)	2. Short	3.78	******
(S1) Type of governance	2. Private	3.71	******
(S5) Mode of remuneration	1. Fee-for-service	3.71	******
(S8) Collaborative agreement with PHC organizations (Clinic and CLSCs)	2. No	3.25	*****
(S9) Collaborative agreement with hospital	2. No	2.83	*****
(S3) Seniority of physicians	2. <= 5 years	2.07	***
**Practices**			
(PZ) Types of consultation	3. + walk-in clinics	8.91	***************
(P5) Number of settings where the clinic's services are available (home, hospital or residential centres)	3. None	5.80	**********
(PH) Maintenance and evaluation of professional competence	2.+/- mechanisms	5.79	**********
(P2) Possibility for regular patients of the clinic who have an urgent health problem to contact a nurse or a physician	3. Low	5.34	*********
(PC) Sharing of clinical activities	1. More important	5.26	*********
(P9) Management of chronic diseases	2. Limited responsibility	4.98	********
(PA) Assistance by professionals for appointments with specialist	2. - support	3.67	******
(PE) Network of reference with hospitals	2. Limited	3.16	*****
(P7) Range of services available on the site of the clinic (diagn./prevention)	3. Restricted	2.80	*****
(PB) Role of the nurse	2. Limited role (tradi.)	2.78	*****
(P6) Approximate time for a consultation	3. Short	2.65	****
(PG) Organizational affiliation of the physicians	1. Double affiliation	2.41	****
(P1) Availability of services in the evening and on the week-end	2. Moderate	2.32	****
(P8) Number of diagnostic and therapeutic procedures available on the site of the clinic	1. Much (7-10)	2.15	****

Class : Class 2 / 5 (Workforce: 104 - Percentage: 21.99)

Professional coordination model			
**Wording of variables**	**Characteristic modalities**	**Test value**	**Histogram**
**Vision**			
(V7) Valued team work	1. More important	5.84	***********
(V8) Resp. towards work colleagues and clinic managers	1. More important	4.51	*******
(V2) Population targeted to be served	2. Population	3.64	******
(V3) Clinic's priority regarding service organization	1. Continuity of care	2.78	*****
(V4) Importance attached to experience of care	1. More important	2.10	***
**Resources**			
(R1) Number of FTE MDs	3. 2-3 FTE	8.43	***************
(R4) Financing sources for the operating costs of the clinic	3. Physician source only	6.10	***********
(R3) Presence of other health professionals or medical specialist MDs	1. Medical specialist with	3.86	******
(R7) Availability of a technical platform on the site of the clinic	2. Biological specimen collection only.	3.02	*****
(R1) Number of FTE MDs	2. 4-6 FTE	2.72	*****
(R2) Presence of a nurse	2. No	2.71	****
**Structure**			
(S7) Mechanism of care coordination among clinic professionals	2. Informal	6.63	************
(S2) Medico-administrative management	2. Physician-in-charge and physician group	6.08	***********
(S1) Type of governance	2. Private	4.93	*********
(S5) Mode of remuneration	1. Fee-for-service	4.93	*********
(S6) Administrative resources sharing (rooms, staff, medical records)	1. Highly	4.49	*******
(S6) Administrative resources sharing (rooms, staff, medical records)	2. Moderately	3.46	******
(S9) Collaborative agreement with hospital	2. No	3.19	*****
(S8) Collaborative agreement with PHC organizations (clinic and CLSCs)	2. No	2.26	****
**Practices**			
(PH) Maintenance and evaluation of professional competence	2. +/- mechanisms	7.42	*************
(PC) Sharing of clinical activities	1. More important	6.25	***********
(PF) Network of reference with private institutions	1. Extended	4.94	*********
(PE) Network of reference with hospitals	1. Extended	4.58	*********
(P7) Range of services available on the site of the clinic (diagn./prevention)	2. Extended	4.03	*******
(P3) Participation in a healthcare access network	2. No	3.62	******
(P6) Approximate time for a consultation at the clinic	3. Short	3.26	*****

Professional coordination model			
**Wording of variables**	**Characteristic modalities**	**Test value**	**Histogram**
(PB) Role of the nurse	3. No nurse	2.71	****
(P8) Number of diagnostic and therapeutic procedures available on the site of the clinic	2. Fairly (4-6)	2.35	****
(P2) Possibility for regular patients of the clinic who have an urgent health problem to contact a nurse or a physician	3. Low	2.04	***
(PG) Organizational affiliation of the physicians	1. Double affiliation	2.03	***

Class : Class 3 / 5 (Workforce: 72 - Percentage: 15.22)

Professional integrated coordination model			
**Wording of variables**	**Characteristic modalities**	**Test value**	**Histogram**
**Vision**			
(V7) Valued team work	1. More important	7.36	************
(V8) Resp. towards work colleagues and clinic managers	1. More important	7.10	************
(V2) Population targeted to be served by the clinic	1. Population	3.41	******
(V6) Financial profitability	2. More important	2.52	****
(V1) Scope of responsibility	1. More socioenvironm.	2.16	****
**Resources**			
(R4) Financing sources for the operating costs of the clinic	2. Physician and public source	9.72	****************
(R2) Presence of a nurse	1. Yes	6.31	**********
(R1) Number of FTE MDs	1. > 6 FTE	5.80	**********
(R6) Number of information's technologies	1. 3 tech. and +	4.79	********
(R3) Presence of other health prof. or medical specialist MDs	1. Medical specialist with	4.43	*******
(R7) Availability of a technical platform on the site of the clinic	1. Radio with/without	3.47	******
(R1) Number of FTE MDs	2. 4-6 FTE	2.66	****
**Structure**			
(S8) Collaborative agreement with PHC organizations (clinic and CLSCs)	1. Yes	7.17	************
(S9) Collaborative agreement with hospital	1. Yes	7.02	************
(S6) Administrative resources sharing (rooms, staff, medical records)	1. Highly	6.95	************
(S7) Mechanism of care coordination among clinic professionals	1. Formal	6.66	***********
(SA) Participation of physicians in regional committees	1. Yes	6.40	***********
(S2) Medico-administrative management	1. Administrator/manager and physician	4.35	*******
(S1) Type of governance	2. Private	3.86	******
(S5) Mode of remuneration	1. Fee-for-service	3.86	******

***Professional integrated coordination* model**			
**Wording of variables**	**Characteristic modalities**	**Test value**	**Histogram**
(S2) Medico-administrative management	2. Physician-in-charge and physician group	3.54	******
**Practices**			
(PC) Sharing of clinical activities	1. More important	8.07	*************
(PB) Role of the nurse	1. Extended role (innovator)	6.36	***********
(P3) Participation in a healthcare access network	1. Yes	6.34	**********
(P8) Number of diagnostic and therapeutic procedures available on the site of the clinic	1. Much (7-10)	6.08	**********
(PH) Maintenance and evaluation of professional competence	1. + mechanisms	6.06	**********
(P1) Availability of services in the evening and on the week-end	1. High	5.95	**********
(P5) Number of settings where the clinic's services are available (home, hospital or residential centres)	1. 2-3 settings	4.66	********
(PF) Network of reference with private institutions	2. Limited	4.08	*******
(P7) Range of services available on the site of the clinic (diagn./prevention)	1. Very extended	3.83	******
(PG) Organizational affiliation of the physicians	1. Double affiliation	3.44	******
(PA) Assistance by professionals for appointments with specialist	1. + support	3.09	*****
(P6) Approximate time for a consultation	2. Moderate	2.56	****

Class: Class 4 / 5 (Workforce: 55 - Percentage: 11.63)

***Community* model**			
**Wording of variables**	**Characteristic modalities**	**Test value**	**Histogram**
**Vision**			
(V6) Financial profitability	1. Less important	7.44	************
(V7) Valued team work	1. More important	4.01	*******
(V2) Population targeted to be served by the clinic	1. Population	3.76	******
(V1) Scope of responsibility	1. More socioenvironmental	3.69	******
(V8) Resp. towards work colleagues and clinic managers	1. More important	3.14	*****
(V3) Clinic's priority regarding service organization	1. Continuity of care	3.02	*****
(V5) Responsibility and right regarding health and access to services	1. More social	2.94	*****
**Resources**			
(R4) Financing sources for the operating costs of the clinic	1. Public source only	17.71	************** ************** *
(R2) Presence of a nurse	1. Yes	9.55	**************

Community model			
**Wording of variables**	**Characteristic modalities**	**Test value**	**Histogram**
			**
(R7) Availability of a technical platform on the site of the clinic	2. Biological specimen collection only	6.69	***********
(R1) Number of FTE MDs	1. > 6 FTE	6.44	***********
(R6) Number of information's technologies	1. 3 tech. and +	5.58	**********
(R3) Presence of other health prof. or medical specialist MDs	2. Health professional only	4.94	*********
(R5) Financial contribution from patients or enterprises	1. No	2.57	****
**Structure**			
(S1) Type of governance	1. Public	18.05	******************************
(S5) Mode of remuneration	2. Time based and various modes	18.05	******************************
(S6) Administrative resources sharing (rooms, staff, medical records)	2. Moderately	8.42	**************
(S2) Medico-administrative management	2. Physician-in-charge and physician group	8.18	**************
(S7) Mechanism of care coordination among clinic professionals	1. Formal	6.12	***********
(SA) Participation of physicians in regional committees	1. Yes	5.97	***********
**Practices**			
(PB) Role of the nurse	1. Extended role (innovator)	10.03	*****************
(PH) Maintenance and evaluation of professional competence	1. + mechanisms	8.01	*************
(P6) Approximate time for a consultation	1. Long	7.69	*************
(P7) Range of services available on the site of the clinic (diagn./prevention)	1. Very extended	6.80	***********
(PG) Organizational affiliation of the physicians	1. Double affiliation	6.51	***********
(PD) Network of reference with PHC clinics	2. Limited	5.50	*********
(P5) Number of settings where the clinic's services are available (home, hospital or residential centres)	1. 2-3 settings	4.72	********
(P5) Number of settings where the clinic's services are available (home, hospital or residential centres)	2. 1 setting	3.18	*****
(PF) Network of reference with private institutions	2. Limited	3.01	*****
(PC) Sharing of clinical activities	1. More important	2.03	***

Class: Class 5 / 5 (Workforce: 174 - Percentage: 36.79)

Professional single-provider **model**			
**Wording of variables**	**Characteristic modalities**	**Test value**	**Histogram**
**Vision**			
(V8) Resp. towards work colleagues and clinic managers	2. Less important	16.38	*************<br>*************<br>*
(V7) Valued team work	2. Less important	13.95	*************<br>**********
(V2) Population targeted to be served by the clinic	2. Clinic's regular clients	2,89	*****
**Resources**			
(R1) Number of FTE MDs	4. <= 1 FTE	20.01	*************<br>*************<br>*******
(R7) Availability of a technical platform on the site of the clinic	3. No technical platform	9.21	*************<br>**
(R2) Presence of a nurse	2. No	8.75	*************<br>*
(R4) Financing sources for the operating costs of the clinic	3. Physician source only	8.07	*************
(R3) Presence of other health prof. or medical specialist MDs	3. None	7.08	************
(R6) Number of information's technologies	4. None	5.29	*********
(R3) Presence of other health prof. or medical specialist MDs	2. Health professional only	2.34	****
**Structure**			
(S6) Administrative resources sharing (rooms, staff, medical records)	3. None	21.73	*************<br>*************<br>**********
(S2) Medico-administrative management	3. Not specified and solo practice physician	20.77	*************<br>*************<br>********
(S7) Mechanism of care coordination among clinic professionals	3. None	18.02	*************<br>*************<br>****
(SA) Participation of physicians in regional committees	2. No	7.07	************
(S1) Type of governance	2. Private	6.99	************
(S5) Mode of remuneration	1. Fee-for-service	6.99	************
(S4) Attendance time of the physician in the clinic	1. Long	5.02	********
(S3) Seniority of physicians	1. > 5 years	2.91	*****
**Practices**			
(PH) Maintenance and evaluation of professional competence	3. - mechanisms	20.02	*************<br>*************<br>*******

Professional single-provider **model**			
**Wording of variables**	**Characteristic modalities**	**Test value**	**Histogram**
(PC) Sharing of clinical activities	2. Less important	18.07	******************************
(PG) Organizational affiliation of the physicians	3. None	10.31	*****************
(PB) Role of the nurse	3. No nurse	8.75	**************
(P8) Number of diagnostic and therapeutic procedures available on the site of the clinic	3. A little or none (0-3)	8.47	**************
(PZ) Types of consultation	1. + scheduled appointments	7.09	************
(P7) Range of services available on the site of the clinic (diagn./prevention)	3. Restricted	5.51	*********
(P1) Availability of services in the evening and on the week-end	3. Low	5.15	*********
(P9) Management of chronic diseases	1. Overall responsibility	4.93	********
(P2) Possibility for regular patients of the clinic who have an urgent health problem to contact a nurse or a physician	1. High	4.60	********
(P3) Participation in a healthcare access network	2. No	3.37	******
(PG) Organizational affiliation of the physicians	2. Single affiliation	3.10	*****
(P5) Number of settings where the clinic's services are available (home, hospital or residential centres)	3. None	2.72	*****
(PD) Network of reference with PHC clinics	1. Extended	2.46	****
(P2) Possibility of regular patients of the clinic who have an urgent health problem to contact a nurse or a physician	2. Moderate	2.21	****
(PF) Network of reference with private institutions	1. Extended	2.05	***

B3 Taxonomy of contexts

A methodological report of the context analysis was produced (Roberge, Pineault et al., 2007). The report presents the methodology and results for this component of the study. Details are provided of the approach used, variables chosen and taxonomy construction process. The information that is most useful to understanding the methodology section follows.

The variables retained for the context analysis are listed in Table B4. Results of statistical analyses are presented in Figures B4 and B5. The results are given by

- Quotient = inter-class inertia (variance)/total inertia (variance) (the higher the quotient the greater is inter-class inertia).
- Total inertia = total inertia (variance) of the cloud of individuals (value is identical for all partitions),

- Inter-class inertia = inter-class variance, which measures heterogeneity among classes in the partition.
- Partition: corresponds to a number of groups of contexts. The number of groups presented rises from 2 to 10.

Finally, inter-class distances that express group differences are shown in Figure B6 and Table B5.

Table B4: Variables retained for the analysis

Dimensions and variables	Procedures and CSSS workforce by procedure	Threshold value
**Characteristics of the population (n=6)**		
1. Population aged 65 and over (%)	1. Below average (n=4)<br>2. Around average (n=15)<br>3. Above average (n=4)	1. (- ((X<11,3)<br>2. ((11,3(X(16,2)<br>3. (+ ((X>16,2)
2. Population aged 0 to 14 years (%)	1. Below average (n=3)<br>2. Around average (n=16)<br>3. Above average (n=4)	1. (- ((X<14,3)<br>2. ((14,3(X(19,8)<br>3. (+ ((X>19,8)
3. Life expectancy at birth (years)	1. Below average and median (n=4)<br>2. Around average and median (n=13)<br>3. Above average and median (n=6)	1. L.E.B. <78 years<br>2. L.E.B. =78 or 79 years<br>3. L.E.B. = 80 years and over
4. Material and social deprivation index	1. Advantaged (n=8)<br>2. Materially advantaged (n=4)<br>3. Socially advantaged (n=4)<br>4. Disadvantaged (n=7)	1. Advantaged: value of material and social indices lower than or equal to median<br>2. Materially advantaged: value of material index lower than or equal to median<br>3. Socially advantaged: value of social index lower than or equal to median<br>4. Disadvantaged: value of material and social indices higher than median
**Resources (n=4)**		
5. Availability of hospital and specialised resources	1. Low (n=5)<br>2. Average (n=13)<br>3. High (n=5)	1. Score = 4 or 5<br>2. Score = 6 or 7<br>3. Score = 8 or more
6. Availability of FTE general practitioners (Ratio of GPs per 1 000 inhabitants)	1. Low (n=8)<br>2. Average (n=7)<br>3. High (n=8)	1. X< 0,80<br>2. 0,80 ≤X ≤ 0,90<br>3. X> 0,90
7. Availability of clinics (number of clinics per km^2)	1. Low (n=10)<br>2. Average (n=6)<br>3. High (n=7)	1. X<0,22<br>2. 0,22 ≤X≤ 0,71<br>3. X>0,71

Dimensions and variables	Procedures and CSSS workforce by procedure	Threshold value
8. PDI*-RDI** for medical services provided by GPs in private practice	1. Closed market (n=12)<br>2. Mixed market (n=5)<br>3. Open market (n=6)	PDI and RDI above respective medians<br>PDI below median / RDI above median<br>PDI and RDI below respective medians
**Clinical collaborations (n=2)**		
9. Degree of collaboration among primary care medical services in a HSSC territory	1. Poor (n=19)<br>2. Strong (n=4)	1. Collaboration poor or rather poor<br>2. Collaboration strong or rather strong
10. Degree of collaboration between primary care medical services and hospitals located outside the HSSC territory	1. Poor (n=19)<br>2. Strong (n=4)	1. Collaboration poor or rather poor<br>2. Collaboration strong or rather strong

* PDI: Population dependency index
** RDI: Resource dependency index

Figure B4: Inter-class and intra-class inertia

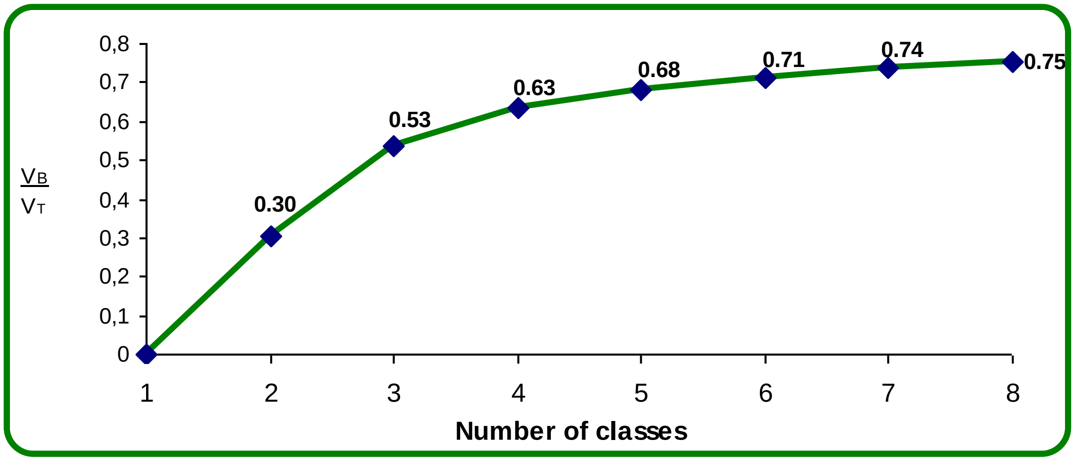

Note: The ratio $\frac{\text{Variance between}}{\text{Variance}}$

increases steadily with the first 5 partitions but levels off after that point.

Figure B5 : "Inertia quotient increase between partitions"

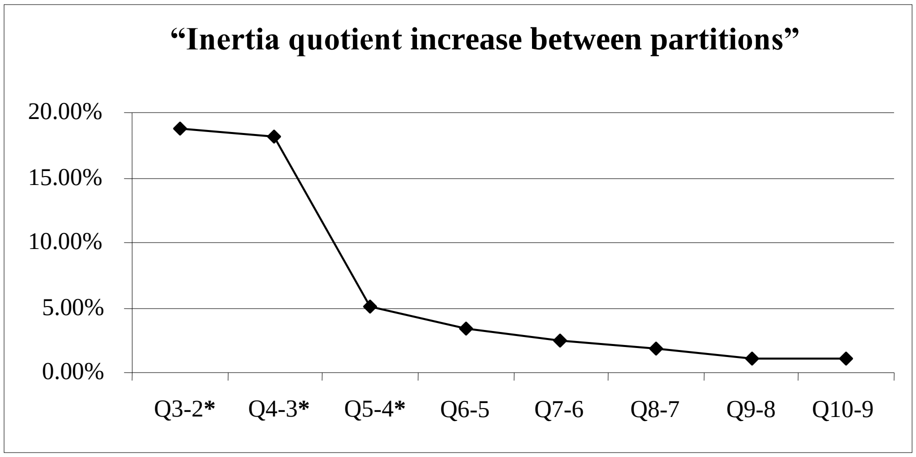

*We note that homogeneity increases from partitions 2 to 3 and 3 to 4 by about 18 points. However, from partitions 4 to 5, the increase falls to 5 points.

Figure B6: Graphic representation of the 4 groups over the first two factorial axes

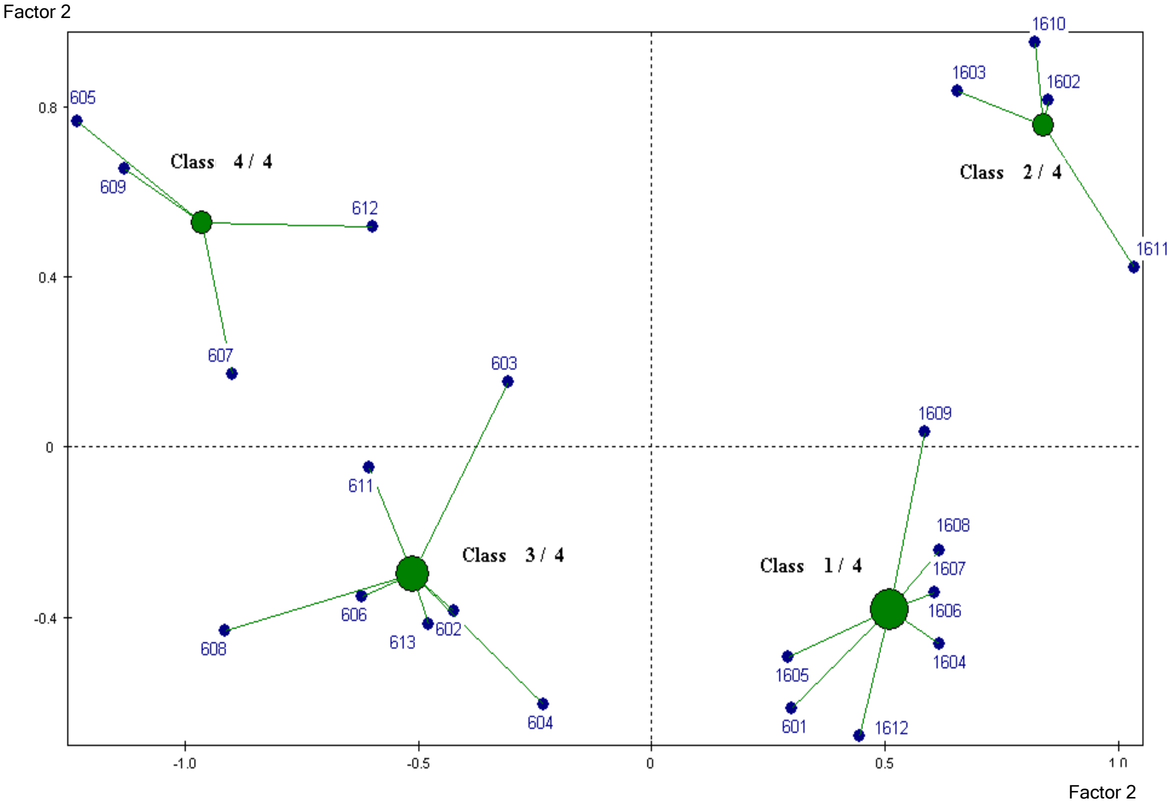

Table B5: Distance of HSSC territories from the centre of their class

Class 1/ 4 Workforce: 8

Rank	Distance from the centre of the class	Particulars
1	0.06266	1607
2	0.06266	1608
3	0.06537	1606
4	0.11312	601
5	0.30885	1605
6	0.37596	1609
7	0.39002	1604
8	0.43177	1612

Class 2 / 4 Workforce: 4

Rank	Distance from the centre of the class	Particulars
1	0.06103	1603
2	0.07609	1602
3	0.07619	1610
4	0.15449	1611

Class 3 / 4 Workforce: 7

Rank	Distance from the centre of the class	Particulars
1	0.01621	602
2	0.01757	613
3	0.15872	606
4	0.22441	604
5	0.23779	611
6	0.27802	603
7	0.44700	608

Class 4 / 4 Workforce: 4

Rank	Distance from the centre of the class	Particulars
1	0.13197	605
2	0.15065	612
3	0.25643	607
4	0.33979	609

B4 Ideal-type conformity score

These indices measure organisational profile based on 15 attributes grouped around four dimensions: vision, structure, resources, and practices. Each attribute is composed of two values (1, 0) depending on whether the organisation has or does not have this attribute. The score is expressed in percentage either for each or for all dimensions. The overall score was determined based on 15 attributes grouped together (Table B6).

Table B6: Composition of ideal-type conformity scores

**Vision index**		
**Variables**	**Codes**	**%**
Priority to continuity or accessibility	Cont = 1<br>Access = 0	79.1
Target population/clientele	Pop = 1<br>Other = 0	22.5
Common values shared by professionals about mission	Yes = 1<br>No = 0	37.2
Importance of team work	Yes = 1<br>No = 0	41.6
**Resources index**		
**Variables**	**Codes**	**%**
Number of MDs (FTE) (7 and +)	7 and + = 1<br>6 and - = 0	20.5
Innovative role of nurse	Yes = 1<br>No = 0	20.5
Information technologies (3 and +)	3 and + = 1<br>2 and - = 0	18.3
**Structure index**		
**Variables**	**Codes**	**%**
Sharing of resources (5 and +)	5 and + = 1<br>4 and - = 0	43.2
Sharing of clinical activities (4 and +)	4 and + = 1<br>3 and - = 0	51.4
Participation to an on call system or accessibility network	Yes = 1<br>No = 0	19.2
Nurse acting as liaison	Yes = 1<br>No = 0	18.0
**Practice index**		
**Variables**	**Codes**	**%**
Importance of walk-in patients (25-50% of visits)	25.5%-50% = 1<br>Other = 0	16.5
On call system outside opening hours	Yes = 1<br>No = 0	25.8
Measures for managing and follow-up of chronic diseases	Yes = 1<br>No = 0	33.2
Clinic helping patients for appointment to specialist	Yes = 1<br>No = 0	35.0

Corrected / March 2012

B5 Analyses of the relationships among organisational models, experience of care, having a family physician and unmet needs for care

Multiple regression analyses were performed for different dependent variables as part of this study. We analysed factors associated with reporting unmet healthcare needs (logistic regression), with having a regular family physician (logistic regression), and with better experience for each index of experience of care (ordinal regression). Analyses were on weighted data adjusted for design effects.

Each analysis carried out structurally in broad stages to develop multiple models. Variables that presented over 5% of missing data were excluded from the study. Variables associated with the dependent variable of interest that presented a significant bivariate association test were considered for the analyses ($p<0.20$). Variables strongly correlated with other significant variables were excluded from analyses to reduce the risk of multicollinearity.

Regression models were developed by progressive introduction of the following blocs of variables: Socioeconomic and economic status variables; health status and services utilisation variables; clinical affiliation variables; and life circumstances variables. Variables that were not significant were excluded from the final models.

For experience of care, aggregate indices (continuous variable) were assigned to three categories: 0%-69%; 70%-89%; 90% and over. These variables were modeled using ordinal regressions. Proportionality assumption was tested using ordinal models with fixed and with variable coefficients (partially proportional models). The proportionality hypothesis was respected in most models. Sensitivity analyses were performed by producing logistic regression models based on the contrasts “90% and over – less than 90%” and “70% and over – less than 70%.” All models proved to be very stable.

APPENDIX C – PRESENTATIONS AND PUBLICATIONS

Communications – partners/decision makers

Pineault R, Hamel M, Levesque JF. L'accessibilité et la continuité des services de santé : une étude sur la première ligne au Québec Quelques résultats Présentation sur invitation du MSSS du Québec, 11 février 2008.

Levesque JF, Pineault R, Roberge D, Hamel M, Lozier-Sergerie A. L'accessibilité et la continuité des services de santé» : une étude sur la première ligne dans deux régions du Québec, réunion des co-chercheurs et de l'équipe de travail, 2 novembre 2007.

Hamel M, Roberge D. L'accessibilité et la continuité des services : une étude sur la première ligne au Québec. ASSS de la Montérégie, le 14 juin 2007.

Roberge D, Larouche D, Pineault R, Levesque JF, Hamel M, Simard B. L'urgence hospitalière : un substitut à la première ligne? ASSS de la Montérégie, 14 juin 2007.

Pineault R, Levesque JF, Hamel M, Roberge D, Simard B, Foisy L, Robert L. Mesurer l'association entre les modèles d'organisation de première ligne et l'expérience de soins des populations : aspects méthodologiques. Présentation au ministère de la Santé et des Services sociaux, 3 mai 2007.

Levesque JF, Hamel M, Roberge D. Mesurer l'association entre les modèles d'organisation de première ligne et l'expérience de soins des populations : résultats préliminaires. Présentation au ministère de la Santé et des Services sociaux, 3 mai 2007.

Pineault R. Évaluer les besoins de santé : Comment s'y prendre pour ne pas s'y perdre? Présentation au Département de santé publique de l'Hôpital Charles LeMoyne, 18 avril 2007.

Levesque JF. L'accessibilité et la continuité des services de santé : Une étude sur la première ligne au Québec : L'expérience de soins de la population. Rencontre de la table locale du DRMG, CSSS Bordeaux-Cartierville-St-Laurent, 4 avril 2007.

Pineault R, Levesque JF, Hamel M, Roberge D, Prud'homme A, Perron M, Simard B, Foisy L. Les cliniques-GMF et les cliniques-réseau à Montréal et en Montérégie : Réalité et potentiel. Résultats d'une analyse secondaire des données de l'étude L'accessibilité et la continuité des services de santé : une étude sur la première ligne. Groupe de réflexion Chaire GETOS et INSPQ, Direction de santé publique de Montréal, 13 décembre 2006.

Pineault R, Levesque JF, Hamel M, Roberge D, Simard B, Foisy L, Robert L. L'accessibilité et la continuité des services de santé : une étude sur la première ligne – Résultats. Présentation au Comité consultatif, Agence de la santé et des services sociaux de Montréal, 27 novembre 2006.

Pineault R, Levesque JF, Hamel M, Roberge D, Simard B, Foisy L, Robert L. L'accessibilité et la continuité des services de santé : une étude sur la première ligne – Résultats. Agence de la santé et des services sociaux de Montréal, 31 octobre 2006.

Pineault R, Levesque JF, Hamel M, Roberge D, Simard B, Foisy L, Robert L. L'accessibilité et la continuité des services de santé : une étude sur la première ligne – Résultats. Comité de direction du département régional de médecine générale, Agence de la santé et des services sociaux de la Montérégie, 25 octobre 2006.

Pineault R. Hamel M, L'accessibilité et la continuité des services de santé : une étude sur la première ligne – Résultats. Comités de coordination stratégique de l'Agence de la santé et des services sociaux de la Montérégie, 20 octobre 2006.

Levesque JF, Pineault R. Descriptif populationnel et préliminaire organisationnel. Comité de gestion des réseaux locaux de Montréal (CGRM), 14 juin 2006.

Levesque JF, Roberge D, Pineault R, Simard B, Hamel M. Descriptif populationnel et préliminaire organisationnel. Agence de la santé et des services sociaux de Montréal, 12 juin 2006.

Levesque JF, Roberge D, Pineault R, Simard B, Hamel M, Foisy L. Descriptif populationnel et préliminaire organisationnel. Présenté à l'Agence de la santé et des services sociaux de Montérégie, 1er juin 2006.

Pineault R, Hamel M. L'accessibilité et la continuité des services de santé : une étude sur la première ligne. Agence de la santé et des services sociaux de la Montérégie, 1er juin 2006.

Pineault R, Hamel M, Foisy L. Le mode d'organisation optimal des ressources et des services en Montérégie : une réflexion critique. Quatrième colloque montérégien sur les réseaux locaux de services, Château Vaudreuil, 25 et 26 mai 2006.

Levesque JF, Roberge D. L'accessibilité et la continuité des services de santé : une étude sur la première ligne. Présentation au Comité de coordination stratégique de la Montérégie, 12 mai 2006.

Pineault R, Levesque JF, Hamel M. Projet accessibilité et continuité des services de santé : une étude sur la première ligne : Résultats préliminaires. Présentation à la Direction de santé publique de l'ASSS de Montréal et à l'Institut National de santé publique du Québec, 6 mai 2006.

Pineault R, Hamel M, Foisy L. Projet accessibilité et continuité des services de santé : une étude sur la première ligne : Résultats préliminaires. Présentation Équipe Pratiques cliniques préventives de la Direction de santé publique de l'ASSS de Montréal, 4 mai 2006.

Pineault R, Levesque JF, Roberge D, Hamel M, Foisy L. L'accessibilité et la continuité des services de santé : une étude sur la première ligne au Québec. Comité des DG de CSSS de Montérégie, 10 mars 2006.

Pineault R, Levesque JF, Roberge D, Hamel M, Foisy L. L'accessibilité et la continuité des services de santé : une étude sur la première ligne au Québec. Comité du DRMG de la Montérégie, 6 mars 2006.

Levesque JF, Roberge D, Pineault R, Simard B, Hamel M, Foisy L. Bilan des travaux et résultats préliminaires. Présentation au Comité du Directeur régional de médecine générale (DRMG) de Montréal, 6 mars 2006.

Pineault R, Levesque JF, Roberge D, Hamel M. Foisy L, L'accessibilité et la continuité des services de santé : une étude sur la première ligne au Québec. Comité des DG des CSSS de Montréal, 22 février 2006.

Levesque JF. L'accessibilité et la continuité des services de santé : une étude sur la première ligne au Québec. Présentation à la table national de concertation en santé publique du Québec. Agence de développement des réseaux locaux de services de santé et services sociaux de la Montérégie; Longueuil, 2 juin 2005.

Pineault R, Levesque JF, Roberge D, Hamel M, Foisy L. L'accessibilité et la continuité des services de santé : une étude sur la première ligne au Québec. Comité des DG des CSSS de Montréal, 15 avril 2005.

Pineault R, Levesque JF, Roberge D, Hamel M, Foisy L. L'accessibilité et la continuité des services de santé : une étude sur la première ligne au Québec. Comité des DG des CSSS de Montérégie, 6 avril 2005.

Pineault R, Levesque JF, Roberge D, Hamel M. L'accessibilité et la continuité des services de santé : une étude sur la première ligne au Québec. Comité de gestion des réseaux locaux, Agence de la santé et des services sociaux de Montréal, 16 mars 2005.

Pineault R, Levesque JF, Roberge D, Hamel M. L'accessibilité et la continuité des services de santé : une étude sur la première ligne au Québec. Comité du DRMG de l'Agence de la santé et des services sociaux de Montérégie, 9 mars 2005.

Pineault R, Levesque JF, Roberge D, Hamel M. L'accessibilité et la continuité des services de santé : une étude sur la première ligne au Québec. Comité du DRMG de l'Agence de la santé et des services sociaux de Montréal, 8 mars 2005.

Solicited scientific communications

Pineault R, Hamel M, Levesque JF. La performance des organisations de santé de première ligne : les résultats d'une étude dans deux régions du Québec. Présentation à l'occasion de la visite du Dr Marc Danzon. Institut national de santé publique. Québec, le 29 avril 2008.

Pineault R, Hamel M, Levesque JF. Séminaire organisé par l'association québécoise d'établissements de santé et de services sociaux (AQESS). Québec, 24 avril 2008.

Pineault R, Hamel M. « Une approche populationnelle pour apprécier la performance des services de santé de première ligne : les résultats d'une recherche sur les organisations de première ligne dans deux régions du Québec » présentation sur invitation du GRÉAS I et de la Direction de santé publique de l'ASSS de Montréal, 26 février 2008.

Pineault R, Levesque JF, Breton M, Lozier-Sergerie A. Évaluer la performance des différents types d'organisation de première ligne à partir des données d'une recherche réalisée dans deux régions du Québec. Un continuum de services à prescrire. Association Québécoise d'établissement de santé et de services sociaux, Montréal, Québec, 5 décembre 2007.

Pineault R. La performance des différents types d'organisation de première ligne : résultats d'une recherche dans deux régions du Québec. Colloque organisé par les médecins canadiens pour le régime public : « Solutions publiques pour améliorer l'accès aux soins de santé ». Montréal, 15 novembre 2007.

Breton M, Pineault R, Levesque JF. Évaluation de la performance des systèmes de santé. Table ronde : propriété publique, privée ou communautaire. Seizième colloque annuel de la SQÉP. Montréal, 2 novembre 2007.

Hamel M, Pineault R, Roberge D, Levesque JF. La réalisation d'une enquête sur l'organisation des services médicaux de première ligne : défis conceptuels et méthodologiques. Centre de recherche de l'hôpital Charles LeMoyne, 19 octobre 2007.

Levesque JF, Pineault R, Hamel M, Lozier-Sergerie A. Une approche populationnelle à l'évaluation de la performance des organisations de première ligne: résultats d'une étude au Québec. Mesures de performance en santé, 3e édition, Montréal, Québec, 16 octobre 2007.

Pineault R, Levesque JF, Hamel M, Roberge D, Feldman D, Ouchêne H, Simard B, Lozier-SergerieA. Une approche populationnelle à l'évaluation de la performance des organisations de première ligne : résultats d'une étude au Québec, Mesures de performance en santé. Troisième édition, Montréal, 16 octobre 2007.

Levesque JF. L'accessibilité et la continuité des services de 1re ligne en Montérégie et à Montréal : Méthodes et résultats descriptifs, Département de santé publique de l'Hôpital Charles LeMoyne, 10 octobre 2007.

Pineault R, Levesque JF, Hamel M, Lozier-Sergerie A. Évaluer la performance des organisations de première ligne : Une approche populationnelle et systémique. 6e sommet québécois sur la gestion de la performance dans la santé. Montréal 24 et 25 septembre 2007.

Pineault, R., Levesque, JF, Hamel M. Les groupes de médecine de famille et des cliniques-réseau : Potentiel et réalité. Colloque Transformer les services médicaux de première ligne : enseignement à tirer de l'expérience des premiers Groupes de médecine de famille au Québec/75e congrès de l'ACFAS, Trois-Rivières, 7 mai 2007.

Pineault R, Levesque JF, Hamel M, Roberge D, Prud'homme A, Perron M, Simard B, Foisy L, Lozier-Sergerie A. L'expérience de soins des utilisateurs de services de première ligne dans les différents modes d'organisation à Montréal et en Montérégie. Colloque de la commission des Présidents des 19 associations affiliées à la FMOQ, 12 avril 2007.

Pineault R, Breton M. Comment concilier responsabilité populationnelle et qualité des services : un défi pour les gestionnaires. Les leviers du leadership de qualité, Congrès de l'Association des directeurs généraux des services de santé et des services sociaux du Québec, Québec, 4 avril 2007.

Pineault R. Comment concilier l'intérêt des uns et des autres dans le partenariat chercheurs/ décideurs : L'exemple du collectif de recherche? Séminaire ANEIS, Université de Montréal, mercredi le 14 mars 2007.

Pineault R. La maîtrise des dépenses imposée au système de santé se fait au détriment de la qualité des services rendus. Table ronde au 2e Forum franco-québécois sur la santé « Maîtrise des dépenses de santé ou qualité : faut-il choisir? » Paris, 5 mars 2007.

Unsolicited scientific communications

Pineault R, Levesque JF, Breton M. Should government invest public funds in privately owned PHC (PHC) clinics? Conférence de l'Association canadienne pour la recherche sur les services et les politiques de la santé (ACRSPS), Gatineau, Québec, Canada, 26-28 juin 2008.

Pineault R, Levesque JF, Breton M. The performance of publicly and privately owned primary healthcare organizations. Academy Health, Poster session. Washington, 9 juin 2008.

Hamel M, Pineault R, Roberge D, Levesque JF, Lamarche P, Borgès DaSilva R. Les modèles d'organisation des services médicaux de première ligne au Québec, Société Française de Santé Publique, Montpellier 23 – 25 novembre 2007.

Levesque JF, Pineault R, Hamel M, Lemoine O, Simard B, Roberge D, Lozier-Sergerie A. La construction et la validation d'indices d'expérience de soins : la convergence des perspectives conceptuelle et statistique. Communication affichée. Société Française de Santé Publique, Montpellier 23 – 25 novembre 2007.

Roberge D, Pineault R, Hamel M, Levesque JF, Prud'homme A. L'influence des modèles d'organisation des services médicaux de première ligne sur l'expérience de soins de la population, Société Française de Santé Publique, Montpellier, 23-25 novembre 2007.

Roberge D, Borgès Da Silva R, Pineault R, Levesque JF, Hamel M, Taxonomie des contextes des modèles de santé primaire : développement et applications, Communication affichée. Société Française de Santé Publique, Montpellier, 23-25 novembre 2007.

Levesque JF, Pineault R, Lemoine O, Simard B, Haggerty J, Hamel M, Roberge D. Developing comprehensive indices of experience of primary care : triangulating statistical and conceptual validation processes. North America Primary Care Reseach Group Annual Meeting, Vancouver, BC, 22 octobre 2007.

Pineault R, Hamel M, Levesque JF, Roberge D, Lamarche P, Borgès Da Silva R. A taxonomy of primary healthcare organizations in Quebec. North America Primary Care Research Group Annual Meeting, Vancouver, BC, 22 octobre 2007.

Pineault R, Levesque JF, Hamel M, Prud'homme A. The potential of emerging Family Medicine Groups (FMG) and network clinics in Québec. Conférence de l'association canadienne pour la recherché sur les services et les politiques de santé (ACRSPS), Toronto Ontario, 12 - 14 juin 2007.

Levesque JF, Pineault R, Kapetanakis C. Primary care affiliation and unmet needs for care for vulnerable populations; results from a population-based survey in Quebec province. Conférence de l'association canadienne pour la recherché sur les services et les politiques de santé (ACRSPS), Toronto Ontario, 12 - 14 juin 2007.

Roberge D, Larouche D, Pineault R, Perron M, Levesque JF, Hamel M, Simard B. Why do people choose the hospital emergency instead of their regular source of primary care? Communication affichée. 34th NAPCRG Annual Meeting, Tucson, Arizona, 15-18 octobre 2006.

Larouche D, Roberge D, Pineault R, Levesque JF. Démarches préalables et issues de la visite à l'urgence hospitalière : résultats d'une enquête québécoise auprès de la population. XVII Congrès de l'ALASS, Milan, 6 octobre 2006.

Larouche D, Roberge D, Pineault R, Levesque JF. Pourquoi la population québécoise choisit-elle de recourir à l'urgence? XVII Congrès de l'ALASS 2006 Milan, 5-7 octobre 2006.

Pineault R. Mise en contexte et description de la recherche. La conduite d'une recherche appliquée dans un contexte de restructuration des services de première ligne : Défis et potentiel d'application. XVII Congrès de l'ALASS, Milan, 5-7 octobre 2006.

Pineault R, Levesque JF, Simard B, Hamel M, Roberge D. Quelles sont les pratiques d'utilisation des services de 1re ligne par la population et que pensent les utilisateurs des services reçus? XVII Congrès de l'ALASS, Milan, 5-7 octobre 2006.

Pineault R, Levesque JF, Hamel M, Simard B, Roberge D, Foisy L. Le jumelage de données d'enquêtes populationnelle et organisationnelle : défis et potentiels. XVII Congrès de l'ALASS, Milan, 5-7 octobre 2006. Communication affichée.

Roberge D, Hamel M, Pineault R, Simard B, Borges Da Silva R, Levesque JF, Robert L, Foisy L. La conduite d'une recherche appliquée dans un contexte de restructuration des services de première ligne : Défis et potentiel d'application. XVII Congrès de l'ALASS, Milan, 5-7 octobre 2006.

Roberge D, Hamel M, Borges Da Silva R, Pineault R, Levesque JF. Comment sont structurées et organisées les cliniques médicales de la 1re ligne. XVII Congrès de l'ALASS, Milan, 5-7 octobre 2006.

Simard B, Pineault R, Levesque JF, Hamel M, Roberge D, Foisy L. Le raccordement de données d'enquêtes populationnelle et organisationnelle : défis et potentiel. Communication affichée. XVII Congrès de l'ALASS, Milan, 7 octobre 2006.

Levesque JF, Pineault R, Robert L, Simard B, Laugraud A. Intra-regional variations in coverage of primary healthcare services : Informing decision-making through a population survey. CHSRF Primary Healthcare Network Symposium, Vancouver, 20 septembre 2006.

Larouche D, Roberge D, Pineault R, Levesque JF. Emergency room utilization in the population : Highlighting barriers to care and unmet needs for services in Québec. CHSRF Primary Healthcare Network Symposium, Vancouver, 20 septembre 2006.

Levesque JF, Pineault R, Hamel M, Simard B, Roberge D, Foisy L. Challenges in linking population and organization data to inform policy : Evidence from a Quebec study. 2006 Annual CAHSPR Conference, Vancouver, 17-19 septembre 2006 (communication affichée).

Reports, pamphlets and thematic reports

Hamel M, Pineault R, Levesque JF, Roberge D, Lozier-Sergerie A, Prud'homme A, Simard B. L'organisation des services de santé de première ligne : Portrait des services médicaux de première ligne à Montréal et en Montérégie. Institut national de santé publique du Québec, Direction de santé publique de l'Agence de la santé et des services sociaux de Montréal et Centre de recherche de l'hôpital Charles LeMoyne, décembre 2007. Rapport et résumé.

Hamel M, Pineault R, Levesque JF, Roberge D, Lozier-Sergerie A, Prud'homme A, Simard B. A portrait of primary care medical services in Montréal and Montérégie. Institut national de santé publique du Québec, Direction de santé publique de l'Agence de la santé et des services sociaux de Montréal et Centre de recherche de l'hôpital Charles LeMoyne, décembre 2007. Summary.

Roberge D, Pineault R, Hamel M, Da Silva R, Cazale L, Levesque JF, Ouellet D. Rapport méthodologique de l'analyse des contextes. L'accessibilité et la continuité des services de santé : Une étude sur la première ligne au Québec. Centre de recherche de l'hôpital Charles LeMoyne, Direction de santé publique de l'Agence de la santé et des services sociaux de Montréal et Institut national de santé publique du Québec, décembre 2007.

Levesque JF, Pineault R, Robert L, Hamel M, Roberge D, Kapetanakis C, Simard B, Laugraud A. Unmet needs for medical services : A reflection of the accessibility of primary care services? Institut national de santé publique du Québec et Direction de santé publique de l’Agence de la santé et des services sociaux de Montréal, 2007.

Levesque JF, Pineault R, Robert L, Hamel M, Roberge D, Kapetanakis C, Simard B, Laugraud A. Les besoins non comblés de services médicaux : un reflet de l’accessibilité des services de première ligne? Institut national de santé publique du Québec et Direction de santé publique de l’Agence de la santé et des services sociaux de Montréal, 2007. cahier et résumé.

Levesque JF, Pineault R, Simard B, Roberge D, Hamel M, Kapetanakis C, Robert L. The population’s experience of care : Portrait of intra-regional variations in Montréal and Montérégie. Institut national de santé publique du Québec, Direction de santé publique de l’Agence de la santé et des services sociaux de Montréal et Centre de recherche de l’hôpital Charles LeMoyne. mars 2007. Summary.

Levesque JF, Pineault R, Simard B, Roberge D, Hamel M, Kapetanakis C, Robert L. L’expérience de soins de la population. Portrait des variations intra-régionales à Montréal et en Montérégie. Institut national de santé publique du Québec, Direction de santé publique de l’Agence de la santé et des services sociaux de Montréal et Centre de recherche de l’hôpital Charles LeMoyne, mars 2007. Rapport et résumé.

Roberge D, Larouche D, Pineault R, Levesque JF, Hamel M, Simard B. L’urgence hospitalière : un substitut à la première ligne? Résultats d’une enquête auprès de la population de Montréal et de la Montérégie. Direction de santé publique de l’Agence de la santé et des services sociaux de Montréal, Institut national de santé publique du Québec et Centre de recherche de l’Hôpital Charles LeMoyne, mars 2007. Rapport et résumé.

Roberge D, Larouche D, Pineault R, Levesque JF, Hamel M, Simard B. Hospital emergency departments : Substitutes for primary care? Results of a survey among the population of Montréal and Montérégie. Direction de santé publique de l’Agence de la santé et des services sociaux de Montréal, Institut national de santé publique du Québec et Centre de recherche de l’Hôpital Charles LeMoyne, mars 2007. Résumé.

Levesque JF, Pineault R, Simard B, Hamel M, Roberge D, Ouellet D, Haggerty J. Questionnaire populationnel. L’accessibilité et la continuité des services de santé : Une étude sur la première ligne au Québec. Institut national de santé publique du Québec, Direction de santé publique de l’Agence de la santé et des services sociaux de Montréal et Centre de recherche de l’hôpital Charles LeMoyne, avril 2006.

Levesque JF, Pineault R, Simard B, Hamel M, Roberge D, Ouellet D, Haggerty J. (2006), Population questionnaire Accessibility and continuity of health services : A study on primary carre in Québec. Institut national de santé publique du Québec, Agence de la santé et des services sociaux de Montréal et Centre de recherche de l’hôpital Charles LeMoyne, avril 2006.

Pineault R, Foisy L, Hamel M, Simard B, Levesque JF, Roberge D. Répertoire des cliniques médicales de première ligne de Montréal et de la Montérégie. Institut national de santé publique du Québec, Direction de santé publique de l’Agence de la santé et des services sociaux de Montréal et Centre de recherche de l’hôpital Charles LeMoyne, 2006.

Pineault R, Hamel M, Levesque JF, Roberge D, Lamarche P, Haggerty J. Questionnaire organisationnel Clinique médicale de première ligne. L’accessibilité et la continuité des services de santé : Une étude sur la première ligne au Québec. Institut national de santé publique du Québec, Direction de santé publique

de l'Agence de la santé et des services sociaux de Montréal et Centre de recherche de l'hôpital Charles LeMoyne, avril 2006.

Pineault R, Hamel M, Levesque JF, Roberge D, Lamarche P, Haggerty J. Organisational questionnaire Primary healthcare clinic A study on primary care in Québec. Institut national de santé publique du Québec, Agence de la santé et des services sociaux de Montréal et Centre de recherche de l'hôpital Charles LeMoyne, avril 2006.

Book chapters

Lévesque JF, Roberge, D, Pineault R. La première ligne de soins : un témoin distant des réformes institutionnelles et hospitalières au Québec? Chapitre 5 dans Le système sociosanitaire au Québec : gouverne, régulation et participation. M-J Fleury, M Tremblay, H. Nguyen, L Bordeleau, eds. Gaëtan Morin éditeur, Chenelière Éducation, 2007, p. 63-78.

Pineault R, Levesque JF, Breton M, Hamel M, Roberge D. Réalisations et potentiel des organisations publiques et privées de première ligne dans deux régions du Québec. Dans Béland F, Contandriopoulos A-P, Quesnel-Vallée A, Robert L. Les Presses de l'Université de Montréal « Le privé dans la santé : un débat sans fin ? » (à paraître 2008).

Articles

Levesque JF, Pineault R, Kapetanakis C, Hamel M, Roberge D, Robert L, Simard B, Primary care affiliation and unmet needs for healthcare services for vulnerable population-based survey in Quebec province. Soumis pour publication, 2008.

Pineault R, Levesque JF, Hamel M, Roberge D, Prud'Homme A, Perron M, Simard B, Foisy L. Le profil et le type d'organisation de première ligne ont-ils un lien dans l'expérience de soins de leur clientèle? Résultats d'une étude dans deux régions du Québec. Infolettre, février 2007, 4 (1):2-6.

Pineault R, Levesque JF, Hamel M, Roberge D, Prud'Homme A, Perron M, Simard B, Foisy L. Are the profile and type of organization of primary care linked to experience of care for their clients? Findings of a study in two regions of Quebec, Infoletter, February 2007, 4 (1):2-6.

Pineault R, Levesque JF. Le type d'organisation des soins de première ligne a-t-il un effet sur l'expérience de soins rapportée par les utilisateurs? Le Point en administration de la santé et des services sociaux, Hiver 2007 – 2008, 3(4): 12-14.

Public at-large

Crépeau C. Soins de première ligne : Les cliniques solos sont performantes, mais les GMF forment la meilleure organisation. Actualité médicale, 22 août 2007.

Murchinson N. Impossible de voir un médecin. Journal de Montréal, 18 février 2008. http://www2.canoe.com/cgi-bin/imprimer.cgi?id=344958.

La Presse Canadienne - Le fil radio. Les consultations sans rendez-vous prennent une très grande place à Montréal. Actualités du Québec, vendredi, 15 février 2008.
