# Supplementary material for: Constructing Taxonomies to Identify Distinctive Forms of Primary Healthcare Organizations
Source: ISRN Family Med. 2013 Apr 15;2013:798347. doi: 10.5402/2013/798347 (PMC4041222; doi:10.5402/2013/798347)
Supplement: Supplementary file 1 — Research report (46) and Organizational questionnaire developed for mail organizational survey of general practitioners' PHC practice settings [file 798347.f1.zip › Supplementary Material/Add_file_2_Organizational_questionnaire_ISRNFM_477959.pdf]

Accessibility and continuity of health services:
A study on primary care in Québec

Organisational questionnaire
Primary healthcare clinic

April 2006

Institut national de santé publique du Québec
Direction de santé publique, Agence de la santé et des services sociaux de Montréal
Centre de recherche de l'hôpital Charles LeMoyne

Québec

AUTHORS

Raynald Pineault[1,2,3]
Marjolaine Hamel[1,2]
Jean-Frédéric Levesque[1,2,3]
Danièle Roberge[4,5]
Paul Lamarche[6]
Jeannie Haggerty[5]

TECHNICAL SUPPORT, RESEARCH

Léonarda Da Silva[1,2]

COOPERATION

The team of researchers and partners associated with the research project
Physicians from the DRMG/ Montréal-Montérégie
Committee members from the Départements régionaux de médecine générale de Montréal et de la Montérégie

INSTITUTIONAL SUPPORT

The research project benefits from an operational grant from the Canadian Institutes for Health Research (CIHR, the Canadian Health Services Research Foundation (CHSRF) and the Fonds de la Recherche en Santé du Québec (FRSQ). The project also benefits from financial support from the Agences de la santé et des services sociaux (ASSS) of Montréal and Montérégie, the Institut national de santé publique du Québec (INSPQ), from the Groupe de recherche sur l'équité d'accès et l'organisation des services de santé de 1re ligne (Gréas 1) and the Groupe interuniversitaire de recherche sur les urgences (GIRU).

This project has received an ethical approval from the Comité d'éthique de la recherche de la Direction de santé publique, Agence de la santé et des services sociaux de Montréal.

This document is available in electronic format on the web site of the Direction de santé publique de Montréal (www.santepub-mtl.qc.ca/ESPSS/production.html)

PRINTING AND DISTRIBUTION

Direction de santé publique, Agence de la santé et des services sociaux de Montréal

Reproduction authorised for non commercial purposes conditional to appropriate citation.

© Direction de santé publique, Agence de la santé et des services sociaux de Montréal (2006)

1 Institut national de santé publique du Québec (INSPQ)
2 Direction de santé publique, Agence de la santé et des services sociaux de Montréal
3 Centre de recherche du Centre hospitalier de l'Université de Montréal
4 Centre de recherche de l'Hôpital Charles LeMoyne
5 Université de Sherbrooke
6 Université de Montréal

QUESTIONNAIRE NUMBER |____|____|____|

L'accessibilité et la continuité des services de santé

Une étude sur la première ligne au Québec

ORGANISATIONAL QUESTIONNAIRE

PRIMARY HEALTHCARE CLINIC

1. Address of the clinic

Please make any necessary corrections.

Name of the clinic		
Number	Street	
Office number	City	
Province	Postal code	
Telephone	Extension	Fax

2. Identification of the physician answering the questionnaire ***(if different from the name and address on the label).***

Last name: ………………………………………………………………………………………..

First name: ……………………………………………………………………………………….

Email address: ………………………………………………………………………………………

Position:

❑ 1 Team leader/Medical director

❑ 2 Head Doctor/Physician-in-charge

❑ 3 Member of the team of general practitioners

❑ 95 Other ➔ ***Specify:*** ……………………………………………………………………………………………………

INSTRUCTIONS

This is a questionnaire about the **organisation of primary healthcare services** offered in medical clinics, physician's offices, polyclinics and local community services centres (CLSC). There is one questionnaire per clinic or civic address.

WHO SHOULD ANSWER THIS QUESTIONNAIRE?

The questionnaire must be filled in by the person who is most familiar with how the clinic is organised and operates; typically this is the physician-in-charge. For this reason, the questionnaire should not be completed by each physician at the clinic even when, in some clinics, physicians rarely work together.

Use of the expression "**solo practice physician**" refers to a situation where only one physician works at the clinic; this physician must then answer the questionnaire.

HOW SHOULD THE QUESTIONNAIRE BE COMPLETED?

Use of the expression "**your clinic**" refers strictly to the primary healthcare medical team (general practitioners and nurses) to which you belong, or to yourself, if you are the only physician at the clinic. Answers should reflect as much as possible the views and practices of the entire primary healthcare medical team (general practitioners and nurses).

Circle or check off ONE answer per question, unless otherwise indicated.

Some questions are only partly applicable to "**solo practice physicians**". These questions should be answered as per the instructions provided.

QUESTIONNAIRE COMPONENTS

Section A: Resources and organisational structure
Section B: Services, practices and interorganisational collaboration
Section C: Vision/Mission and value system
Section D: Clinic location

Si vous préférez recevoir ce questionnaire en français, veuillez contacter:

This is a questionnaire about the organisation of primary healthcare services offered by your medical team (physician(s) or nurse(s)), or offered by you, if you are a solo practice physician. The expression "your clinic" refers to this team or to yourself.

Section A: Resources and organisational structure

1. **a) In all, how many general practitioners work at your clinic?** ……………………………….

   b) How many general practitioners work at your clinic in the following categories.

   a) Less than 10 hours **a week:** ………………………………..
   b) 10 to 25 hours **a week:** ……………………………………
   c) 26 to 40 hours **a week:** ……………………………………
   d) More than 40 hours **a week:** ………………………………..

   c) How many of these general practitioners have been working at your clinic for more than 5 years?

   ❑ 1 None
   ❑ 2 One
   ❑ 3 Some
   ❑ 4 All or most

   d) How many nurses work at your clinic? ………………………….

2. **On the whole, how are general practitioners paid for activities conducted at your clinic?** ***Choose one answer only.***

   ❑ 1 Fee-for-service
   ❑ 2 Sessional fees (Vacation)
   ❑ 3 Fixed honorarium
   ❑ 4 Various modes of payment

☞ **Note: If you are a "solo practice physician" ➡ Go to question 6.**

3. **At your clinic, who …** ***Circle only one answer per statement.***

	A physician-in-charge or designated physician	The physician group collectively	Administrator/ manager	Does not apply
a) sets up on-call lists, schedules, vacations, etc.?	1	2	4	97
b) organises meetings for case discussions?	1	2	4	97
c) looks after recruitment of physicians and assigns practice privileges?	1	2	4	97
d) ensures that the quality of medical acts is evaluated?	1	2	4	97
e) organises continuing medical education activities?	1	2	4	97
f) represents the clinic on committees?	1	2	4	97

4. How many general practitioners at your clinic share …

	All/Most	Some	None
a) rooms *(offices or examination rooms)?*	3	2	1
b) operating costs for the clinic?	3	2	1
c) support staff *(secretary and receptionist)?*	3	2	1
d) an appointment management system?	3	2	1
e) a medical records system?	3	2	1
f) pooled income?	3	2	1

5. How many general practitioners at your clinic share …

	All/Most	Some	None
a) coverage of **walk-in** clinic periods?	3	2	1
b) coverage of scheduled **appointments periods?**	3	2	1
c) in-hospital care for the clinic's patients?	3	2	1
d) patient follow-up?	3	2	1
e) replacement for a physician absent from the clinic?	3	2	1

6. To what extent do general practitioners at your clinic feel accountable to…

	Highly	Fairly	Slightly	Not at all
a) the population of the neighbourhood, village or territory where the clinic is located?	4	3	2	1
b) the clinic's regular clients?	4	3	2	1
c) the RAMQ?	4	3	2	1
d) the Collège des médecins?	4	3	2	1
e) colleagues at work?	4	3	2	1
f) clinic managers?	4	3	2	1

7. Does the financing for the operating costs of your clinic come from …

a) fees charged to physicians or contributions by physicians? ❒ 1 Yes ❒ 2 No
b) private enterprises *(companies, pharmacies, donations, foundations, etc.)?* ❒ 1 Yes ❒ 2 No
c) fees charged to patients *(e.g. fees to open or manage files)?* ❒ 1 Yes ❒ 2 No
d) an institutional operating budget *(CLSC, hospital, etc.)?* ❒ 1 Yes ❒ 2 No

8. Do you have at your clinic…

a) computer software to manage appointments? ❒ 1 Yes ❒ 2 No
b) electronic medical records? ❒ 1 Yes ❒ 2 No
c) Internet access (Web) for physicians? ❒ 1 Yes ❒ 2 No
d) access to the health and social service telecommunications network *(RTSS)?* ❒ 1 Yes ❒ 2 No
e) an appointment system for patients on the Internet (Web)? ❒ 1 Yes ❒ 2 No
f) an electronic system to transmit prescriptions to pharmacies? ❒ 1 Yes ❒ 2 No

9. **At your clinic, where do you send patients for the following services: *Circle all that apply.***

	On site	To another primary healthcare clinic	To a CLSC	To a private laboratory/Private radiology clinic	To a hospital
a) Biological specimen collection	1	2	3	4	5
b) Radiology	1	2	3	4	5
c) Laboratory analyses	1	2	3	4	5

Section B: Services, practices and interorganisational collaboration

1. **At your clinic…**

a) do you have a reception desk for patients? ❑ 1 Yes ❑ 2 No
b) do you have a medical records manager *(opening new files, managing archives, etc.)?* ❑ 1 Yes ❑ 2 No
c) can a patient leave a message on an answering machine and get a return call from a physician or nurse? ❑ 1 Yes ❑ 2 No
d) do you offer services **by appointment** during weekends *(Saturday or Sunday)?* ❑ 1 Yes ❑ 2 No
e) do you offer **walk-in** services during weekends *(Saturday or Sunday)?* ❑ 1 Yes ❑ 2 No
f) do you offer services **by appointment** during weekday evenings *(after 6:00 p.m.)?* ❑ 1 Yes ❑ 2 No
g) do you offer **walk-in** services during weekday evenings *(after 6:00 p.m.)?* ❑ 1 Yes ❑ 2 No
h) do you offer services at night *(between midnight and 8:00 a.m.)?* ❑ 1 Yes ❑ 2 No
i) outside the clinic's opening hours, do you direct patients to another available clinic? ❑ 1 Yes ❑ 2 No
j) do you have a policy for taking **new patients?** ❑ 1 Yes ❑ 2 No
k) do you have a waiting list for **new patients?** ❑ 1 Yes ❑ 2 No

2. **Among all consultations at the clinic, what is the percentage for walk-in clinics?**

❑ 1 0%
❑ 2 1% to 25%
❑ 3 26% to 50%
❑ 4 51% to 75%
❑ 5 76% to 100%

3. **When your clinic is closed, is there an on-call system for…**

a) vulnerable patients? ❑ 1 Yes ❑ 2 No
b) regular patients of the clinic? ❑ 1 Yes ❑ 2 No
c) people who have a medical record at the clinic? ❑ 1 Yes ❑ 2 No
d) people who do not have a medical record at the clinic? ❑ 1 Yes ❑ 2 No

4. **For each client group specified below, indicate how often patients would be able to contact a physician or nurse by telephone should they get sick during the clinic's opening hours.**

	Always	Often	Sometimes	Never
a) Vulnerable patients	4	3	2	1
b) Regular patients of the clinic	4	3	2	1
c) People who have a medical record at the clinic	4	3	2	1
d) People who don't have a medical record at the clinic	4	3	2	1

5. **For each of the client groups specified below, indicate how often patients with acute health problems could see a physician during periods reserved for acute problem.**

	Always	Often	Sometimes	Never
a) Vulnerable patients	4	3	2	1
b) Regular patients of the clinic	4	3	2	1
c) People who have a medical record at the clinic	4	3	2	1
d) People who don't have a medical record at the clinic	4	3	2	1

6. **At your clinic, when a patient has a problem, can he or she be seen between scheduled appointments?**

- ❑ 4 Always
- ❑ 3 Often
- ❑ 2 Sometimes
- ❑ 1 Never

7. **At your clinic, what is the average waiting time *(in weeks)* to see a doctor by appointment?** ____________________

8. **Does your clinic confirm appointments with patients a few days before a scheduled visit?**

- ❑ 4 Always
- ❑ 3 Often
- ❑ 2 Sometimes
- ❑ 1 Never

9. **At your clinic, how much time is scheduled for visits for a complete examination or evaluation of a new patient? *Check one only.***

- ❑ 1 Less than 10 minutes
- ❑ 2 10 minutes
- ❑ 3 15 minutes
- ❑ 4 20 minutes
- ❑ 5 30 minutes
- ❑ 6 Over 30 minutes

10. **At your clinic, how much time is scheduled for follow-up visits or emergency consultations? *Check one only.***

- ❑ 1 Less than 10 minutes
- ❑ 2 10 minutes
- ❑ 3 15 minutes
- ❑ 4 20 minutes
- ❑ 5 30 minutes
- ❑ 6 Over 30 minutes

11. **At your clinic, to what extent are the following services available:**

	Very	Moderately	A little	Not at all
a) Medical or nursing care and services at a patient's home	4	3	2	1
b) Medical or nursing care and services in homes for seniors, nursing homes, residential centres	4	3	2	1
c) Palliative care	4	3	2	1
d) Hospital care	4	3	2	1
e) Obstetrical care	4	3	2	1

12. At your clinic, do you offer specific case management and systematic approaches to care for people with chronic diseases *(COPD, diabetes, etc.)?*

- ❑ 4 Always
- ❑ 3 Often
- ❑ 2 Sometimes
- ❑ 1 Never

13. Are the following diagnostic and therapeutic procedures available on-site?

a) ECG interpretation	❑ 1 Yes	❑ 2 No
b) Capillary glycaemia and capillary cholesterol	❑ 1 Yes	❑ 2 No
c) Needle aspiration	❑ 1 Yes	❑ 2 No
d) Strep test	❑ 1 Yes	❑ 2 No
e) Skin biopsy	❑ 1 Yes	❑ 2 No
f) Interpretation of simple radiological films	❑ 1 Yes	❑ 2 No
g) IUD insertion	❑ 1 Yes	❑ 2 No
h) Musculo-skeletal injection/Aspiration	❑ 1 Yes	❑ 2 No
i) Casting/Splinting	❑ 1 Yes	❑ 2 No
j) Suture/Minor surgery	❑ 1 Yes	❑ 2 No
k) Spirometry	❑ 1 Yes	❑ 2 No
l) Alternative medicine *(acupuncture, chiropractic, osteopathy, etc.)*	❑ 1 Yes	❑ 2 No

14. Are the following preventive services part of your clinic's usual clinical practices?

Screening

a) Identification of drug and alcohol problems	❑ 1 Yes	❑ 2 No
b) Screening for hypercholesterolemia	❑ 1 Yes	❑ 2 No
c) Clinical breast examinations	❑ 1 Yes	❑ 2 No
d) Mammography for women	❑ 1 Yes	❑ 2 No
e) Cervical smear *(Pap test)*	❑ 1 Yes	❑ 2 No
f) Postnatal screening	❑ 1 Yes	❑ 2 No
g) GAIAC/Coloscopy/Colonoscopy	❑ 1 Yes	❑ 2 No
h) Osteoporosis	❑ 1 Yes	❑ 2 No

Prevention

i) Childhood vaccination	❑ 1 Yes	❑ 2 No
j) Influenza vaccination	❑ 1 Yes	❑ 2 No

Counselling, education about

k) Smoking cessation	❑ 1 Yes	❑ 2 No
l) Maintaining a healthy weight	❑ 1 Yes	❑ 2 No
m) Safe-sex practices	❑ 1 Yes	❑ 2 No
n) Prevention of falls in the elderly	❑ 1 Yes	❑ 2 No

15. Are pamphlets or written information on the following topics readily available to patients at your clinic:

a) Ways to stay healthy and prevent diseases	❑ 1 Yes	❑ 2 No
b) Ways of treating and controlling chronic diseases	❑ 1 Yes	❑ 2 No

16. At your clinic, services are offered in which language(s)? *Check all that apply.*

- ❑ 1 French
- ❑ 2 English
- ❑ 95 Other languages ➔ *Specify:* ______________________________

17. At your clinic, how many of the general practitioners concentrate their clinical activities in the following specific areas: *If you are a "solo practice physician" circle your practice concentration(s) in the last column.*

	All/Most	Some	None	Solo practice physician
a) Women's health and obstetrics	4	3	2	96
b) Mental health	4	3	2	96
c) Geriatrics	4	3	2	96
d) Child and adolescent care	4	3	2	96
e) Minor surgery	4	3	2	96
f) Plastic surgery and treatment of varicose veins	4	3	2	96
g) Obesity	4	3	2	96
h) Chronic diseases *(diabetes, COPD, etc.)*	4	3	2	96
i) Industrial medicine	4	3	2	96
j) Sports medicine	4	3	2	96
k) Traveller's health	4	3	2	96
l) Prevention, counselling and screening	4	3	2	96
m) Alternative medicine *(acupuncture, chiropractic, osteopathy, etc.)*	4	3	2	96
n) Others ➔ ***Specify:*** ⬇	4	3	2	96
……				

18. In addition to practicing at your clinic, how many of the general practitioners also provide care in the following other health settings or programs: *If you are a "solo practice physician" circle the additional services in the last column.*

	All/Most	Some	None	Solo practice physician
a) In the **emergency room** of a general and/or specialized care hospital or other health institution?	4	3	2	96
b) In a **short-term care unit** of a general and specialized care hospital?	4	3	2	96
c) In a **long-term care facility** *(CHSLD)?*	4	3	2	96
d) In a CLSC **home care** programme?	4	3	2	96
e) **Obstetrical** services in a hospital?	4	3	2	96
f) Primary healthcare medical services for **vulnerable patients**, at home, in a private office or in any centre operated by a health care institution?	4	3	2	96
g) Other activities identified by the Département régional de médecine générale *(DRMG)?*	4	3	2	96

19. How many of the general practitioners at your clinic engage in activities in these other health settings: *If you are a "solo practice physician", circle the other applicable settings.*

	All/Most	Some	None	Solo practice physician
a) Private medical clinics	3	2	1	96
b) Local community services centre *(CLSC)*	3	2	1	96

20. What are the roles and functions of the nurses on your medical team? *Check all that apply.*

- ❑ a There is no nurse on our team ➔ ***Go to the next question***
- ❑ b Triage of walk-in clinic patients
- ❑ c Prevention, promotion and screening activities
- ❑ d Health education
- ❑ e Systematic approaches to care (application of care protocols)
- ❑ f Liaison and coordination with CLSCs, LTCFs, hospitals and other clinics
- ❑ g Support for medical activities *(blood pressure, weight, injections, etc.)*
- ❑ h Participation in clinical decisions
- ❑ i Others ➔ ***Specify:*** ______________________________

____

☞ **Note: If you are a "solo practice physician" ➡ Go to question 22.**

21. How is care coordinated among clinic professionals?

	Always	Often	Sometimes	Never
a) Informal or ad hoc exchanges	4	3	2	1
b) Pre-established care protocols for specific client groups or problems	4	3	2	1
c) Case discussion meetings *(statutory meetings*)	4	3	2	1
d) Continuing medical education sessions	4	3	2	1

Interorganisational collaboration

22. Where are patients sent when they need to see specialists?

	Always	Often	Sometimes	Never
a) To specialists' private offices	4	3	2	1
b) To hospitals or hospital out-patient clinics	4	3	2	1
c) To hospital emergency rooms	4	3	2	1

23. In general, appointments with specialists are made by …

- ❑ 1 The patient
- ❑ 2 The clinic support staff
- ❑ 3 The clinic nurse
- ❑ 4 The physician

24. At your clinic, are patients referred to service networks designed specifically for particular patient groups?

- ❑ 1 Yes
- ❑ 2 No ➔ ***Go to question 26***

25. What are these different service networks? *Check all that apply.*

- ❑ a Integrated service network for the elderly
- ❑ b COPD network
- ❑ c Integrated service network for people with mental health problems
- ❑ d Asthma network
- ❑ e Youth services network
- ❑ f Diabetes services network
- ❑ g Cancer services network
- ❑ h Cardiovascular diseases services network
- ❑ i Others ➔ ***Specify:*** ____________________

26. Indicate whether your clinic has formal or informal arrangements with other healthcare institutions for the following activities *Check all that apply.*

	Yes, with one or several primary healthcare medical clinic(s)	Yes, with one or several CLSC(s)	Yes, with one or several hospital(s)	NO
a) Planning services offered *(on-call activities, clinic office hours, walk-in services, etc.)?*	1	2	3	4
b) Access to technical services *(e.g. radiology, laboratory)?*	1	2	3	4
c) Exchange of resources *(e.g. loan of professionals)?*	1	2	3	4
d) Referral or transfer of patients to general practitioners, specialists or other professionals?	1	2	3	4
e) Follow-up for hospitalised patients or patients seen at the clinic?	1	2	3	4
f) Others ➔ ***Specify:*** ⬇	1	2	3	4
…………………………………………………………………………………………				

27. If you have answered "yes" to any of the choices in the preceding question, identify…

a) the main primary healthcare clinic or clinics with which you have arrangements:

____

____

____

b) the main CLSC or CLSCs with which you have arrangements:

____

____

____

c) the main hospital or hospitals with which you have arrangements:

____

____

____

28. Does your clinic participate in a healthcare access network to ensure that your clinic's office hours are coordinated with those of other clinics *(evenings, weekends, etc.)?*

- ❑ 1 Yes
- ❑ 2 No

29. Do the general practitioners at your clinic participate in a regional on-call system for vulnerable patients?

- ❑ 1 Yes
- ❑ 2 No

30. How many of the general practitioners at your clinic participate in the following activities: *If you are a "solo practice physician", circle your answer in the last column.*

	All/Most	Some	None	Solo practice physician
a) Local committees of the *Direction régionale de médecine générale (DRMG)*	3	2	1	96
b) Committees for the implementation of Family Medicine Groups *(GMF)*	3	2	1	96
c) Committees to alleviate congestion in emergency departments	3	2	1	96
d) Committees on the health and social service centre's *(CSSS)* clinical project	3	2	1	96
e) Others ➔ ***Specify:*** ⬇	3	2	1	96
....				

Section C: Vision, mission and value system

1. Which statement best represents the population that your clinic tries to serve? *Check one only.*

- ❑ 1 Anyone who needs services and shows up at the clinic.
- ❑ 2 Regular clinic patients or patients registered at the clinic.
- ❑ 3 The population in the neighbourhood, village or territory served by the clinic.

2. Does your clinic's mission include offering services to specific patient groups (*e.g. children, adolescents, elderly*)?

- ❑ 1 Yes ➔ ***Which ones?*** ________________________________
- ❑ 2 No

3. Does your clinic's mission include offering services to patients with specific diseases *(e.g. diabetes, STD, COPD, etc.)*

- ❑ 1 Yes ➔ ***What types of problems?*** ________________________________
- ❑ 2 No

4. Using the scale below, indicate how important the following goals are for your clinic: *Circle your answer choice.*

	**Less important**								**Most important**
a) Accessibility of services offered by the clinic	1	2	4	5	6	7	8	9	10
b) Ongoing relationship with patients	1	2	4	5	6	7	8	9	10
c) Services that meet patients' physical, psychological and social needs	1	2	4	5	6	7	8	9	10
d) Delivery of prevention and health promotion services	1	2	4	5	6	7	8	9	10
e) Services that conform to established guidelines	1	2	4	5	6	7	8	9	10
f) Respect, courtesy and confidentiality	1	2	4	5	6	7	8	9	10
g) Equity in health care service delivery and absence of discrimination towards individuals	1	2	4	5	6	7	8	9	10

5. Do you *totally agree, partly agree, partly disagree, or totally disagree* with the following statements:

	**Totally agree**	**Partly agree**	**Partly disagree**	**Totally disagree**
a) Physicians see the clinic as a business for which financial return is important	4	3	2	1
b) Clinic physicians should consider environmental or occupational causes when assessing patients' health problems	4	3	2	1
c) Clinic physicians should consider social problems in their clinical interventions for patients *(e.g. poverty, violence, substance dependence)*	4	3	2	1
d) Clinic professionals share the clinic's mission, values and objectives. **If you are a "solo practice physician" ➡ *Go to question 6***	4	3	2	1
e) It is important for the clinic's general practitioners to work as a team	4	3	2	1

6. Circle the number (one only) on the scale below that corresponds best to your clinic's medical team's views of … *If you are a "solo practice physician", answer for yourself.*

a) "responsibility for health"

**(1) Health is an individual responsibility**	**(2) Health is a collective responsibility**

5---------------4----------------3---------------2---------------1---------------0---------------1---------------2---------------3---------------4--------------5

(1) Individual responsibility: it is up to each individual to maintain his or her health or do what it takes to improve his or her health.
(2) Collective responsibility: it is up to society to create conditions that help maintain or improve health.

b) the "right to services"

(3) Access to care is an absolute right	(4) Access to care is a relative right

5---------------4----------------3---------------2---------------1---------------0---------------1---------------2---------------3---------------4---------------5

(3) Access to care is an absolute right: everyone should have the same access to health care based on need, regardless of financial ability to pay.
(4) Access to care is a relative right: everyone should have access to health care but people who can afford it could pay for better access to health care.

7. Choose the one statement that corresponds best to your clinic's priorities regarding service organization.

- ❑ 1 Service accessibility is a higher priority
- ❑ 2 Continuity of care for patients is a higher priority

Questions in section d are about the location of your primary healthcare clinic and other resources found in the same building

Section D: Clinic location

1. How long has your clinic been in operation?

- ❑ 1 Less than 1 year
- ❑ 2 1 to 5 years
- ❑ 3 Over 5 years

2. Where is your clinic located?

- ❑ 1 In a building owned by the physicians or of which they are shareholders
- ❑ 2 In rented offices in a commercial building for health professionals
- ❑ 3 In rented offices in a commercial building for any type of business
- ❑ 4 In a pharmacy
- ❑ 5 In an establishment that is part of the publicly-funded health network *(hospital, CLSC, etc.)*
- ❑ 97 Others ➔ ***Specify:*** ______________________________

3. Other than your clinic, are there other <u>primary healthcare medical teams</u> or other <u>general practitioners</u> in the building?

- ❑ 1 Yes
- ❑ 2 No ➔ ***Go to question 6***

4. Please specify these other primary healthcare medical teams or general practitioners:

Name of the physician-in-charge OR of a physician who could answer for the team	Number of physicians

5. Do the general practitioners at your clinic share any of the following with one of these teams …

a) parts of the premises *(offices or examination rooms)?* ❑ 1 Yes ❑ 2 No
b) the operating costs of the clinic? ❑ 1 Yes ❑ 2 No
c) support staff *(secretary and receptionist)?* ❑ 1 Yes ❑ 2 No
d) an appointment management system? ❑ 1 Yes ❑ 2 No
e) a medical records system? ❑ 1 Yes ❑ 2 No

6. In the building in which your clinic is located, are there any services offered by medical specialists?

❑ 1 Yes
❑ 2 No ➔ ***Go to question 9***

7. What are these physicians' specialties?

____

____

____

____

8. Do the general practitioners at your clinic refer patients to the medical specialists in your building?

❑ 1 Yes
❑ 2 No

9. In the building in which your clinic is located, are there any services offered by other health professionals?

❑ 1 Yes
❑ 2 No ➔ ***END OF THE QUESTIONNAIRE***

10. To which professional groups do they belong?

❑ a Pharmacists
❑ b Osteopaths, chiropractors, acupuncturists, etc.
❑ c Nutritionists
❑ e Social workers
❑ f Community organizers
❑ g Others ➔ ***Specify:*** ______________________________

____

11. Does your primary healthcare medical team refer patients to these other health professionals?

❑ 1 Yes
❑ 2 No

THANK YOU FOR YOUR COLLABORATION!

If you have any additional comments, please write them down in the space provided below.
Your observations are important to us.

DATE: …..…..…/……...…/………..
(day /month /year)
