# Supplementary material for: Constructing Taxonomies to Identify Distinctive Forms of Primary Healthcare Organizations
Source: ISRN Family Med. 2013 Apr 15;2013:798347. doi: 10.5402/2013/798347 (PMC4041222; doi:10.5402/2013/798347)
Supplement: Supplementary file 1 — Research report (46) and Organizational questionnaire developed for mail organizational survey of general practitioners' PHC practice settings [file 798347.f1.zip › Supplementary Material/Hamel_et_al_2012_BMC_HSR_Appendix_1_revised_final_ISRNFM_477957.doc]

**Appendix 1: Organizational indicators**

**Vision**

**Variables and categories (% of 473 organizations)	Questions – sources (Pineault et al, 2009; Hamel et al, 2007)**

| **V1 - Scope of responsibility**  1. More socio-environmental (63,2)  2. Less socio-environmental (36,8) | **Do you *totally* *agree, partly agree, partly disagree or totally disagree* with the following statements:**  Clinic physicians should consider environmental or occupational causes when assessing patients’ health problems  Clinic physicians should consider social problems in their clinical interventions for patients (e.g. poverty, violence, substance dependence) |
| --- | --- |
| **V2 - Responsibility**  1. Population (12,5) 2. Clientele or regular clients (75,1) 3. Individuals who present (12,5) | **Which statement best represents the population that your clinic tries to serves?**  *The population in neighbourhood, village or territory served by the clinic / Regular clinic patients registered at the clinic / Anyone who needs services and shows up at the clinic* |
| **V3 - Organizational priority**  1. Continuity of care (79,7) 2. Accessibility services (20,3) | **Chose the one statement that corresponds best to your clinic’s priorities regarding service organization?**  *Services accessibility is a higher priority / Continuity of care for patients is a higher priority* |
| **V4 - Importance attached to experience of care**  1. More important (33,2) 2. Moderately important (35,3) 3. Less important (31,5) | **Using the scale below, indicate how important the following goals are for your clinic:**  Accessibility of services offered by the clinics  Ongoing relationship with patients  Services that meet patents’ physical, psychological and social needs  Delivery of prevention and health promotion services  Services that conform to established guidelines  Respect, courtesy and confidentiality  Equity in health care service delivery and absence of discrimination towards individuals |
| **V5 - Right and responsibility regarding health and access to services**  1. More social (44,2) 2. Moderately social (36,8) 3. Less social (31,5) | **Which statement corresponds best to your clinic’s medical team’s views of “responsibility for health”?**  *Health is an individual responsibility / Health is a collective responsibility*  **Which statement corresponds best to your clinic’s medical team’s views of “right to services”?**  *Access to care is an absolute right / Access to care is a relative right* |
| **V6 - Financial return**  1. Less important (47,6) 2. More important (52,4) | **Do you *totally agree, partly agree, partly disagree or totally disagree* with the following statements:**  Physicians see the clinic as a business for which financial return is important |
| **V7 - Team work**  1. More important (48,6) 2. Less important (51,4) | **Do you *totally agree, partly agree, partly disagree or totally disagree* with the following statements:**  Clinic professionals share the clinic’s mission, values and objectives  It is important for the clinic’s general practitioners to work as a team |
| **V8 - Accountability to managers and colleagues**  1. More important 2. Less important | **To what extent do general practitioners at your clinic feel accountable to...**  Clinic’s managers? *Highly / Fairly / Slightly / Not at all*  Colleagues at work? *Highly / Fairly / Slightly / Not at all* |
| **V9 - Accountability to RAMQ and CMQ**  1. More important (68,9) 2. Less important (31,1) | **To what extent do general practitioners at your clinic feel accountable to…**  The Collège des médecins? *Highly / Fairly / Slightly / Not* at all  The RAMQ? *Highly / Fairly / Slightly / Not at all* |

Resources

| **R1 - Size of clinics (number of FTE practitioners)**  1. > 6 FTE (Large) (19,5)  2. 4-6 FTE (Average) (20,5) 3. 2-3 FTE (Small) (23,5)  4. ≤ 1 FTE (Very small) (36,6) | **How many general practitioners work at your clinic in the following categories:**  Less than 10 hours a week? ____ 26 to 40 hours a week? ____  10 to 25 hours a week?____ More than 40 hours a week? ____ |
| --- | --- |
| **R2 - Presence of nurses**  1. Yes (36,6) 2. No (63,4) | **How many nurses work at your clinic?** ____ |
| **R3 - Presence of other health professional or medical specialists**  1. Specialists with/ without other (High)(52,20 2. Health professional only (Average)(26,2) 3. None (21,6) | **In the building in which your clinic is located, are there any services offered by medical specialists?** *Yes / No*  **In the building in which your clinic is located, are there any services offered by other health professionals*?*** *Yes / No* |
| **R4 - Financing source for the operating costs of your clinic**  1. Public source (11,4) 2. Physicians and public source (7,4) 3. Physicians source only (81,2) | **Does the financing for the operating costs of your clinic come from…**  Fees charged to physicians or contributions by physicians? *Yes / No*  An institutional operating budget *(CLSC, hospital, etc*)? *Yes / No* |
| **R5 - Financial contribution from patients or enterprises**  1. No (87,9) 2. Yes (12,1) | **Does the financing for the operating costs of your clinic come from…**  Private enterprises *(companies, pharmacies, donations, foundations, etc.)* *Yes / No*  Fees charged to patients *(e.g. fees to open or manage files)*? *Yes / No* |
| **R6 - Information technologies**  1. 3 technologies or more (High) (18,0) 2. 2 technologies (Average) (24,9) 3. 1 technology (Low)(27,3) 4. None (Very low) (29,8) | **Do you have at your clinic…**  Computer software to mange appointments? *Yes / No*  Electronic medical records? *Yes / No*  Internet access (Web) for physicians? *Yes / No*  Access to the health and social service telecommunications network (RTSS)? *Yes / No*  An electronic system to transmit prescriptions to pharmacies? *Yes / No* |
| **R7 - Technical platform**  1. Radiology with or without biological specimen collection (High) (10,4)  2. Biological spec collect only (Average) (39,5)  3. None (Very low) (50,1) | **At your clinic, where do you send patients for the following services**  Biological specimen collection?  *On site / To another PH clinic / To a CLSC / To a private laboratory or radiology clinic / To an hospital*  Radiology?  *On site / To another PH clinic / To a CLSC / To a private laboratory or radiology clinic / To an hospital* |

Structure

| **S1 - Governance**  1. Public (11,6) 2. Professional – private (88,4) | **Is it a…** *medical clinic / a CLSC / a UMF*? |
| --- | --- |
| **S2 - Medical and administrative management**  1. Administrator manager and physicians (13,5) 2. Responsible physician or group (42,7)  3. Not specified and solo practice (43,8) | **At your clinic, who…**  Sets up on-call lists, schedules, vacations, etc.?  *Physician-in-charge or designated physicians / Physicians group collectively / Administrator or manager / None*  Ensures that the quality of medical acts is evaluated?  *Physician-in-charge or designated physicians / Physicians group collectively / Administrator or manager / None*  Represents the clinic on committees?  *Physician-in-charge or designated physicians / Physicians group collectively / Administrator or manager / None* |
| **S3 - Seniority of physicians**  1. > 5 years (77,0) 2. ≤ 5 years (23,0) | **How many of these general practitioners have been working at your clinic for more than 5 years?**  *All or most / Some / None* |
| **S4 - Implication of the physician in the clinic**  1. High (58,6) 2. Low (41,4) | **In all, how many general practitioners work at your clinic? ____**  **How many general practitioners work at your clinic more than 26 hours a week? ____** |
| **S5 - Mode of remuneration (payment of physicians)**  1. Fee-for-service (88,4) 2. Time based (11,6) | **On the whole, how are general practitioners paid for activities conducted at your clinic?**  *Fee-for-services / Session fees (vacation) / Fixed honorarium / Various modes of payment* |
| **S6 - Administrative resources sharing**  1. High (31,1) 2. Average (31,5)  3. Low (37,4) | **How many general practitioners at your clinic share…**  Rooms (offices or examination rooms)? *All or most / Some / None*  Operating costs for the clinic? *All or most / Some / None*  Support staff (secretary and receptionist)? *All or most / Some / None*  An appointment management system*? All or most / Some / None*  A medical records system*? All or most / Some / None*  Pooled incomes? *All or most / Some / None* |
| **S7 - Coordination of care (intra-organizational)**  1. Formal (27,7) 2. Informal (30,0) 3. None (42,3) | **How is care coordinated among clinic professionals?**  Informal or ad hoc exchanges? *Always / Often / Sometimes / Never*  Pre-established care protocols for specific client groups or problems*? Always / Often / Sometimes / Never*  Case discussion meetings? *Always / Often / Sometimes / Never* |
| **S8 - Collaboration with PHC organizations**  1. Yes (49,7) 2. No (50,3) | **Indicate whether your clinic has *formal or informal arrangements* with one or several PHC medical clinic(s) for the following activities:**  Planning services offer (on-call activities, clinic hours, walk-ins services etc.?  Access to technical services (e.g. radiology, laboratory)?  Exchange resources (e.g. loan of professionals)?  Referral or transfer of patients to general practitioners, specialists or other professionals?  Follow-up for hospitalised patients or patients seen at the clinic? |
| **S9 - Collaboration with secondary care institutions**  1. Yes (48,8)  2. No (51,2) | **Indicate whether your clinic has formal or informal arrangements with one or several hospital(s) for the following activities:**  Planning services offer (on-call activities, clinic hours, walk-ins services etc.? *Yes / No*  Access to technical services (e.g. radiology, laboratory)? *Yes / No*  Exchange resources (e.g. loan of professionals)? *Yes / No*  Referral or transfer of patients to general practitioners, specialists or other professionals? *Yes / No*  Follow-up for hospitalised patients or patients seen at the clinic? *Yes / No* |
| **SA - Participation of physicians in regional committees**  1. Yes (40,2) 2. No (59,8) | **How many physicians at your clinic participate in the following activities:**  Local committees of the “Direction régionale de medicine générale” (DRMG)? *All or most / Some / None*  Committees for the implementation of Family Medicine Groups (FMG)? *All or most / Some / None*  Committees to alleviate congestion in emergency departments? *All or most / Some / None*  Committees on the health and social service centre’s (CSSS) clinical project? *All or most / Some / None* |

Practices

| **P1 - Availability of services**  1. High (16,1) 2. Average (24,5) 3. Low (59,4) | At your clinic, do you offer services **by appointment** during weekends (Saturday or Sunday)? *Yes / No*  At your clinic, do you offer **walk-in** services during weekends (Saturday or Sunday*)? Yes / No*  At your clinic, do you offer services **by appointment** during weekday evenings (after 6:00 p.m.)? *Yes / No*  At your clinic, do you offer **walk-in** services during weekday evenings(after 6:00 p.m.)? *Yes / No* |
| --- | --- |
| **P2 - Availability in case of emergency**  1. High (11,4) 2. Average (55,2)  3. Low (33,4) | **When your clinic is closed, is there an on-call system for your regular patients?** *Yes / No*  **For regular patients of the clinic, indicate how often patients would be able to contact a physician or nurse by telephone should they get sick during the clinic’s opening hours.** *Always / Often / Sometimes / Never* |
| **P3 - Participation in a healthcare access network**  1. Yes (9,3) 2. No (90,7) | **Does your clinic participate in a healthcare access network to ensure that your clinic’s office hours are coordinated with those of other clinics?** *Yes / No* |
| **PZ - Mode of consultation**  1. Mostly scheduled appointment (61,7) 2. Mixed (15,6) 3. Mostly walk-in (22,6) | **Among all consultations at the clinic, what is the percentage for walk-in clinics?**  *0% / 1 to 25% / 26 to 50% / 51 to 75% / 76 to100%* |
| **P5 - Others settings for clinic’s services**  1. 2-3 settings (21,6)  2. 1 setting (23,0)  3. None (55,4) | **At your clinic, to what extent are the following services available:**  Medical or nursing care and services at a patient’s home? *Very / Moderately / A little / Not*  Medical or nursing care and services in homes for senior, nursing homes *Very / Moderately / A little / Not*  Hospital care*? Very / Moderately / A little / Not* |
| **P6 - Approximate time for a consultation**  1. Long (30,4) 2. Average (31,9)  3. Short (37,6) | **At your clinic, how much time is scheduled for visits for a complete examination or evaluation of a new patient?**  *Less than 10 minutes / 10 minutes / 15 minutes / 20 minutes / 30 minutes / More than 30 minutes*  **At your clinic, how much time is scheduled for follow-up visits or emergency consultations?**  *Less than 10 minutes / 10 minutes / 15 minutes / 20 minutes / 30 minutes / More than 30 minutes* |
| **P7 - Scope of services**  1. Very Broad (27,3)  2. Broad (30,2)  3. Narrow (42,5) | **Are the following diagnostic and therapeutic procedures available on-site?** *Yes / No*  ECG interpretation? interpretation of simple radiological films?  capillary glycaemia and cholesterol? IUD insertion?  needle aspiration? musculo-skeletal injection?  strep test? casting or splinting?  skin biopsy? minor surgery or suture?  **Are the following preventive services part of your clinic’s usual practices:** *Yes / No*  identification of drugs or alcohol problems? prevention of osteoporosis?  screening for hypercholesterolemia? influenza vaccination?  clinical breath examinations? counselling for smoking cessation?  mammography? counselling for maintaining a healthy weight?  cervical smear? prevention of falls in the elderly?  **At your clinic, to what extent are the following services available: Palliative care***? Yes/ No* |
| **P8 - Number of diagnostic and therapeutic procedures**  1. More / 7-10 (27,9) 2. Average / 4-6 (31,3) 3. Less / 0-3 (40,8) | **Are the following diagnostic and therapeutic procedures available on-site?** *Yes / No*  ECG interpretation? interpretation of simple radiological films?  capillary glycaemia and cholesterol? IUD insertion?  needle aspiration? musculo-skeletal injection?  strep test? casting or splinting?  skin biopsy? minor surgery or suture? |
| **P9 - Management of care for patients with chronic disease**  1. More (33,8) 2. Less (66,2) | **At your clinic, do you offer specific case management and systematic approaches to care for people with chronic diseases**  *Never / Sometimes / Often / Always* |
| **PA - Support for the appointment**  1. More (35,7)  2. Less (64,3) | **Does your clinic confirm appointments with patients a few days before a scheduled visit?** *Always / Often / Sometimes / Never*  **In general, appointments with specialists are made by…**  *The patient / The clinic support staff / The nurse / the physician* |
| **PB - Role of the nurses**  1. Extended role (innovated) (22,4) 2. Limited role (traditional) (14,2) 3. No nurse in the clinic (63,4) | **What are the roles and functions of the nurses on your medical team? *Check all that apply***  *There is no nurse on our team Triage of walk-in patients*  *Prevention, promotion and screenings activities Health education*  *Systematic approaches to care (application of care protocols) Participation in clinical decisions*  *Liaison and coordination with CLSC, LTCF, hospitals or others clinics*  *Support for medical activities (blood pressure, weight, injections, etc.)* |
| **PC - Sharing of clinical activities between physicians**  1. High (49,9) 2. Low (50,1) | **How many general practitioners at your clinic share …**  Coverage of walk-in clinic periods? *All or Most / Some/ None*  Coverage of scheduled appointment periods? *All or Most / Some/ None*  In-hospital care for the clinic’s patients? *All or Most / Some/ None*  Patient follow-up? *All or Most / Some/ None*  Replacement for a physician absent from the clinic? *All or Most / Some/ None* |
| **PD - Network to refer patient to others PHC clinics**  1. High (70,6) 2. Low (29,4) | **At your clinic, where do you send patients for the following services: biological specimen collection and radiology**  To another PHC clinic? *Yes / No* |
| **PE - Network to refer patient to hospital/specialist**  1. High (55,2) 2. Low (44,8) | **At your clinic, where do you send patients for the following services: biological specimen collection and radiology**  To a hospital? *Yes / No*  **Where are patients sent when they need to see specialists?**  To hospital or hospital out-patient clinics? *Alway*s */ Often / Sometimes / Never*  To hospital emergency rooms? *Always / Often / Sometimes / Never* |
| **PF - Network to refer patient to private sector**  1. High (76,1) 2. Low (23,9) | **At your clinic, where do you send patients for the following services: biological specimen collection and radiology**  To a private laboratory? *Yes / No*  To a private radiology clinic? *Yes / No*  **Where are patients sent when they need to see specialists?**  To specialists’ private offices? *Always / Often / Sometimes / Never* |
| **PG - Organizational affiliation of the physicians**  1. Double affiliation (44,6) 2. Single affiliation (40,0) 3. None (15,4) | **In addition to practicing at your clinic, how many of the general practitioners also provide care in the following other health settings or programs:**  In the emergency room of a general and/or specialized care hospital? *All or Most / Some/ None*  In a short-term care unit of a general and specialized care hospital? *All or Most / Some/ None*  In a long-term care facility (CHSLD)? *All or Most / Some/ None*  Obstetrical services in a hospital? *All or Most / Some/ None*  In another PHC clinic or CLSC? *All or Most / Some/ None* |
| **PH – Quality assessment (existence of mechanisms for maintenance or evaluation professional)**  1. More (20,5) 2. More or less (33,8)  3. None (45,7) | **At your clinic, who…**  Looks after recruitment of physicians and assigns practice privileges?  *Physician-in-charge or designated physicians / Physicians group collectively / Administrator or manager / None*  Ensures that the quality of medical acts is evaluated?  *Physician-in-charge or designated physicians / Physicians group collectively / Administrator or manager / None*  Organises continuing medical education activities?  *Physician-in-charge or designated physicians / Physicians group collectively / Administrator or manager / None* |
